# Speech therapy for poststroke aphasia: a network meta-analysis of randomized controlled trials

**DOI:** 10.7717/peerj.21118

**Published:** 2026-04-15

**Authors:** Fu-An Yang, Chao-Chun Huang, Chao-Hsien Lu, Pei-Jyuan Wu, Reuben Escorpizo, Hung-Chou Chen

**Affiliations:** 1 Department of Physical Medicine and Rehabilitation, Far Eastern Memorial Hospital, New Taipei City, Taiwan; 2 Department of Internal Medicine, China Medical University Hospital, Taichung, Taiwan; 3 School of Medicine, College of Medicine, Taipei Medical University, Taipei, Taiwan; 4 Department of Physical Therapy and Assistive Technology, National Yang Ming Chiao Tung University, Hsinchu City, Taiwan; 5 Department of Physical Medicine and Rehabilitation, Shuang Ho Hospital, Taipei Medical University, Taipei, Taiwan; 6 Department of Rehabilitation and Movement Science, University of Vermont, College of Nursing and Health Sciences, Burlington, United States; 7 Swiss Paraplegic Research, Nottwil, Switzerland; 8 Department of Physical Medicine and Rehabilitation, School of Medicine, College of Medicine, Taipei Medical University, Taipei, Taiwan; 9 Center for Evidence-Based Health Care, Shuang Ho Hospital, Taipei Medical University, Taipei, Taiwan

**Keywords:** Aphasia, Constraint-induced aphasia therapy, Network meta-analysis, Multimodality aphasia therapy

## Abstract

**Objective:**

Various speech therapies are available for treating poststroke aphasia; however, the effects of these strategies on poststroke aphasia have yet to be compared. We conducted a network meta-analysis to investigate the effects of different speech therapies on quality of life and language performance for patients with poststroke aphasia.

**Methods:**

This systematic review and network meta-analysis was registered in the PROSPERO database (CRD42023465936) on October 2, 2023. We searched the PubMed, EMBASE, and Cochrane Library electronic databases from their inception to September 20, 2023. We included trials that (1) involved participants with poststroke aphasia regardless of phase; (2) adopted speech therapy as the intervention; (3) applied either no intervention or another speech therapy as the control treatment; (4) reported quality of life or language performance as outcomes. The network meta-analysis was performed using the online tool ShinyNMA (version 1.01).

**Results:**

We examined 17 articles involving 931 patients. Our analyses revealed that both multimodality aphasia therapy and constraint-induced aphasia therapy prompted significant improvements in quality of life. However, no specific speech therapy showed statistically significant superiority over no intervention across isolated language domains. All significant results were also clinically meaningful. No significant inconsistencies were observed between the results of direct and indirect comparisons.

**Conclusions:**

Our results suggest that multimodality aphasia therapy and constraint-induced aphasia therapy show promise for improving quality of life in patients with poststroke aphasia. However, no single speech therapy demonstrated statistical superiority over no intervention across specific language domains.

## Introduction

Stroke is a syndrome characterized by acute, focal neurological deficits caused by vascular damage to the central nervous system, often resulting in physical disabilities (Kim & Yang, 2022; Murphy & Werring, 2020). Among these, poststroke aphasia is a common and impactful condition—a language disorder that impairs communication, affecting speaking, understanding, reading, and writing (Berthier, 2005; Fridriksson & Hillis, 2021; Pedersen, Vinter & Olsen, 2004). Typically caused by damage to language-related areas in the left hemisphere of the brain, aphasia significantly reduces a person’s ability to engage in daily activities and diminishes quality of life (Berthier, 2005; Fridriksson & Hillis, 2021; Pedersen, Vinter & Olsen, 2004). Poststroke aphasia occurs in approximately 21% to 38% of individuals with acute stroke, underscoring its prevalence and clinical importance (Berthier, 2005; Fridriksson & Hillis, 2021; Pedersen, Vinter & Olsen, 2004).

Although spontaneous recovery may occur within the first 3 to 6 months in some cases, speech therapy remains necessary. Such therapy has been demonstrated to yield clinically and statistically significant improvements in the language abilities of patients (Brady et al., 2016; Burton et al., 2023; Brady et al., 2022; Vitti & Hillis, 2021). Common training strategies for aphasia implemented in clinical practice include melodic intonation therapy (MIT), constraint-induced aphasia therapy (CIAT), promoting aphasics’ communicative effectiveness (PACE), and multimodality aphasia therapy (M-MAT) (Brady et al., 2022; Wortman-Jutt & Edwards, 2019). Other approaches such as computerized speech and language therapy (CSLT) and cognitive-linguistic treatments like BOX therapy have also been developed and will be included in our analysis. MIT is a rehabilitation technique that uses the melodic and rhythmic elements of music to improve speech production in individuals with post-stroke aphasia (Norton et al., 2009). A meta-analysis published in 2022 highlighted the efficacy of MIT in improving everyday communication skills (Popescu et al., 2022). CIAT is a speech rehabilitation approach that involves intensive practice of verbal communication while restricting nonverbal forms of communication to encourage the use of impaired language skills (Balardin & Miotto, 2009). Another study concluded that CIAT is beneficial for chronic poststroke aphasia (Zhang et al., 2017).

Although these methods yield improvements in functional communication, no study has compared all of these strategies. Therefore, we performed a comprehensive literature review to identify randomized controlled trials (RCTs) related to speech therapy for patients with poststroke aphasia; we then conducted a network meta-analysis to compare the effectiveness of these therapies in improving the quality of life and language functions of patients with poststroke aphasia.

Our research questions were as follows:

1. What are the outcomes of different speech therapies in patients with poststroke aphasia, in comparison to no intervention or other active therapies?
2. Which therapy is the most effective in improving quality of life and specific language domains, as measured by the standardized mean difference (SMD) synthesized from RCTs?

## Materials and Methods

This systematic review and network meta-analysis was registered in the International Prospective Register of Systematic Reviews (PROSPERO) database (CRD42023465936) on October 2, 2023. The study was performed in accordance with the recommendations of the Preferred Reporting Items for Systematic Reviews and Meta-Analyses (PRISMA) extension statement for network meta-analyses (Hutton et al., 2015). Portions of this text were previously published as part of a preprint (https://www.researchsquare.com/article/rs-4312160/v1) (Yang et al., 2024).

We included RCTs, including those with parallel, pilot, or crossover research designs. We used the following patient (P), intervention (I), comparison (C), and outcome (O) criteria to identify eligible studies:

(P) involved participants with poststroke aphasia regardless of phase;

(I) applied speech therapy as the intervention;

(C) adopted either no intervention or another speech therapy as the control treatment;

(O) reported quality of life or language performance (fluency, comprehension, repetition, or naming) as outcomes.

We excluded protocol articles, articles that were not peer reviewed, conference articles, and letters to the editor. We applied no language restrictions in our search strategy.

Each author independently reviewed the literature, extracted data, and performed crosschecks following the PRISMA guidelines (Page et al., 2020). We searched the PubMed, EMBASE, and Cochrane Library electronic databases from their inception to September 20, 2023. In our search strategy, we included terms related to poststroke aphasia and speech training and other equivalent terms (Supplemental Appendix). RCTs were identified using the refined search function of the databases, if available. Additional articles were identified by manually searching the reference lists of relevant articles. Two reviewers independently evaluated the eligibility of all titles and abstracts, and disagreements were resolved through discussion with a third reviewer. Subsequently, the full texts of all remaining articles were screened to determine their eligibility.

Two authors (Fu-An Yang and Chao-Chun Huang) independently extracted the following information from each article: type of RCT; inclusion criteria; number, mean age, and time since aphasia onset of participants; the protocol used in different groups; follow-up duration; and outcome measurements. Discrepancies were identified and resolved through discussion with the third reviewer (Hung-Chou Chen). Unclear or missing data were requested from the article authors by email.

We extracted data for five specific outcomes: quality of life (*e.g*., SAQOL-39), and four language domains based on standardized aphasia batteries: fluency, comprehension, repetition, and naming. To minimize the confounding effect of spontaneous recovery, which predominantly occurs within the first 6 months post-stroke (Chiaki & Shinichiro, 2021), we conducted a pre-specified subgroup analysis including only studies that recruited participants in the chronic phase (>6 months post-onset). Outcomes assessed at the end of the intervention were collected for analysis.

The quality of the included studies was assessed using the Physiotherapy Evidence Database (PEDro) scale, a reliable quality assessment tool for evaluating bias risk in RCTs (Maher et al., 2003); inconsistencies in quality scores between the two authors (Fu-An Yang and Chao-Chun Huang) were resolved through discussion with the third reviewer (Hung-Chou Chen). Ratings of PEDro scale items 2–11 are summed to obtain a total score between 0 and 10. Scores of <4, 4 to 5, 6 to 8, and 9 to 10 are considered poor, fair, good, and excellent, respectively (Maher et al., 2003). All articles were included in this review irrespective of their PEDro score.

A Network Meta-Analysis is a statistical method used to compare three or more interventions simultaneously by integrating both direct (head-to-head comparisons within trials) and indirect (comparisons across trials through a common comparator) evidence across a network of studies (Higgins et al., 2023). This approach provides estimates of the relative effects between any pair of interventions in the network and typically yields more precise results than individual direct or indirect analyses (Higgins et al., 2023). In our network construction, “no intervention” and “conventional therapy” were treated as separate nodes to distinguish between the total absence of speech therapy (or waitlist control) and standard clinical care. Furthermore, Network Meta-Analysis allows for ranking the interventions based on their relative effectiveness, offering valuable insights into the comparative performance of different treatments (Higgins et al., 2023). For our analysis, we used ShinyNMA (version 1.01; https://jerryljw.shinyapps.io/ShinyNMA_/) (Wei-Ming, Ren-wei & Shen-hua, 2021), a free online cloud computing network meta-analysis tool. ShinyNMA can be used to draw charts as per the standards of the latest PRISMA 2020 guidelines (Page et al., 2020). It also synthesizes results and guides the selection of R software (version 4.1.0) packages between metafor (version 2.4-0), netmeta (version 1.3-0), and BUGSnet (version 1.0-4).

We extracted continuous data by changing the baseline measurement. For studies that reported outcome data at baseline and follow-up but did not report the standard deviation (SD) of the change score, we calculated the SD using methods described in the Cochrane Handbook for Systematic Reviews of Interventions (Higgins et al., 2023). Specifically, we imputed the change SD by assuming a conservative correlation coefficient (r) of 0.5 between the baseline and final measurements (Higgins et al., 2023). We employed a random-effects model and conducted head-to-head comparisons of different speech therapies for poststroke aphasia by estimating the standard mean difference (SMD) with its 95% confidence interval (CI). We also examined the distribution of probabilities of effectiveness for each speech therapy. For ranking the treatments, we employed the P score, which measures the extent of certainty that a treatment is superior to other treatments, averaged over all competing treatments (Rücker & Schwarzer, 2015). The P score ranges from 0 (*poorest*) to 1 (*best*). Therefore, a high P score indicates that the certainty that one treatment is superior to another is high. Furthermore, we examined network inconsistency by using loop-specific heterogeneity and local incoherence estimates and by comparing differences in effect sizes between standard meta-analyses (direct comparisons) and indirect comparisons (Higgins et al., 2023). SMDs calculated using Cohen’s d statistic were employed to measure the probable clinical meaningfulness of the relationships between variables in a population; an SMD of <0.2, of 0.2 to 0.5, of 0.5 to 0.8, and of >0.8 indicate clinically meaningless, small, moderate, and large effects, respectively (Cohen, 1988).

The certainty of evidence was assessed using CINeMA (Confidence in Network Meta-Analysis, https://cinema.ispm.unibe.ch/#), a tool based on the GRADE framework that evaluates six domains: (i) within-study bias (impact of risk of bias in the included studies), (ii) reporting bias (including publication and other reporting biases), (iii) indirectness, (iv) imprecision, (v) heterogeneity, and (vi) incoherence (Nikolakopoulou et al., 2020). Each domain is assessed at three levels of concern (no concerns, some concerns, or major concerns), with reviewer input required at the study level for within-study bias and indirectness. The judgments across these domains are summarized to determine four levels of confidence for each relative treatment effect, corresponding to the standard GRADE assessments of very low, low, moderate, or high (Nikolakopoulou et al., 2020).

## Results

### Search results

**Figure 1:**
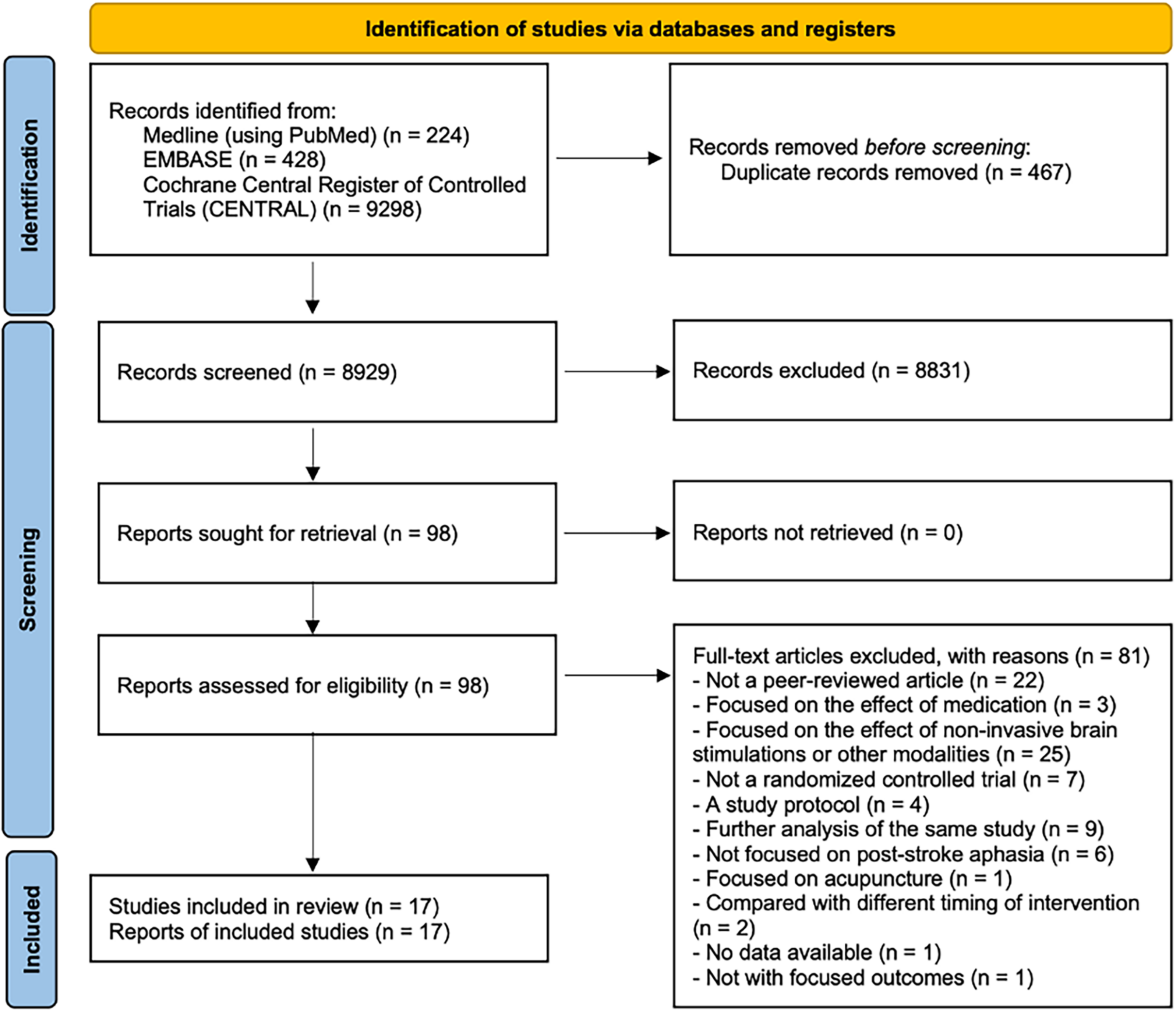
Article selection process.

We initially retrieved 9,950 records and excluded 1,021 duplicates. After title and abstract screening, we excluded a further 8,831 studies. The primary reasons for exclusion at this stage were: irrelevant topic (*e.g*., not focused on aphasia or speech therapy), non-RCT study design (*e.g*., case reports, reviews), and incorrect patient population (*e.g*., aphasia not caused by stroke). We screened the full texts of the remaining 98 studies and excluded 22 that were not peer reviewed, three that focused on the effects of medication, 25 that focused on the effect of noninvasive brain stimulations or other modalities, seven that were not RCTs, four that were study protocols, nine that were further analyses of the same study (we ensured that only the primary RCT data from these studies were included in our final analysis to avoid duplicating participants) (Palmer et al., 2020; Rose et al., 2022; Szaflarski et al., 2015; Van Der Meulen et al., 2016), six that did not focus on poststroke aphasia, one that focused on acupuncture, two that compared different timings of an intervention, one with data that could not be assessed and one not with focused outcomes. Finally, our network meta-analysis included 17 articles involving 931 participants (Fig. 1) (Rose et al., 2022; Szaflarski et al., 2015; Van Der Meulen et al., 2016; Ciccone et al., 2016; Kurland et al., 2016; Nenert et al., 2017; Palmer et al., 2019; Pierce et al., 2023; Raglio et al., 2016; Sickert et al., 2014; Spaccavento et al., 2021; Stahl et al., 2017; Vuksanović et al., 2018; Wilssens et al., 2015; Woldag et al., 2017; Zhang et al., 2021; Breitenstein et al., 2017).

### Study characteristics

Of the 17 included RCTs, 15 were parallel RCTs (Rose et al., 2022; Szaflarski et al., 2015; Van Der Meulen et al., 2016; Ciccone et al., 2016; Kurland et al., 2016; Nenert et al., 2017; Palmer et al., 2019; Pierce et al., 2023; Raglio et al., 2016; Sickert et al., 2014; Spaccavento et al., 2021; Wilssens et al., 2015; Woldag et al., 2017; Zhang et al., 2021; Breitenstein et al., 2017), and two were crossover studies (Stahl et al., 2017; Vuksanović et al., 2018). According to the Cochrane Handbook for Systematic Reviews of Interventions, including crossover studies in a network meta-analysis is acceptable (Higgins et al., 2023). For the included crossover trials, only data from the first period (pre-crossover) were extracted and analyzed. This approach was adopted in accordance with the Cochrane Handbook recommendations to treat the data as if from a parallel-group trial, thereby avoiding potential carryover effects (Higgins et al., 2023). The studies we reviewed employed the following speech therapies: CIAT (Rose et al., 2022; Szaflarski et al., 2015; Ciccone et al., 2016; Kurland et al., 2016; Nenert et al., 2017; Pierce et al., 2023; Sickert et al., 2014; Stahl et al., 2017; Vuksanović et al., 2018; Wilssens et al., 2015; Woldag et al., 2017), computerized speech and language therapy (CSLT) (Palmer et al., 2019; Spaccavento et al., 2021), MIT (Van Der Meulen et al., 2016; Raglio et al., 2016; Zhang et al., 2021), BOX therapy (Wilssens et al., 2015), M-MAT (Pierce et al., 2023), PACE (Kurland et al., 2016; Raglio et al., 2016), high-intensity speech and language therapy (HISLT) (Breitenstein et al., 2017) and conventional thearpy (Ciccone et al., 2016; Palmer et al., 2019; Sickert et al., 2014; Spaccavento et al., 2021; Stahl et al., 2017; Vuksanović et al., 2018; Woldag et al., 2017; Zhang et al., 2021); no intervention was prespecified as placebo. Specifically, the HISLT node represents data exclusively from the single large-scale FCET2EC trial (Breitenstein et al., 2017, *n* = 156). Rose et al. (2022) conducted a 3-arm study comparing CIAT, M-MAT, and no intervention (Rose et al., 2022). Twelve studies included patients who had poststroke aphasia for >6 months (Szaflarski et al., 2015; Van Der Meulen et al., 2016; Kurland et al., 2016; Nenert et al., 2017; Palmer et al., 2019; Pierce et al., 2023; Raglio et al., 2016; Stahl et al., 2017; Wilssens et al., 2015; Zhang et al., 2021; Breitenstein et al., 2017) and five studies <6 months (Ciccone et al., 2016; Sickert et al., 2014; Spaccavento et al., 2021; Vuksanović et al., 2018; Woldag et al., 2017). A summary of the main characteristics of the 17 included RCTs is presented in Table S1.

### Risk-of-bias assessment

The quality of the included RCTs was independently assessed using the PEDro scale. All included studies had PEDro scores between 6 and 9, with one study having excellent quality (Szaflarski et al., 2015), and the remaining 16 studies having good quality (Rose et al., 2022; Van Der Meulen et al., 2016; Ciccone et al., 2016; Kurland et al., 2016; Nenert et al., 2017; Palmer et al., 2019; Pierce et al., 2023; Raglio et al., 2016; Sickert et al., 2014; Spaccavento et al., 2021; Stahl et al., 2017; Vuksanović et al., 2018; Wilssens et al., 2015; Woldag et al., 2017; Zhang et al., 2021; Breitenstein et al., 2017). The detailed results of the bias risk assessment are listed in Table 1.

**Table 1:** PEDro scale evaluations.

	1*	2	3	4	5	6	7	8	9	10	11	Total	Rating
Breitenstein et al. (2017)	V	V	V	V			V	V	V	V	V	8	Good
Ciccone et al. (2016)	V	V	V	V			V	V		V	V	7	Good
Kurland et al. (2016)	V	V		V			V	V	V	V	V	7	Good
Nenert et al. (2017)	V	V		V			V	V	V	V	V	7	Good
Pierce et al. (2023)	V	V		V			V	V		V	V	6	Good
Rose et al. (2022)	V	V		V			V	V	V	V	V	7	Good
Sickert et al. (2014)	V	V		V			V	V		V	V	6	Good
Stahl et al. (2017)	V	V		V			V	V	V	V	V	7	Good
Szaflarski et al. (2015)	V	V	V	V		V	V	V	V	V	V	9	Excellent
Vuksanović et al. (2018)	V	V		V			V	V	V	V	V	7	Good
Wilssens et al. (2015)	V	V		V				V	V	V	V	6	Good
Woldag et al. (2017)	V	V		V			V	V	V	V	V	7	Good
Palmer et al. (2019)	V	V		V			V	V		V	V	6	Good
Spaccavento et al. (2021)	V	V		V				V	V	V	V	6	Good
Van Der Meulen et al. (2016)	V	V	V	V			V	V		V	V	7	Good
Raglio et al. (2016)	V	V		V			V	V	V	V	V	7	Good
Zhang et al. (2021)	V	V		V	V		V	V	V	V	V	8	Good

**Note:**

PEDro scale criteria: 1, eligibility criteria and source of participants; 2, random allocation; 3, concealed allocation; 4, baseline comparability; 5, blinded participants; 6, blinded therapists; 7, blind assessors; 8, adequate follow-up; 9, intention-to-treat analysis; 10, between-group comparisons; 11, point estimates and variability. *Not included in the calculation of the total score.

### Results of quality of life in all patients

**Figure 2:**
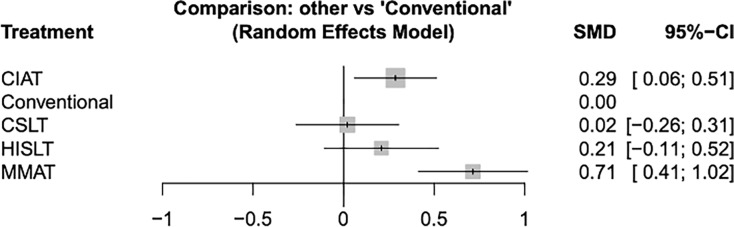
Forest plots of all results showing the quality of life of all patients. Abbreviations: 95% CI, 95% credible interval; CIAT, constraint-induced aphasia therapy; CSLT, computerized speech and language therapy; HISLT, high-intensity speech and language therapy; MMAT, multimodality aphasia therapy; SMD, standard mean difference.

**Figure 3:**
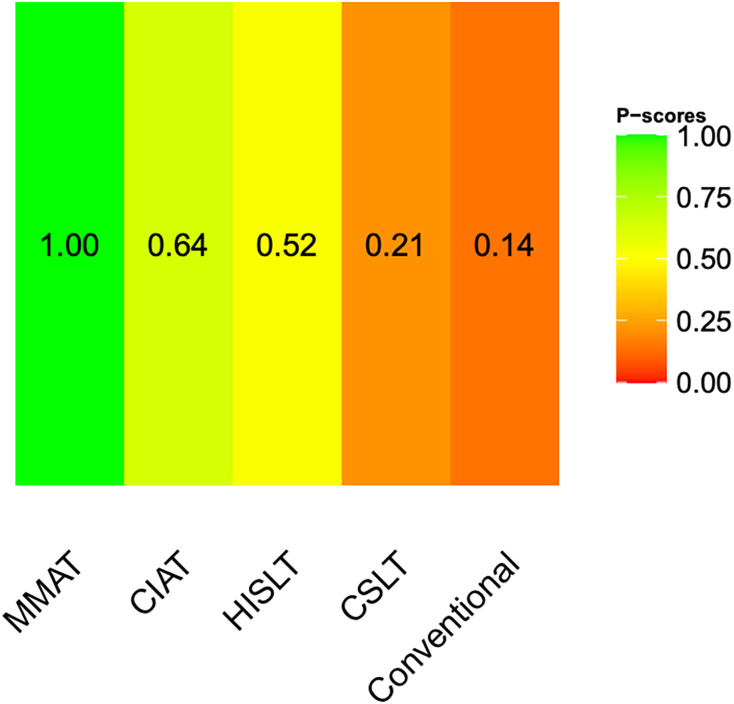
Distribution of probabilities of effectiveness for each speech therapy showing the quality of life of all patients. Abbreviations: CIAT, constraint-induced aphasia therapy; CSLT, computerized speech and language therapy; HISLT, high-intensity speech and language therapy; MMAT, multimodality aphasia therapy.

A network diagram of the included speech therapies in terms of the quality of life of the patients is given in Fig. S1. The pooled SMDs revealed that both MMAT (SMD = 0.71, 95% CI [0.41–1.02]) and CIAT (SMD = 0.29, 95% CI [0.06–0.51]) were significantly more effective than conventional therapy (Fig. 2). We synthesized head-to-head studies separately to assess the differences among speech therapies; the results of the pairwise meta-analysis and network meta-analysis are shown in Table 2. We also analyzed the distribution of probabilities of effectiveness for each speech therapy (Fig. 3). MMAT had the highest probability of being the most effective treatment, followed by CIAT, HISLT, CSLT, and conventional therapy.

**Table 2:** Network meta-analysis results for quality of life of all patients.

Pairwise meta-analysis				
**MMAT**	0.41 [0.10; 0.71]			0.77 [0.43; 1.11]
0.43 [0.14; 0.72]	**CIAT**			0.27 [0.05; 0.50]
0.51 [0.07; 0.94]	0.08 [−0.31; 0.47]	**HISLT**		0.21 [−0.11; 0.52]
0.69 [0.28; 1.11]	0.27 [−0.10; 0.63]	0.19 [−0.24; 0.61]	**CSLT**	0.02 [−0.26; 0.31]
0.71 [0.41; 1.02]	0.29 [0.06; 0.51]	0.21 [−0.11; 0.52]	0.02 [−0.26; 0.31]	**Conventional**
Network meta-analysis				
Data are expressed as SMD [95% CI]. Significant results are underscored. “-” means data not applicable.				

**Note:**

The table presents two types of estimates. Results in the upper-right triangle (above the gray diagonal) represent the SMD from direct (pairwise) meta-analyses. Results in the lower-left triangle (below the gray diagonal) represent the SMD from the final network meta-analysis, which incorporates both direct and indirect evidence. Abbreviations: 95% CI, 95% credible interval; CIAT, constraint-induced aphasia therapy; CSLT, computerized speech and language therapy; HISLT, high-intensity speech and language therapy; MMAT, multimodality aphasia therapy; SMD, standard mean difference.

Our network plot (Fig. S1) depicts comparisons centered around conventional therapy. The loop-specific heterogeneity assessment revealed no significant inconsistency between the results of direct trials and indirect comparisons (Table S2). We compared the differences between traditional pairwise meta-analyses and network meta-analyses, and the results (Fig. S2) revealed that none of the differences were significant.

### Results of quality of life in the chronic phase

**Figure 4:**
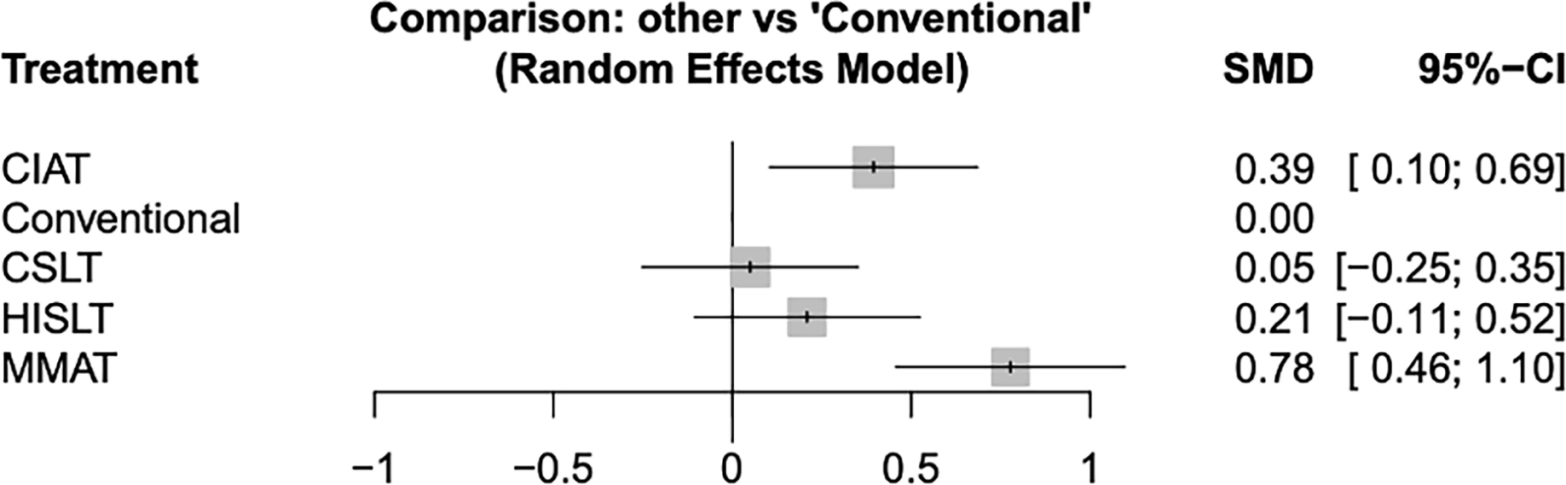
Forest plots of all results showing the quality of life of patients in the chronic phase. Abbreviations: 95% CI, 95% credible interval; CIAT, constraint-induced aphasia therapy; CSLT, computerized speech and language therapy; HISLT, high-intensity speech and language therapy; MMAT, multimodality aphasia therapy; SMD, standard mean difference.

**Figure 5:**
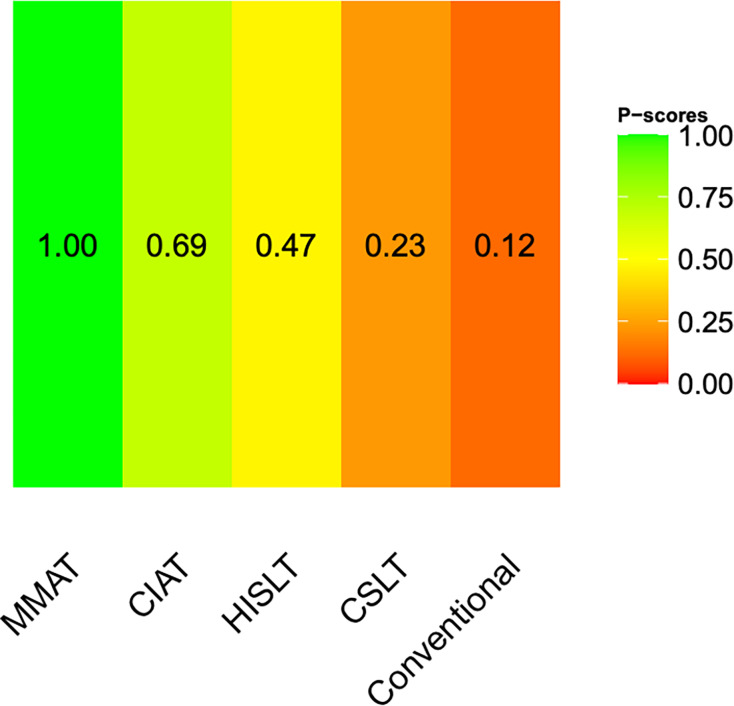
Distribution of probabilities of effectiveness for each speech therapy showing the quality of life of patients in the chronic phase. Abbreviations: CIAT, constraint-induced aphasia therapy; CSLT, computerized speech and language therapy; HISLT, high-intensity speech and language therapy; MMAT, multimodality aphasia therapy.

We conducted a subgroup analysis of patients in the chronic phase of aphasia. A network diagram of the included speech therapies is given in Fig. S3. The pooled SMDs revealed that both MMAT (SMD = 0.78, 95% CI [0.46–1.10]) and CIAT (SMD = 0.39, 95% CI [0.10–0.69]) were significantly more effective than conventional therapy (Fig. 4). The results of the pairwise and network meta-analyses are presented in Table 3. The ranking of effectiveness probabilities indicated that MMAT was the most effective approach, followed by CIAT, HISLT, CSLT, and conventional therapy (Fig. 5).

**Table 3:** Network meta-analysis results for quality of life of patients in the chronic phase.

Pairwise meta-analysis				
**MMAT**	0.41 [0.10; 0.71]			0.77 [0.43; 1.11]
0.38 [0.09; 0.68]	**CIAT**			0.38 [0.08; 0.67]
0.57 [0.12; 1.02]	0.19 [−0.24; 0.61]	**HISLT**		0.21 [−0.11; 0.52]
0.73 [0.29; 1.17]	0.35 [−0.07; 0.76]	0.16 [−0.28; 0.60]	**CSLT**	0.05 [−0.25; 0.35]
0.78 [0.46; 1.10]	0.39 [0.10; 0.69]	0.21 [−0.11; 0.52]	0.05 [−0.25; 0.35]	**Conventional**
Network meta-analysis				
Data are expressed as SMD [95% CI]. Significant results are underscored. “-” means data not applicable.				

**Note:**

The table presents two types of estimates. Results in the upper-right triangle (above the gray diagonal) represent the SMD from direct (pairwise) meta-analyses. Results in the lower-left triangle (below the gray diagonal) represent the SMD from the final network meta-analysis, which incorporates both direct and indirect evidence. Abbreviations: 95% CI, 95% credible interval; CIAT, constraint-induced aphasia therapy; CSLT, computerized speech and language therapy; HISLT, high-intensity speech and language therapy; MMAT, multimodality aphasia therapy; SMD, standard mean difference.

The network plot (Fig. S3) and heterogeneity analysis (Table S4) showed no significant inconsistency between direct and indirect comparisons. The forest plots comparing pairwise and network meta-analyses (Fig. S3) also revealed no significant differences.

### Results of language performance (fluency, comprehension, repetition, and naming)

We further analyzed specific language domains, including fluency, comprehension, repetition, and naming, for both the overall population and the chronic phase subgroup. The network diagrams for these outcomes are presented in the remaining Supplemental Figures. In terms of effectiveness compared to no intervention, the network meta-analysis revealed no statistically significant differences for any specific speech therapy across these four domains. Similarly, in the chronic phase subgroup, no intervention demonstrated significant superiority over the control group.

Although no statistically significant SMDs were found, probability rankings offered insights into potential trends. For fluency, MIT ranked highest in the overall population, whereas CIAT and MIT showed comparable high probabilities of effectiveness in the chronic phase. For comprehension, BOX therapy and MIT showed higher probabilities of effectiveness. For repetition, MIT consistently ranked highest. For naming, CSLT ranked highest in the chronic phase. Detailed results for pairwise and network meta-analyses for these outcomes are provided in the Supplemental Materials.

### Certainty of evidence

We evaluated the certainty of evidence using CINeMA (Nikolakopoulou et al., 2020). The confidence ratings were predominantly low to very low across comparisons. The primary reason for downgrading was imprecision, as many studies were classified as having ‘some concerns’ or ‘major concerns’ due to the limited number of included studies. Additionally, concerns about within-study bias and heterogeneity were noted, largely attributed to challenges in blinding participants and variations between studies. The detailed results of the CINeMA assessment are provided in the Supplemental Tables.

## Discussion

Poststroke aphasia significantly hinders activities of daily living and diminishes quality of life (Berthier, 2005; Wortman-Jutt & Edwards, 2019). Our network meta-analysis aimed to dissect the impacts of various speech therapies on both patients’ quality of life and specific language domains.

A key finding of our study is the dissociation between functional quality of life and isolated language performance. Our analysis revealed that both MMAT and CIAT yielded significant improvements in quality of life relative to conventional therapy. This is clinically profound. While our analysis of specific language domains (fluency, comprehension, repetition, and naming) did not identify any single therapy as statistically superior to no intervention, the significant gain in quality of life suggests that MMAT and CIAT may confer benefits that extend beyond mere linguistic accuracy. Patients with poststroke aphasia often resort to compensatory strategies, which can lead to learned nonuse (Ciccone et al., 2016; Sickert et al., 2014). CIAT combats this by forcing verbal practice through constraint (Szaflarski et al., 2015; Ciccone et al., 2016; Sickert et al., 2014; Wilssens et al., 2015; Woldag et al., 2017), while MMAT engages patients through intensive multimodal cues (Pierce et al., 2023). The superior quality of life outcomes for these two approaches suggest that their high intensity and focus on communicative engagement may boost patients’ confidence and social participation—effects effectively captured by quality of life measures but potentially missed by specific linguistic subtests.

Although specific therapies did not show statistical superiority in isolated language domains in this meta-analysis, they hold distinct values in real-world clinical practice. For instance, CSLT may offer a standardized solution for increasing practice dosage without demanding additional therapist hours (Palmer et al., 2019; Spaccavento et al., 2021). MIT, while not ranking highest in overall fluency in our statistical model, may remain a theoretically sound pathway for engaging the right hemisphere (Van Der Meulen et al., 2016; Zhang et al., 2021). Similarly, PACE theoretically focuses on pragmatic exchange using any available modality, offering a vital bridge for patients with severe deficits (Raglio et al., 2016), whereas BOX therapy provides a cognitive neuropsychological approach for restoring specific linguistic processes (Wilssens et al., 2015).

It is important to conceptually acknowledge that the therapies compared in this analysis are not always interchangeable. The lack of statistical differentiation in our specific domain analysis likely reflects this heterogeneity. Approaches like MIT are designed for specific profiles, such as non-fluent aphasia (Van Der Meulen et al., 2016; Zhang et al., 2021), while CIAT may be less suitable for patients with very mild deficits or predominant writing impairments (Wilssens et al., 2015). Our network meta-analysis provides a valuable macroscopic view of the average treatment effects across studies. However, consistent with the insight into real-world application, we stress that the choice of therapy in clinical practice should be a nuanced decision, guided by a patient’s individual profile, including aphasia type and severity, lesion characteristics, personal goals, and the specific stage of recovery.

This study has several strengths. First, this is a network meta-analysis of RCTs focused on dissecting the impacts of different speech therapies on both quality of life and specific language domains in patients with poststroke aphasia. Second, to identify relevant RCTs, we adopted broad inclusion criteria, considered major databases, and imposed no language restrictions. Third, according to PEDro scores, the quality of the included RCTs was good overall and excellent in one case. Fourth, no significant inconsistencies were observed between the results of direct and indirect comparisons, indicating favorable coherence.

This study also has several limitations. First, considerable interstudy variations, namely in the stages of poststroke aphasia, types of aphasia, and treatment duration, may have affected the effectiveness of the interventions. Second, although the risk of bias in assessment was generally “good,” in most of the studies, the patients or therapists were not blind to the intervention because of the nature of the interventions. Third, regarding specific language domains, the number of studies contributing to each network node was relatively small, leading to wide confidence intervals and imprecision (as noted in our CINeMA assessment). This may have limited our statistical power to detect smaller differences between active therapies. Reflecting these limitations, it is important to explicitly acknowledge that the overall certainty of evidence assessed by CINeMA was predominantly rated as “low to very low” across most comparisons. Therefore, these findings should be interpreted with caution. Fourth, the protocols of specific training strategies differed between studies; thus, providing suggestions for the ideal training dosage or intensity is challenging. Future studies should stratify treatment effects by different types and phases of poststroke aphasia to reduce heterogeneity.

In summary, this network meta-analysis suggests that among the currently available RCT evidence, both CIAT and MMAT show significant promise for improving quality of life in patients with poststroke aphasia when compared to no intervention. However, no specific speech therapy demonstrated statistical superiority over no intervention across isolated language domains (fluency, comprehension, repetition, and naming). Given these findings and the study limitations, particularly the uneven distribution of evidence, these conclusions should be interpreted with a clinical lens. We do not recommend a single “protocol of choice,” based solely on statistical ranking, especially given that the P-score rankings were derived from estimates with wide, overlapping credible intervals. Rather, we suggest that while CIAT and MMAT are options for enhancing quality of life, the selection of therapy for specific linguistic deficits should be guided by individual patient profiles and goals. Future high-quality, large-scale RCTs are urgently needed to conduct direct head-to-head comparisons to further clarify the specific linguistic benefits of these promising therapies.

## Supplemental Information

10.7717/peerj.21118/supp-1 Supplemental Information 1 PRISMA 2020 checklist: Post-stroke aphasia.

10.7717/peerj.21118/supp-2 Supplemental Information 2 Linguistic performance.

10.7717/peerj.21118/supp-3 Supplemental Information 3 Supplementary Figures & Tables.

10.7717/peerj.21118/supp-4 Supplemental Information 4 Search terms.

10.7717/peerj.21118/supp-5 Supplemental Information 5 Intended audience.

## Additional Information and Declarations

### Data Availability

The following information was supplied regarding data availability:

The raw data are available in the Supplemental Files.
