# Supplementary material for: Speech therapy for poststroke aphasia: a network meta-analysis of randomized controlled trials
Source: PeerJ. 2026 Apr 15;14:e21118. doi: 10.7717/peerj.21118 (PMC13091577; doi:10.7717/peerj.21118)
Supplement: Supplemental Information 3 [file peerj-14-21118-s003.docx]

**Supplementary Figure Legends**

Supplementary Figure 1. Network plots of all studies showing the quality of life of all patients.

Supplementary Figure 2. Forest plots of pairwise and network meta-analyses showing the quality of life of all patients.

Supplementary Figure 3. Network plots of all studies showing the quality of life of patients in the chronic phase.

Supplementary Figure 4. Forest plots of pairwise and network meta-analyses showing the quality of life of patients in the chronic phase.

Supplementary Figure 5. Network plots of all studies showing the fluency of all patients.

Supplementary Figure 6. Forest plots of all results showing the fluency of all patients.

Supplementary Figure 7. Forest plots of pairwise and network meta-analyses showing the fluency of all patients.

Supplementary Figure 8. Distribution of probabilities of effectiveness for each speech therapy showing the fluency of all patients.

Supplementary Figure 9. Network plots of all studies showing the fluency of patients in the chronic phase.

Supplementary Figure 10. Forest plots of all results showing the fluency of patients in the chronic phase.

Supplementary Figure 11. Forest plots of pairwise and network meta-analyses showing the fluency of patients in the chronic phase.

Supplementary Figure 12. Distribution of probabilities of effectiveness for each speech therapy showing the fluency of patients in the chronic phase.

Supplementary Figure 13. Network plots of all studies showing the comprehension of all patients.

Supplementary Figure 14. Forest plots of all results showing the comprehension of all patients.

Supplementary Figure 15. Forest plots of pairwise and network meta-analyses showing the comprehension of all patients.

Supplementary Figure 16. Distribution of probabilities of effectiveness for each speech therapy showing the comprehension of all patients.

Supplementary Figure 17. Network plots of all studies showing the comprehension of patients in the chronic phase.

Supplementary Figure 18. Forest plots of all results showing the comprehension of patients in the chronic phase.

Supplementary Figure 19. Forest plots of pairwise and network meta-analyses showing the comprehension of patients in the chronic phase.

Supplementary Figure 20. Distribution of probabilities of effectiveness for each speech therapy showing the comprehension of patients in the chronic phase.

Supplementary Figure 21. Network plots of all studies showing the repeat performance of all patients.

Supplementary Figure 22. Forest plots of all results showing the repeat performance of all patients.

Supplementary Figure 23. Distribution of probabilities of effectiveness for each speech therapy showing the repeat performance of all patients.

Supplementary Figure 24. Network plots of all studies showing the repeat performance of patients in the chronic phase.

Supplementary Figure 25. Forest plots of all results showing the repeat performance of patients in the chronic phase.

Supplementary Figure 26. Distribution of probabilities of effectiveness for each speech therapy showing the repeat performance of patients in the chronic phase.

Supplementary Figure 27. Network plots of all studies showing the naming performance of all patients.

Supplementary Figure 28. Forest plots of all results showing the naming performance of all patients.

Supplementary Figure 29. Forest plots of pairwise and network meta-analyses showing the naming performance of all patients.

Supplementary Figure 30. Distribution of probabilities of effectiveness for each speech therapy showing the naming performance of all patients.

Supplementary Figure 31. Network plots of all studies showing the naming performance of patients in the chronic phase.

Supplementary Figure 32. Forest plots of all results showing the naming performance of patients in the chronic phase.

Supplementary Figure 33. Forest plots of pairwise and network meta-analyses showing the naming performance of patients in the chronic phase.

Supplementary Figure 34. Distribution of probabilities of effectiveness for each speech therapy showing the naming performance of patients in the chronic phase.


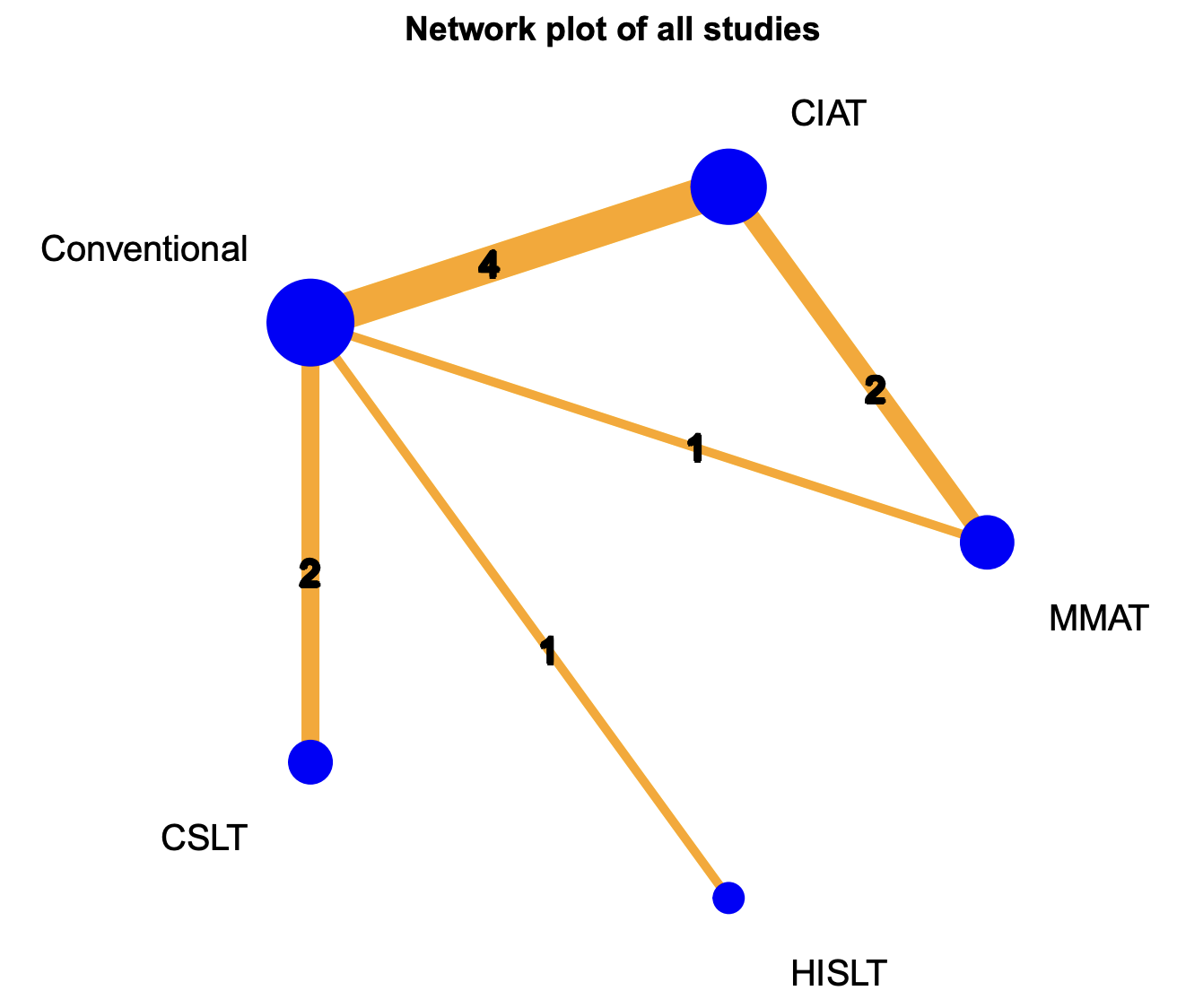


Supplementary Figure 1. Network plots of all studies showing the quality of life of all patients. Node size represents the total number of participants in each treatment arm, and edge width is proportional to the number of studies comparing the connected interventions. Abbreviations: CIAT, constraint-induced aphasia therapy; CSLT, computerized speech and language therapy; HISLT, high-intensity speech and language therapy; MMAT, multimodality aphasia therapy.


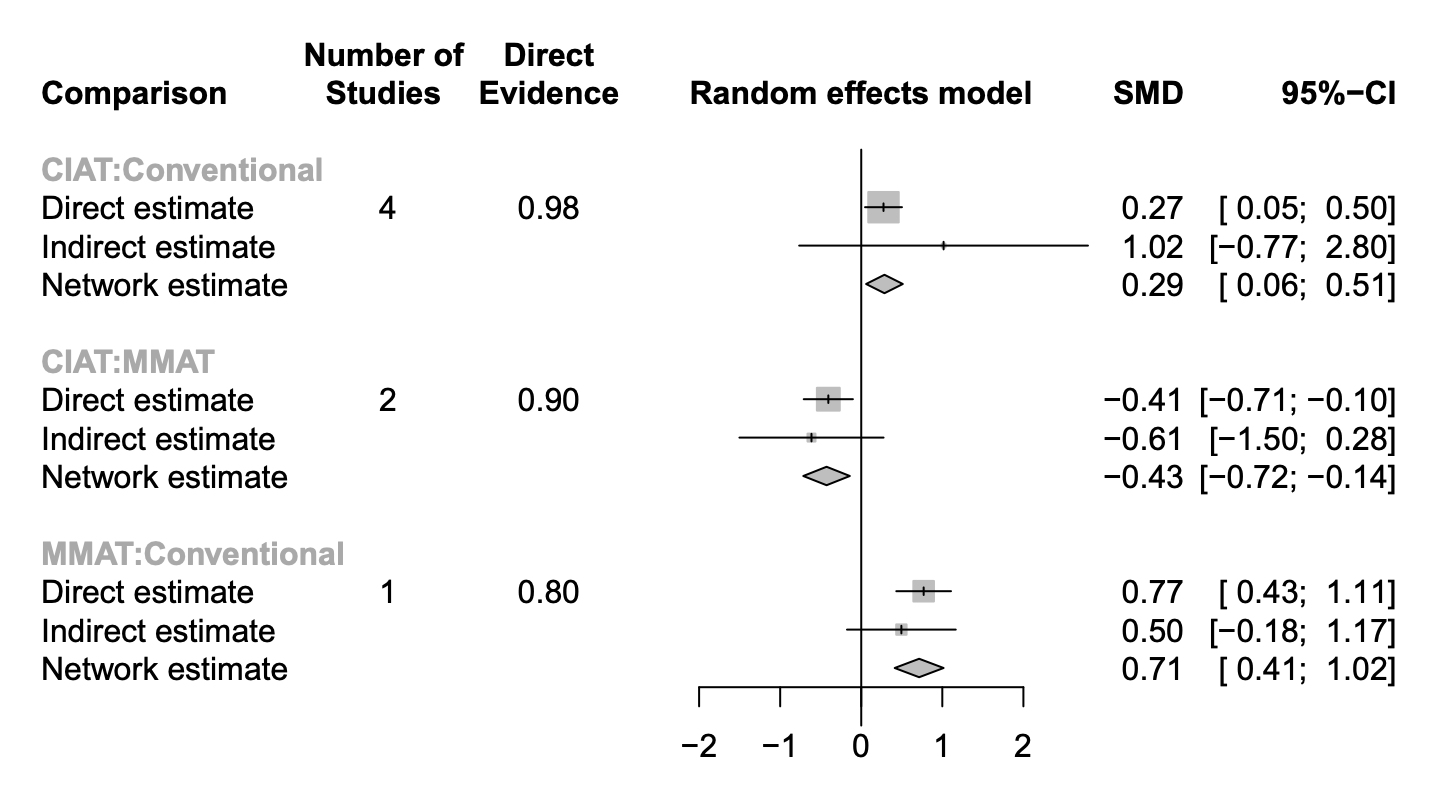


Supplementary Figure 2. Forest plots of pairwise and network meta-analyses showing the quality of life of all patients. Abbreviations: 95% CI, 95% credible interval; CIAT, constraint-induced aphasia therapy; MMAT, multimodality aphasia therapy; SMD, standard mean difference.


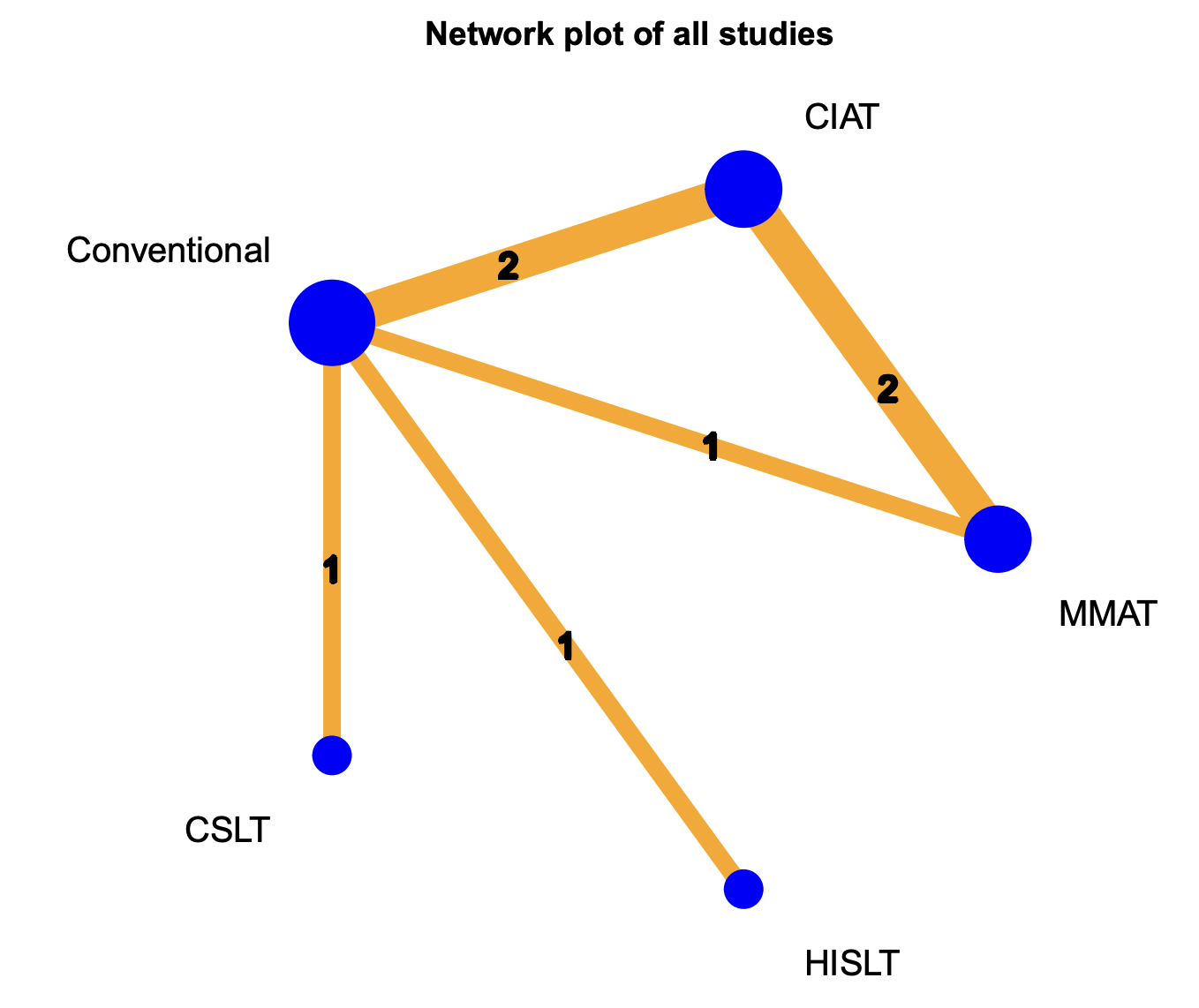


Supplementary Figure 3. Network plots of all studies showing the quality of life of patients in the chronic phase. Node size represents the total number of participants in each treatment arm, and edge width is proportional to the number of studies comparing the connected interventions. Abbreviations: CIAT, constraint-induced aphasia therapy; CSLT, computerized speech and language therapy; HISLT, high-intensity speech and language therapy; MMAT, multimodality aphasia therapy.


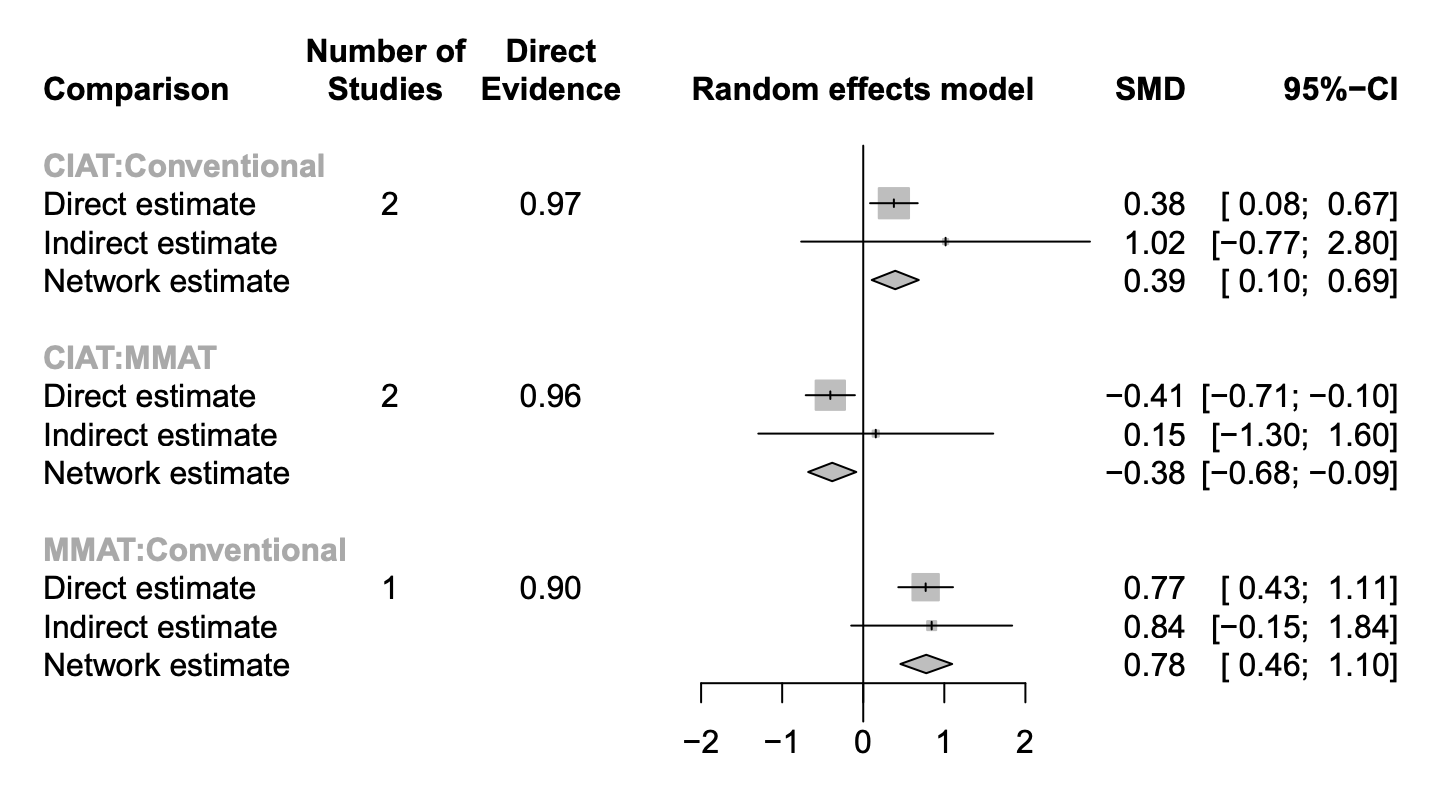


Supplementary Figure 4. Forest plots of pairwise and network meta-analyses showing the quality of life of patients in the chronic phase. Abbreviations: 95% CI, 95% credible interval; CIAT, constraint-induced aphasia therapy; MMAT, multimodality aphasia therapy; SMD, standard mean difference.


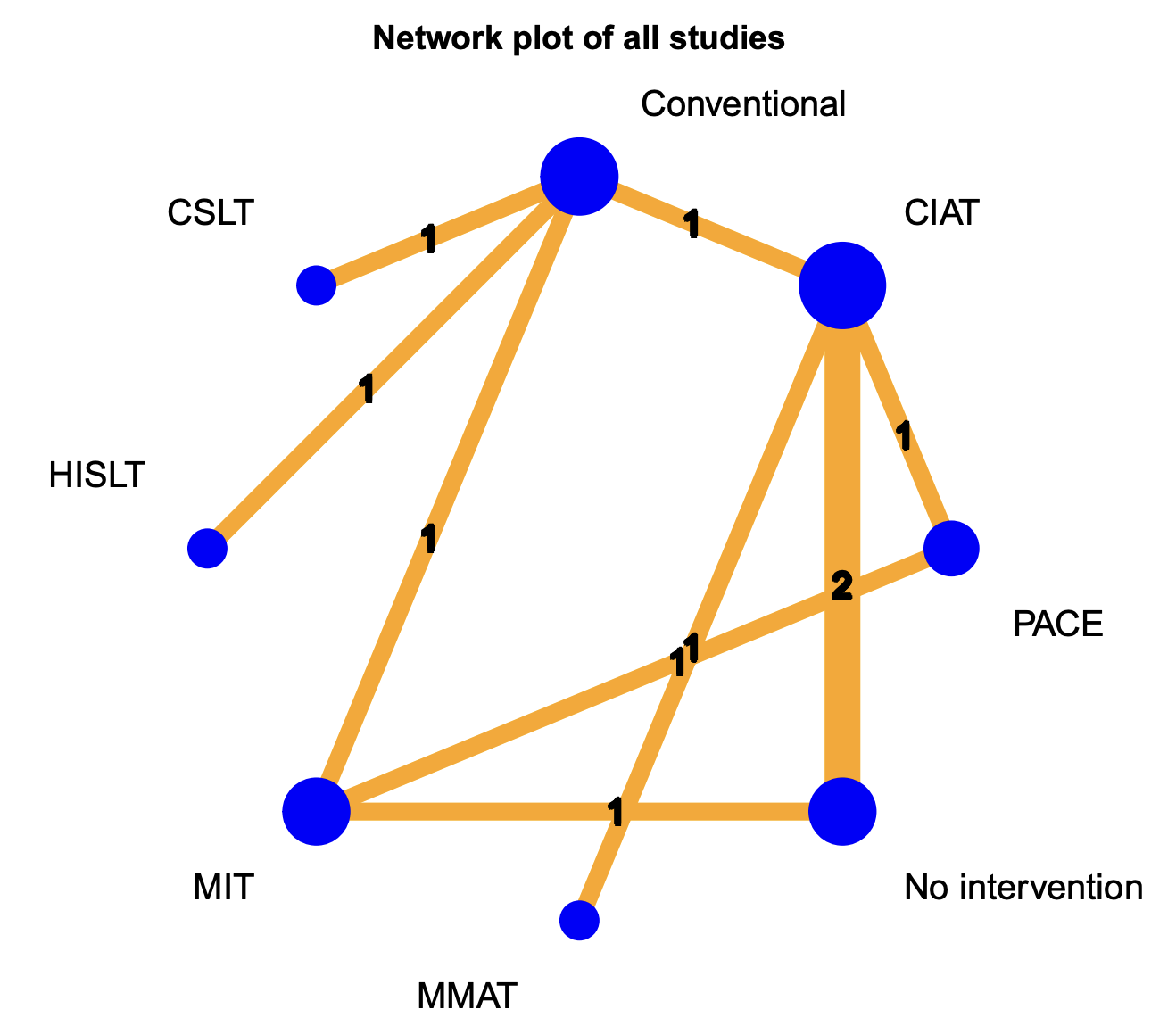


Supplementary Figure 5. Network plots of all studies showing the fluency of all patients. Node size represents the total number of participants in each treatment arm, and edge width is proportional to the number of studies comparing the connected interventions. Abbreviations: CIAT, constraint-induced aphasia therapy; CSLT, computerized speech and language therapy; HISLT, high-intensity speech and language therapy; MIT, melodic intonation therapy; MMAT, multimodality aphasia therapy; PACE, promoting aphasics’ communicative effectiveness.


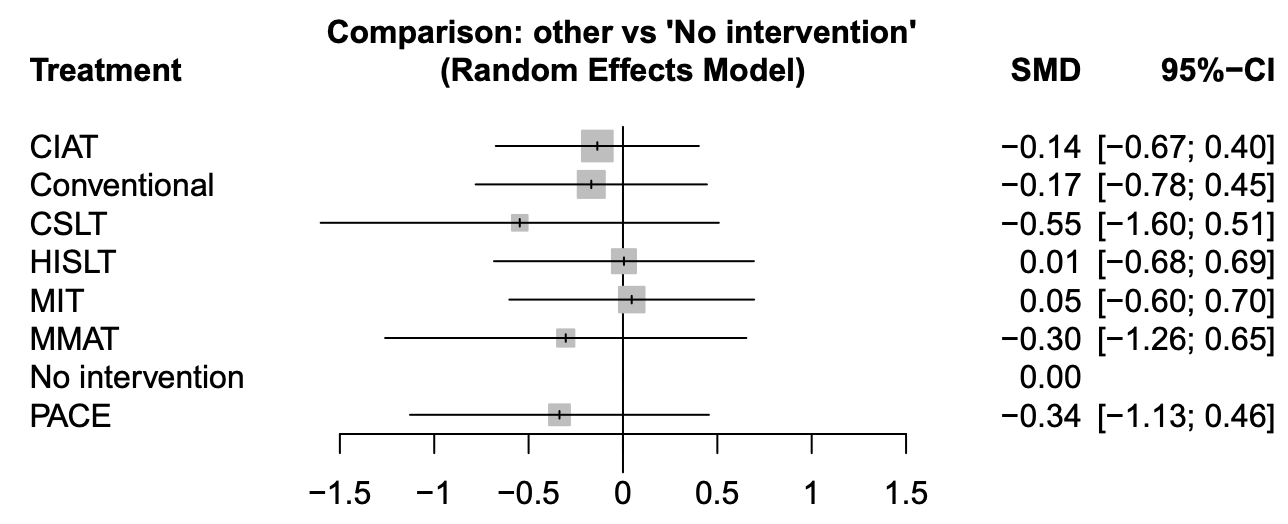


Supplementary Figure 6. Forest plots of all results showing the fluency of all patients. Abbreviations: 95% CI, 95% credible interval; CIAT, constraint-induced aphasia therapy; CSLT, computerized speech and language therapy; HISLT, high-intensity speech and language therapy; MIT, melodic intonation therapy; MMAT, multimodality aphasia therapy; PACE, promoting aphasics’ communicative effectiveness; SMD, standard mean difference.


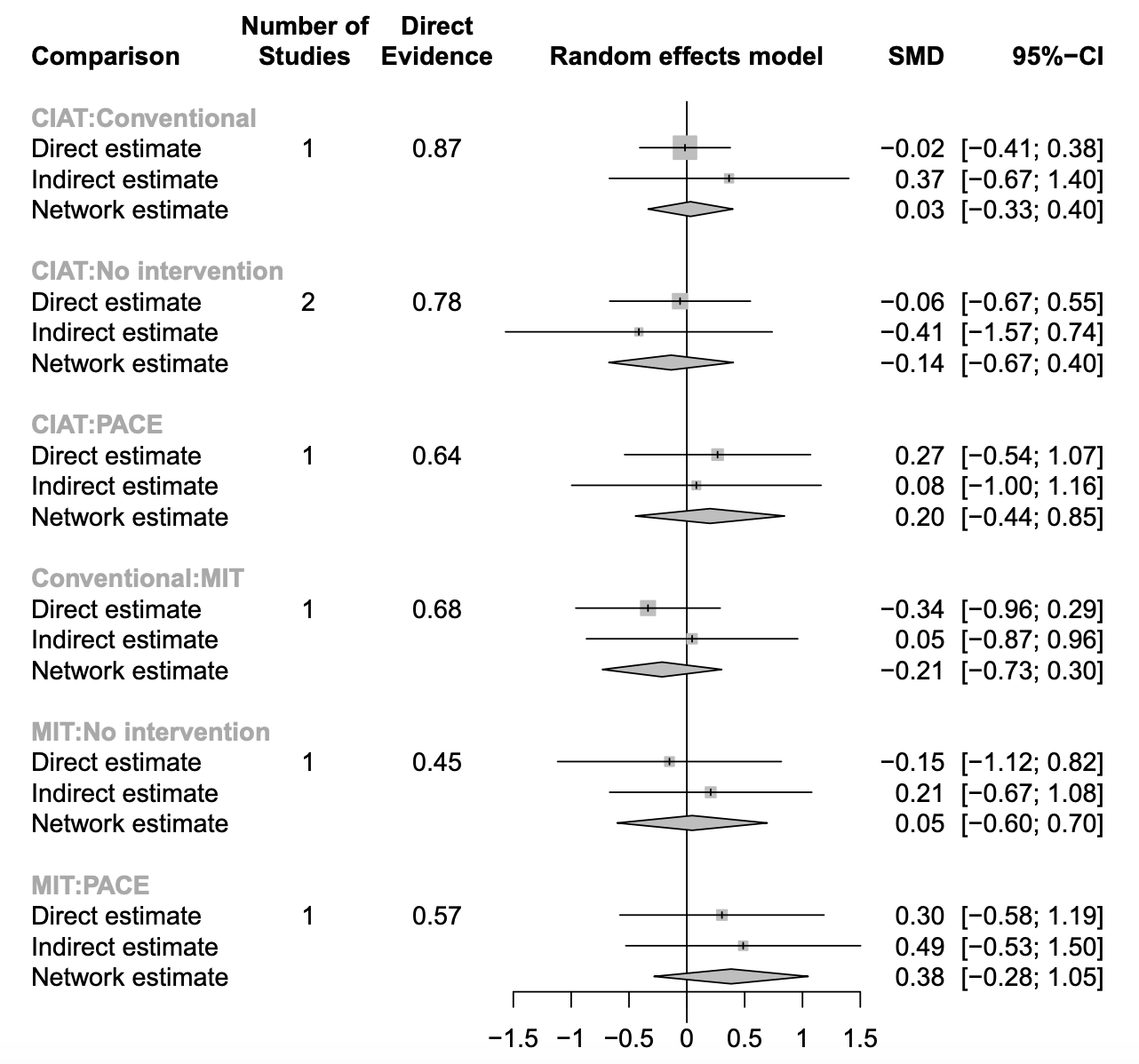


Supplementary Figure 7. Forest plots of pairwise and network meta-analyses showing the fluency of all patients. Abbreviations: 95% CI, 95% credible interval; CIAT, constraint-induced aphasia therapy; MIT, melodic intonation therapy; PACE, promoting aphasics’ communicative effectiveness; SMD, standard mean difference.


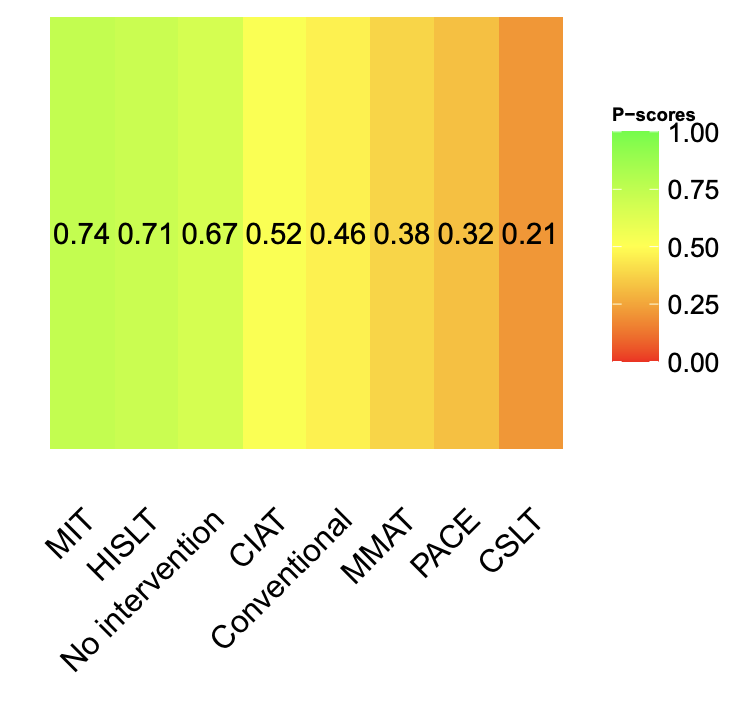


Supplementary Figure 8. Distribution of probabilities of effectiveness for each speech therapy showing the fluency of all patients. Abbreviations: CIAT, constraint-induced aphasia therapy; CSLT, computerized speech and language therapy; HISLT, high-intensity speech and language therapy; MIT, melodic intonation therapy; MMAT, multimodality aphasia therapy; PACE, promoting aphasics’ communicative effectiveness.


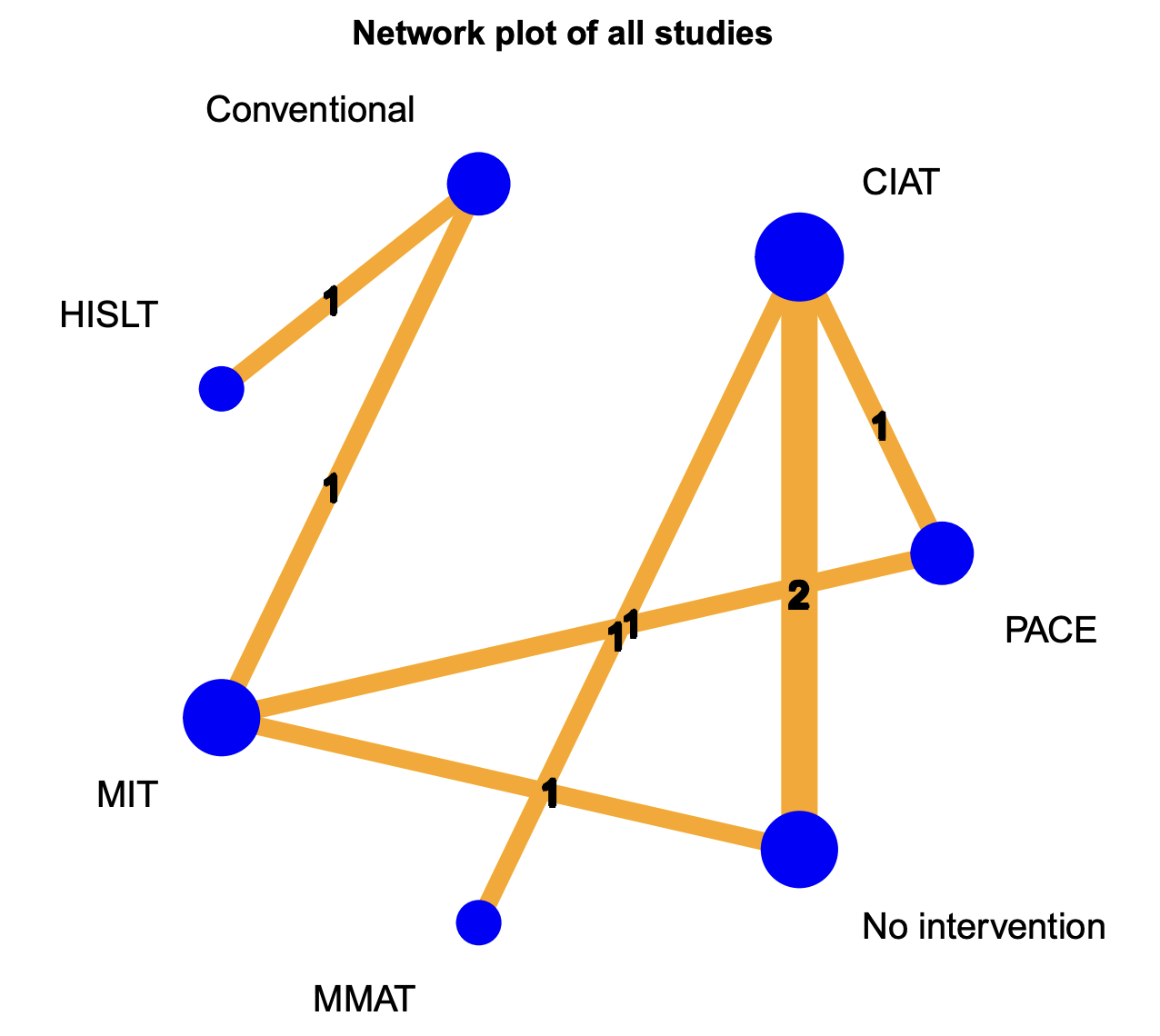


Supplementary Figure 9. Network plots of all studies showing the fluency of patients in the chronic phase. Node size represents the total number of participants in each treatment arm, and edge width is proportional to the number of studies comparing the connected interventions. Abbreviations: CIAT, constraint-induced aphasia therapy; HISLT, high-intensity speech and language therapy; MIT, melodic intonation therapy; MMAT, multimodality aphasia therapy; PACE, promoting aphasics’ communicative effectiveness.


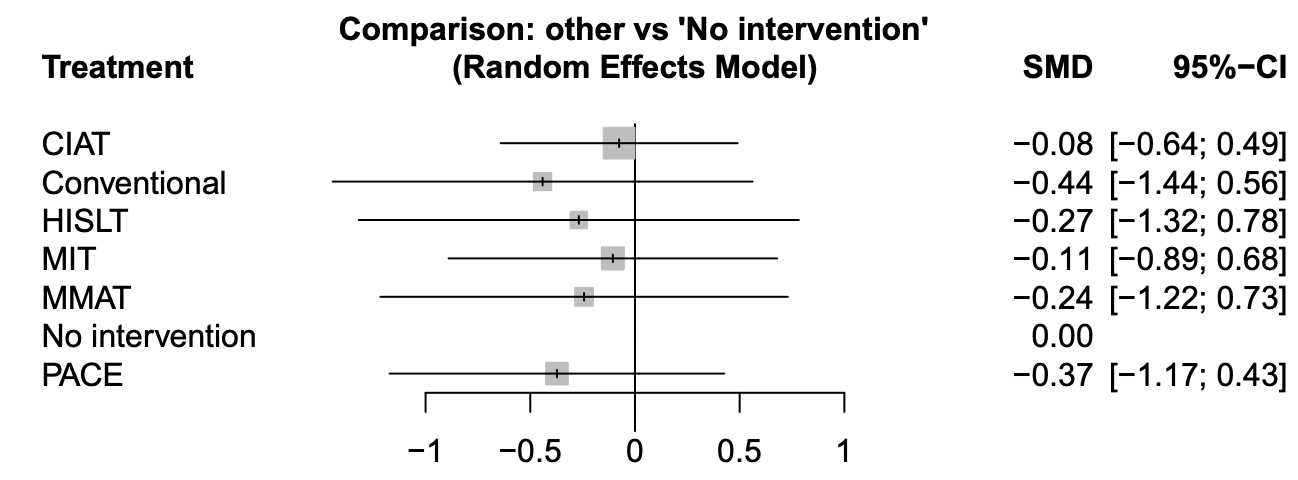


Supplementary Figure 10. Forest plots of all results showing the fluency of patients in the chronic phase. Abbreviations: 95% CI, 95% credible interval; CIAT, constraint-induced aphasia therapy; HISLT, high-intensity speech and language therapy; MIT, melodic intonation therapy; MMAT, multimodality aphasia therapy; PACE, promoting aphasics’ communicative effectiveness; SMD, standard mean difference.


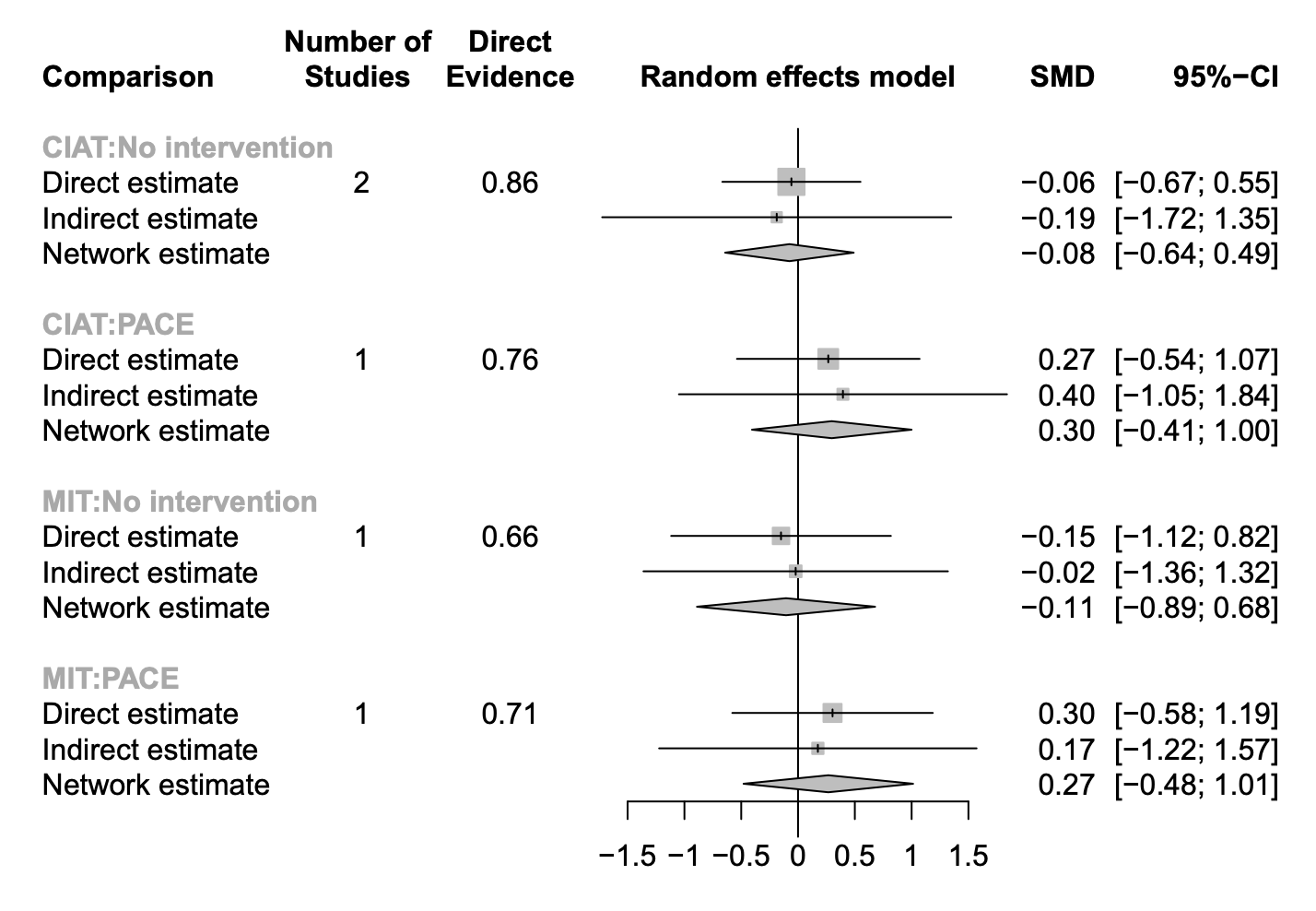


Supplementary Figure 11. Forest plots of pairwise and network meta-analyses showing the fluency of patients in the chronic phase. Abbreviations: 95% CI, 95% credible interval; CIAT, constraint-induced aphasia therapy; MIT, melodic intonation therapy; PACE, promoting aphasics’ communicative effectiveness; SMD, standard mean difference.


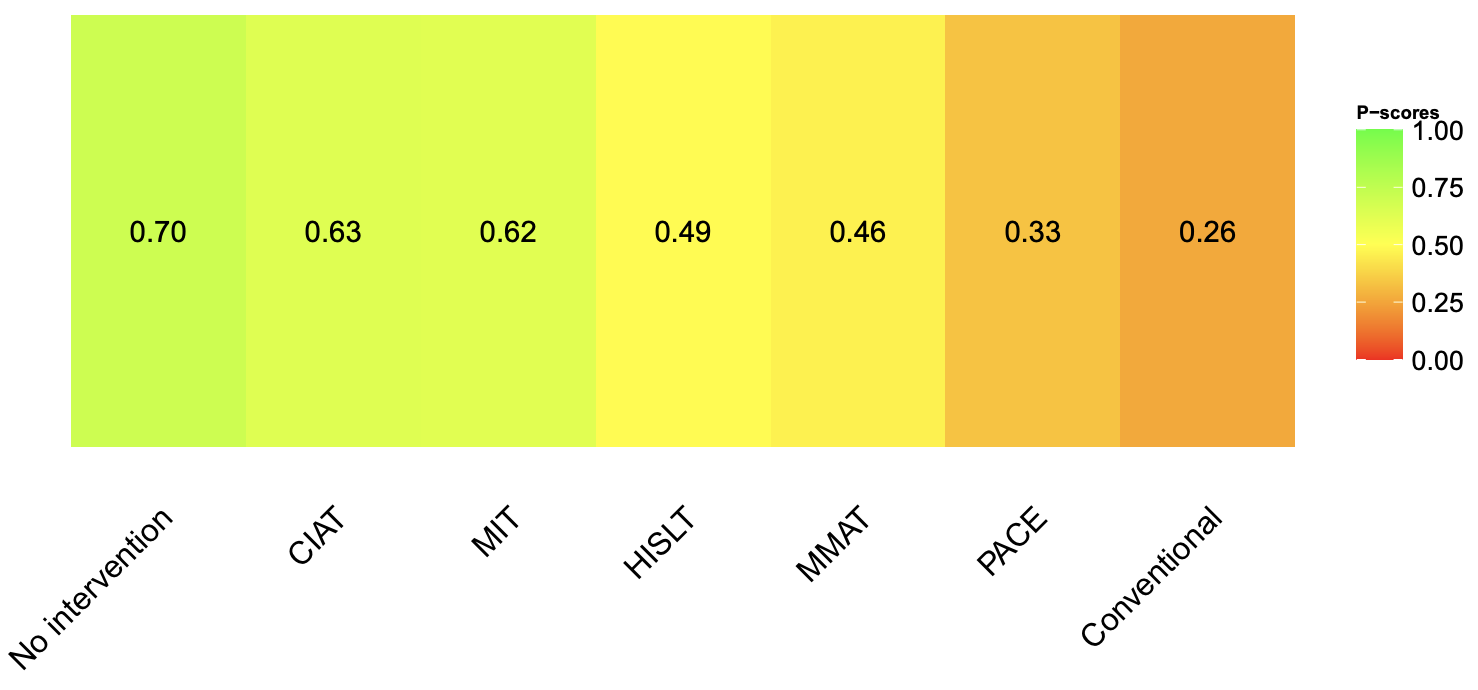


Supplementary Figure 12. Distribution of probabilities of effectiveness for each speech therapy showing the fluency of patients in the chronic phase. Abbreviations: CIAT, constraint-induced aphasia therapy; HISLT, high-intensity speech and language therapy; MIT, melodic intonation therapy; MMAT, multimodality aphasia therapy; PACE, promoting aphasics’ communicative effectiveness.


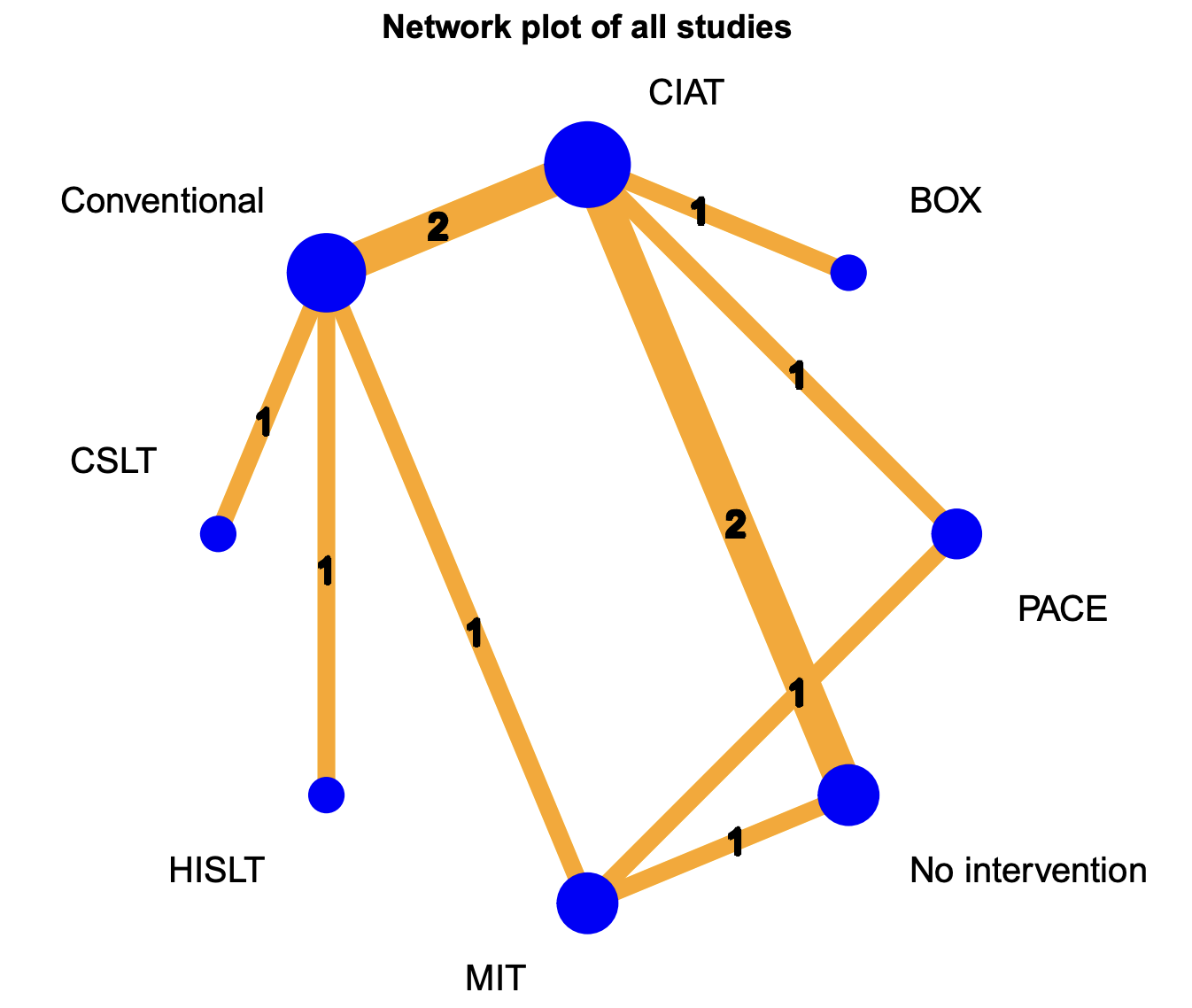


Supplementary Figure 13. Network plots of all studies showing the comprehension of all patients. Node size represents the total number of participants in each treatment arm, and edge width is proportional to the number of studies comparing the connected interventions. Abbreviations: CIAT, constraint-induced aphasia therapy; CSLT, computerized speech and language therapy; HISLT, high-intensity speech and language therapy; MIT, melodic intonation therapy; PACE, promoting aphasics’ communicative effectiveness.


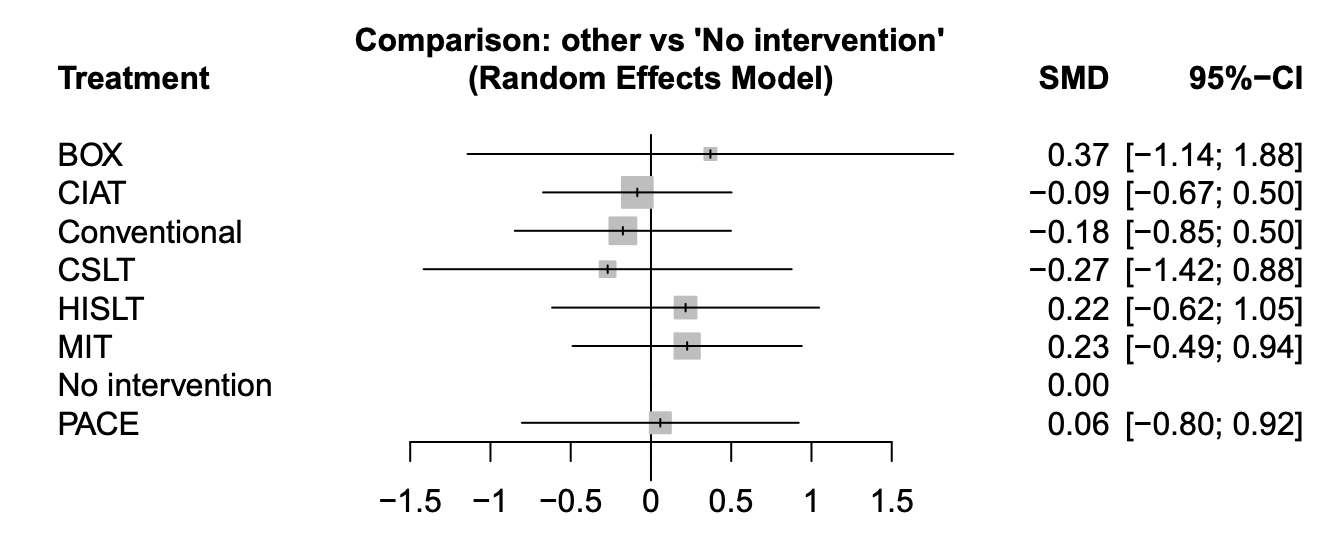


Supplementary Figure 14. Forest plots of all results showing the comprehension of all patients. Abbreviations: 95% CI, 95% credible interval; CIAT, constraint-induced aphasia therapy; CSLT, computerized speech and language therapy; HISLT, high-intensity speech and language therapy; MIT, melodic intonation therapy; PACE, promoting aphasics’ communicative effectiveness; SMD, standard mean difference.


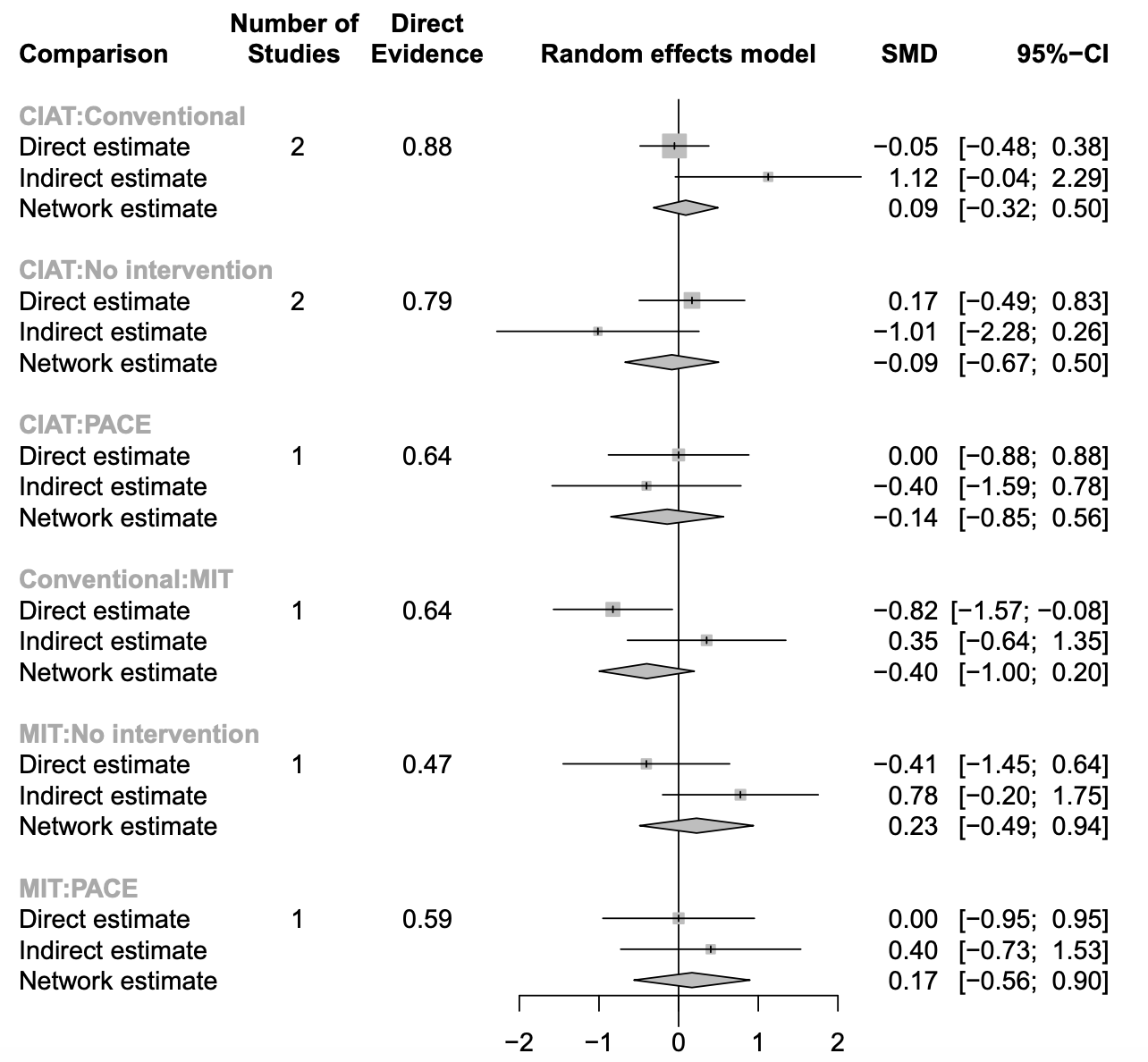


Supplementary Figure 15. Forest plots of pairwise and network meta-analyses showing the comprehension of all patients. Abbreviations: 95% CI, 95% credible interval; CIAT, constraint-induced aphasia therapy; MIT, melodic intonation therapy; PACE, promoting aphasics’ communicative effectiveness; SMD, standard mean difference.


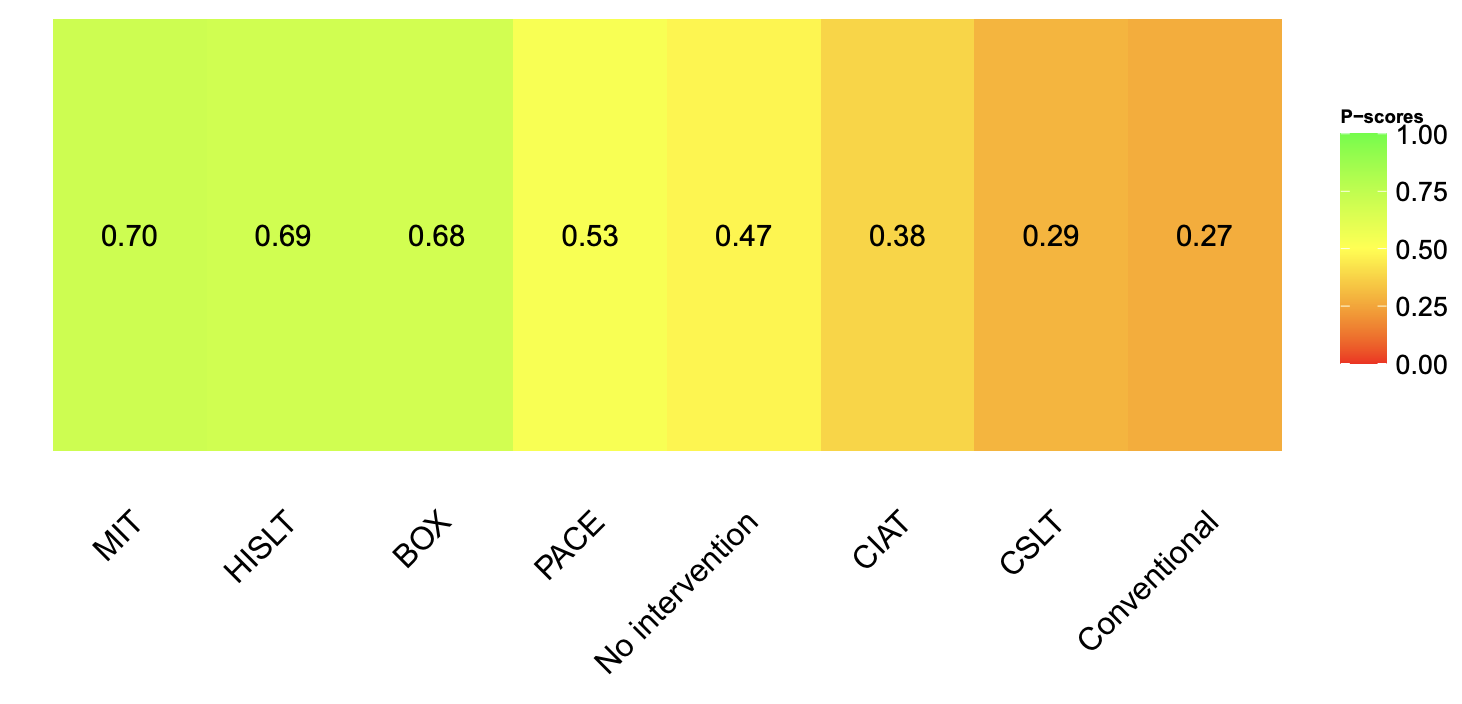


Supplementary Figure 16. Distribution of probabilities of effectiveness for each speech therapy showing the comprehension of all patients. Abbreviations: CIAT, constraint-induced aphasia therapy; CSLT, computerized speech and language therapy; HISLT, high-intensity speech and language therapy; MIT, melodic intonation therapy; PACE, promoting aphasics’ communicative effectiveness.


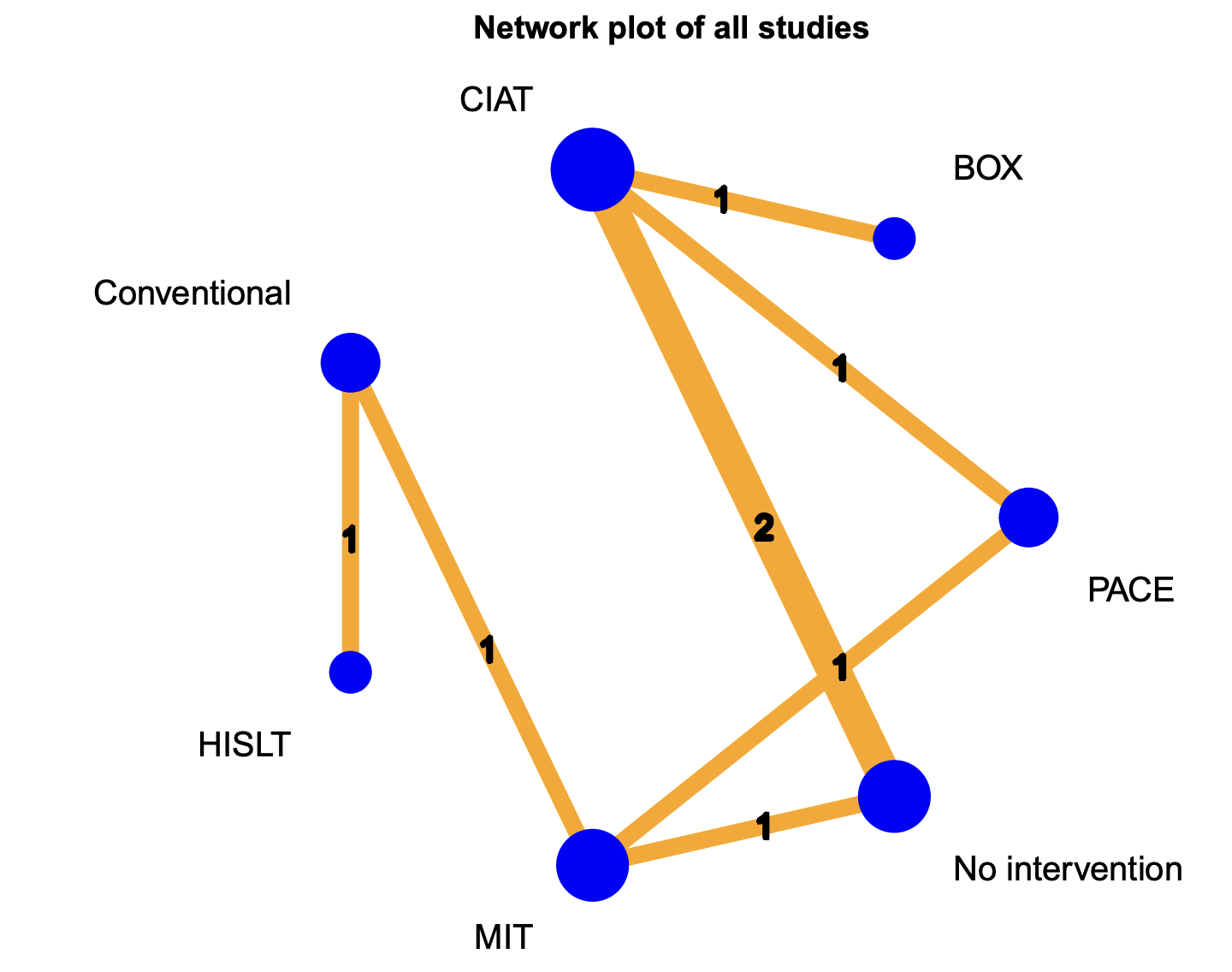


Supplementary Figure 17. Network plots of all studies showing the comprehension of patients in the chronic phase. Node size represents the total number of participants in each treatment arm, and edge width is proportional to the number of studies comparing the connected interventions. Abbreviations: CIAT, constraint-induced aphasia therapy; HISLT, high-intensity speech and language therapy; MIT, melodic intonation therapy; PACE, promoting aphasics’ communicative effectiveness.


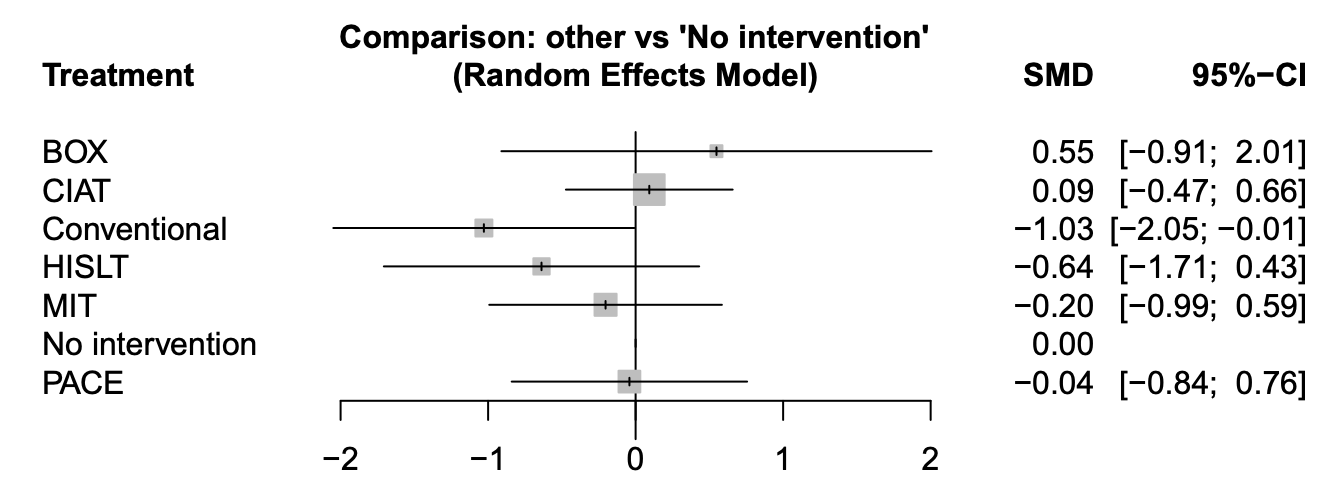


Supplementary Figure 18. Forest plots of all results showing the comprehension of patients in the chronic phase. Abbreviations: 95% CI, 95% credible interval; CIAT, constraint-induced aphasia therapy; HISLT, high-intensity speech and language therapy; MIT, melodic intonation therapy; PACE, promoting aphasics’ communicative effectiveness; SMD, standard mean difference.


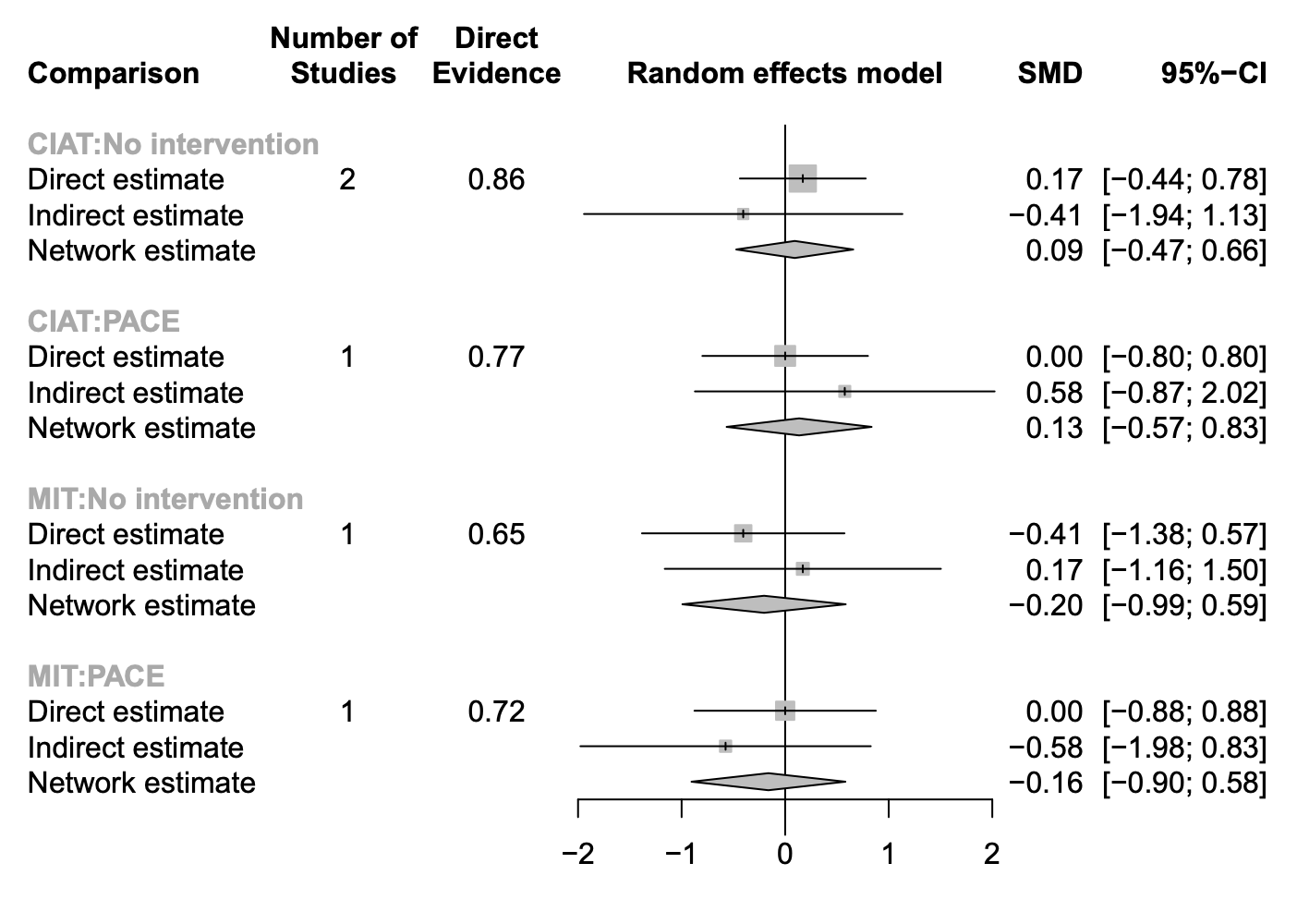


Supplementary Figure 19. Forest plots of pairwise and network meta-analyses showing the comprehension of patients in the chronic phase. Abbreviations: 95% CI, 95% credible interval; CIAT, constraint-induced aphasia therapy; MIT, melodic intonation therapy; PACE, promoting aphasics’ communicative effectiveness; SMD, standard mean difference.


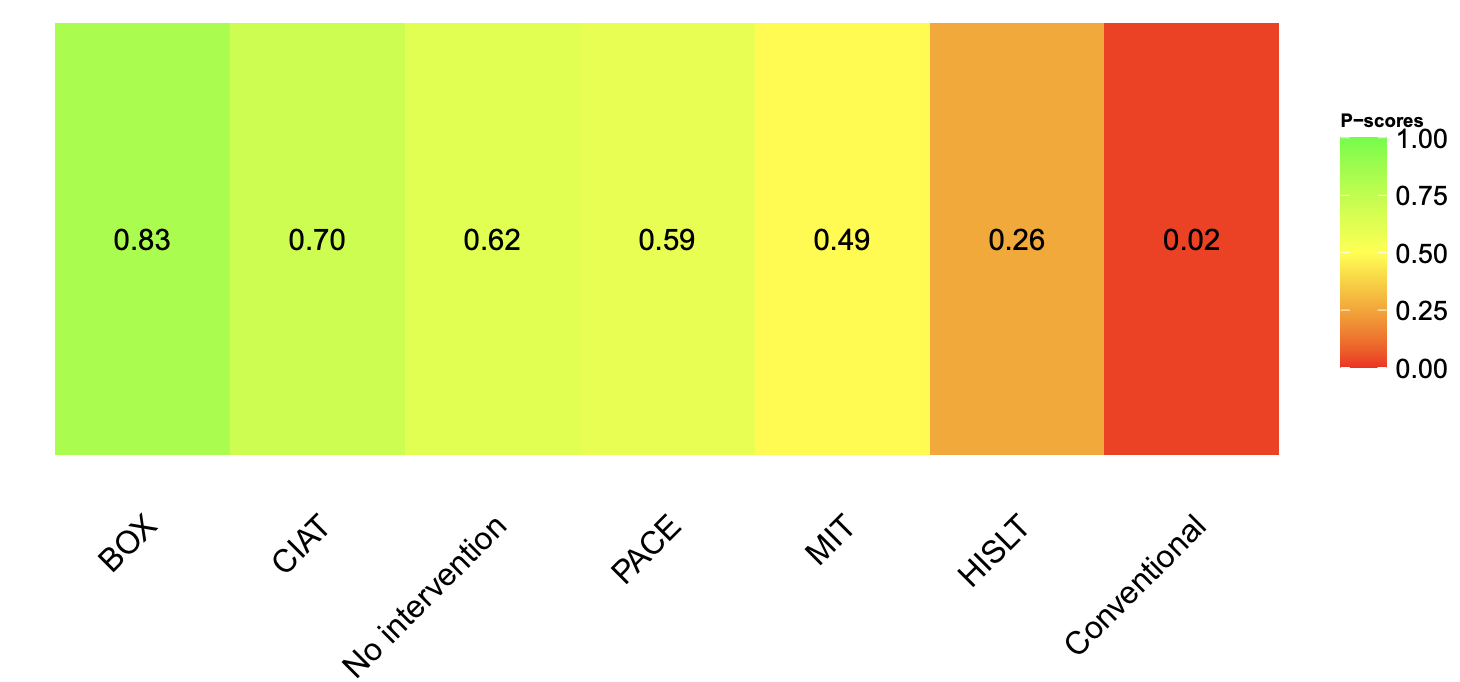


Supplementary Figure 20. Distribution of probabilities of effectiveness for each speech therapy showing the comprehension of patients in the chronic phase. Abbreviations: CIAT, constraint-induced aphasia therapy; HISLT, high-intensity speech and language therapy; MIT, melodic intonation therapy; PACE, promoting aphasics’ communicative effectiveness.


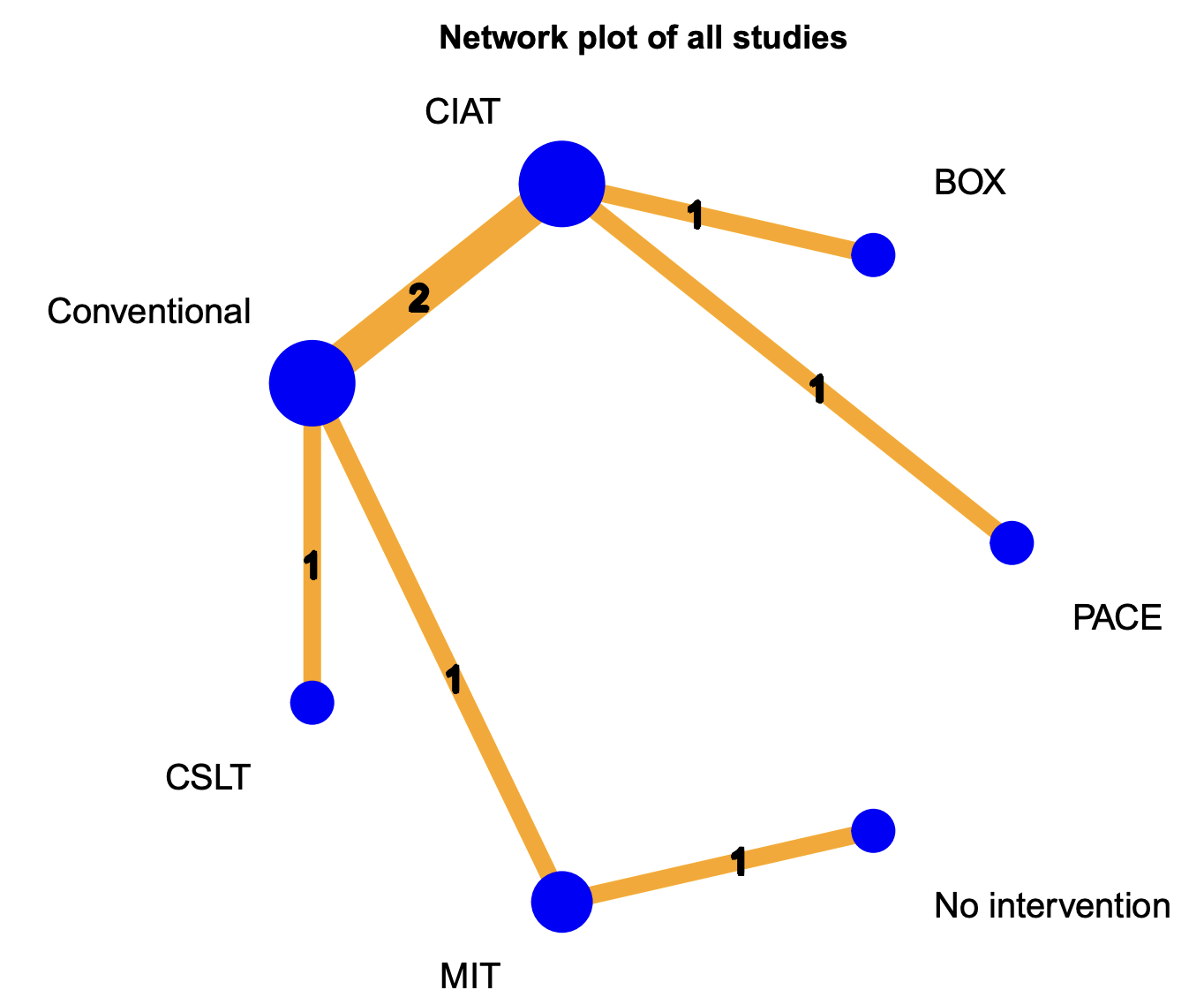


Supplementary Figure 21. Network plots of all studies showing the repeat performance of all patients. Node size represents the total number of participants in each treatment arm, and edge width is proportional to the number of studies comparing the connected interventions. Abbreviations: CIAT, constraint-induced aphasia therapy; CSLT, computerized speech and language therapy; MIT, melodic intonation therapy; PACE, promoting aphasics’ communicative effectiveness.


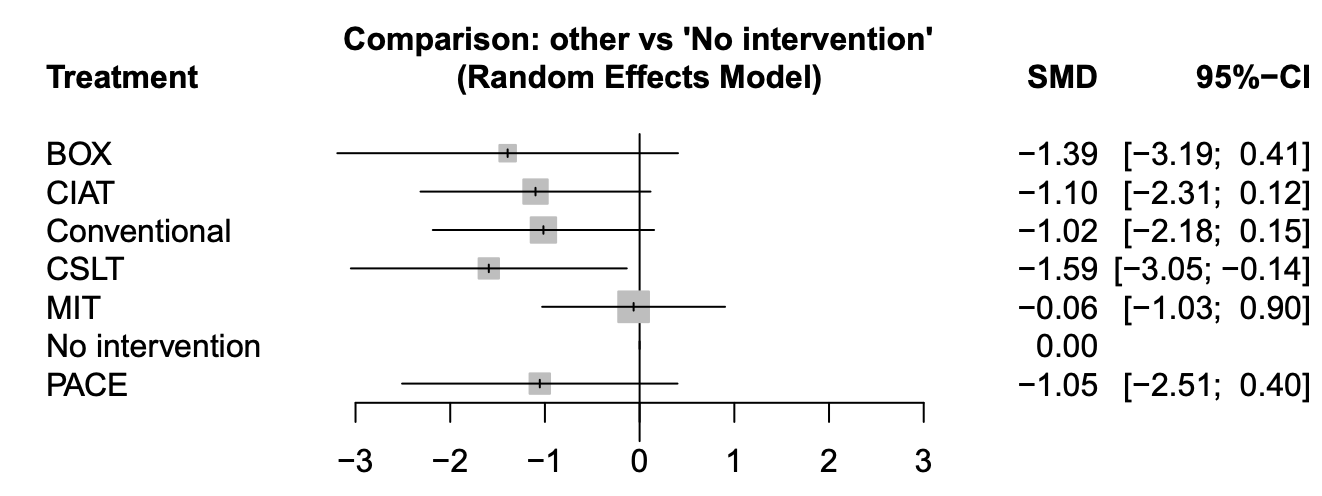


Supplementary Figure 22. Forest plots of all results showing the repeat performance of all patients. Abbreviations: 95% CI, 95% credible interval; CIAT, constraint-induced aphasia therapy; CSLT, computerized speech and language therapy; MIT, melodic intonation therapy; PACE, promoting aphasics’ communicative effectiveness; SMD, standard mean difference.


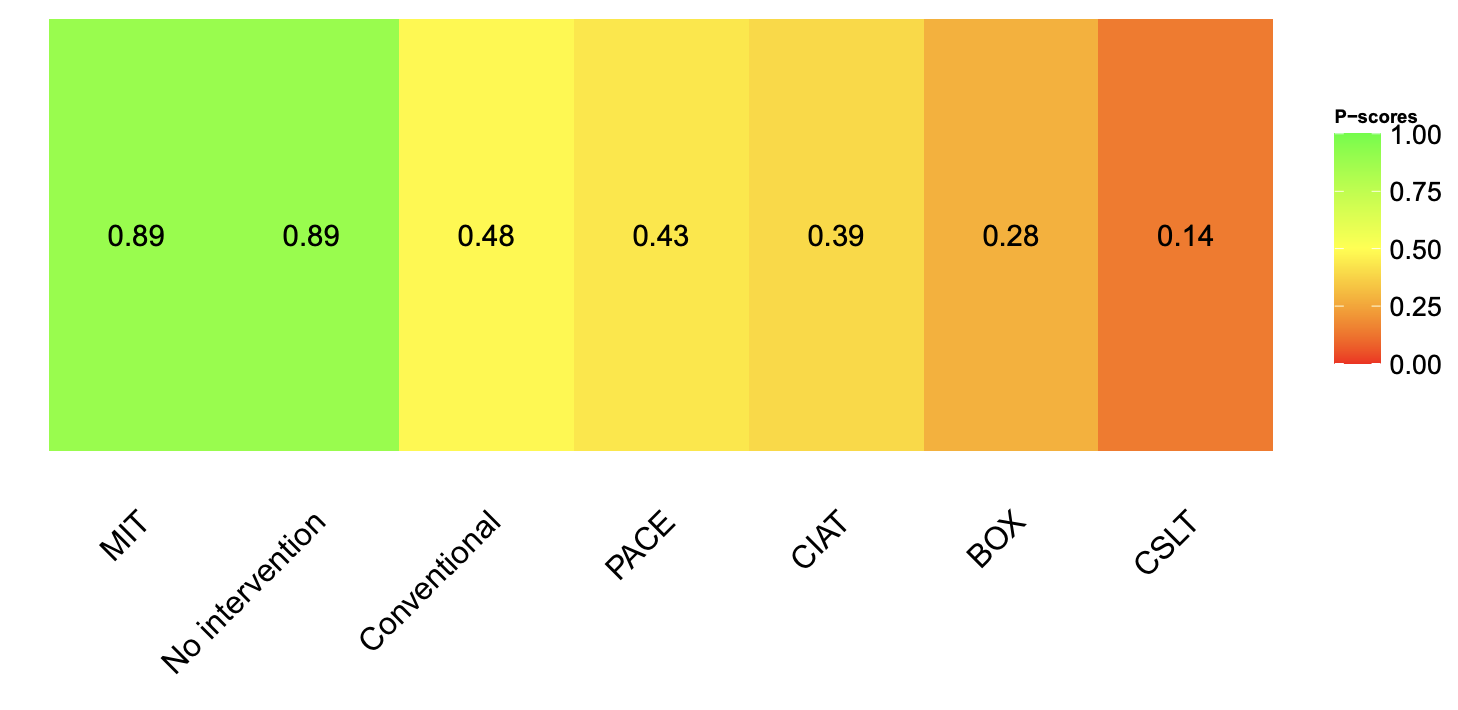


Supplementary Figure 23. Distribution of probabilities of effectiveness for each speech therapy showing the repeat performance of all patients. Abbreviations: CIAT, constraint-induced aphasia therapy; CSLT, computerized speech and language therapy; MIT, melodic intonation therapy; PACE, promoting aphasics’ communicative effectiveness.


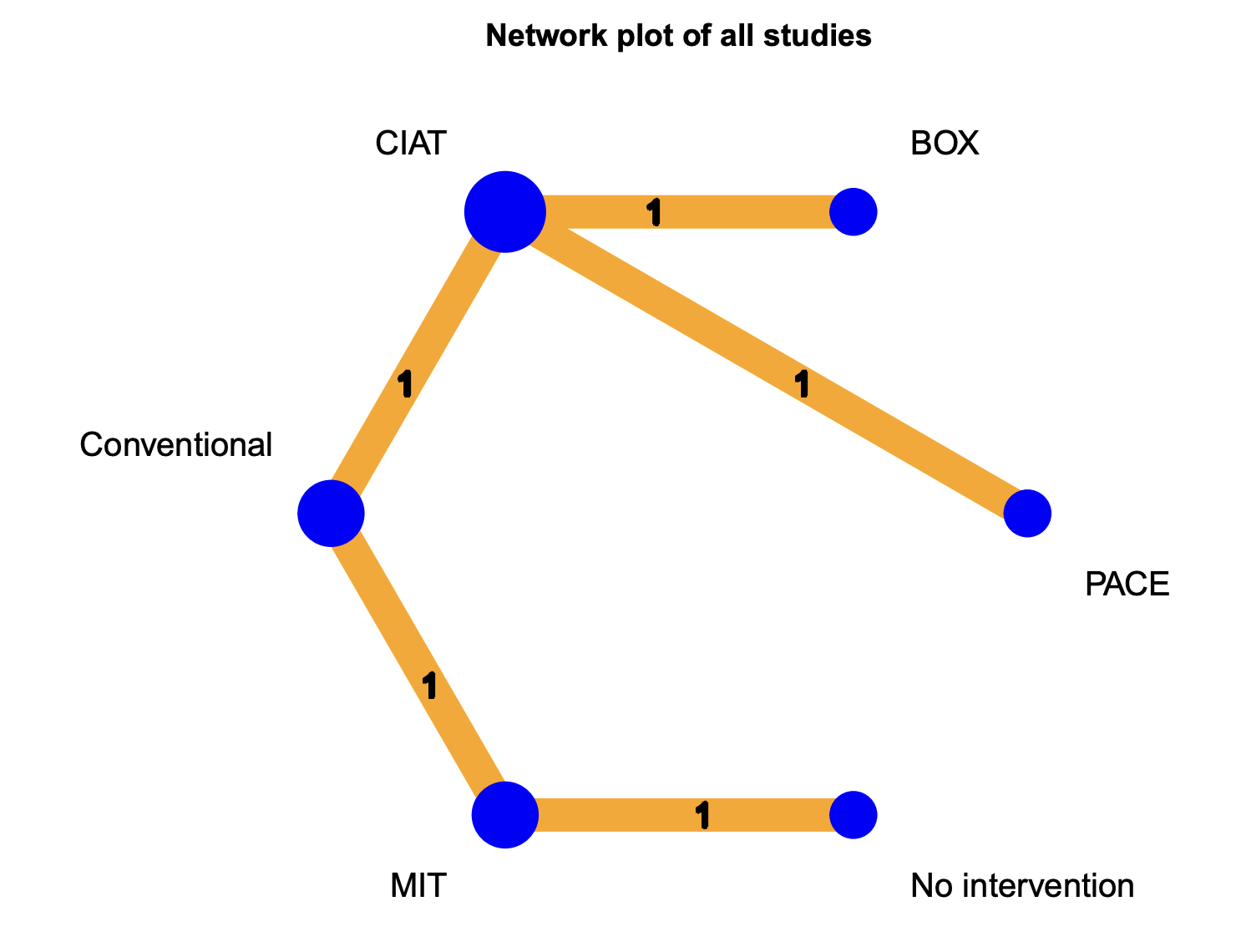


Supplementary Figure 24. Network plots of all studies showing the repeat performance of patients in the chronic phase. Node size represents the total number of participants in each treatment arm, and edge width is proportional to the number of studies comparing the connected interventions. Abbreviations: CIAT, constraint-induced aphasia therapy; MIT, melodic intonation therapy; PACE, promoting aphasics’ communicative effectiveness.


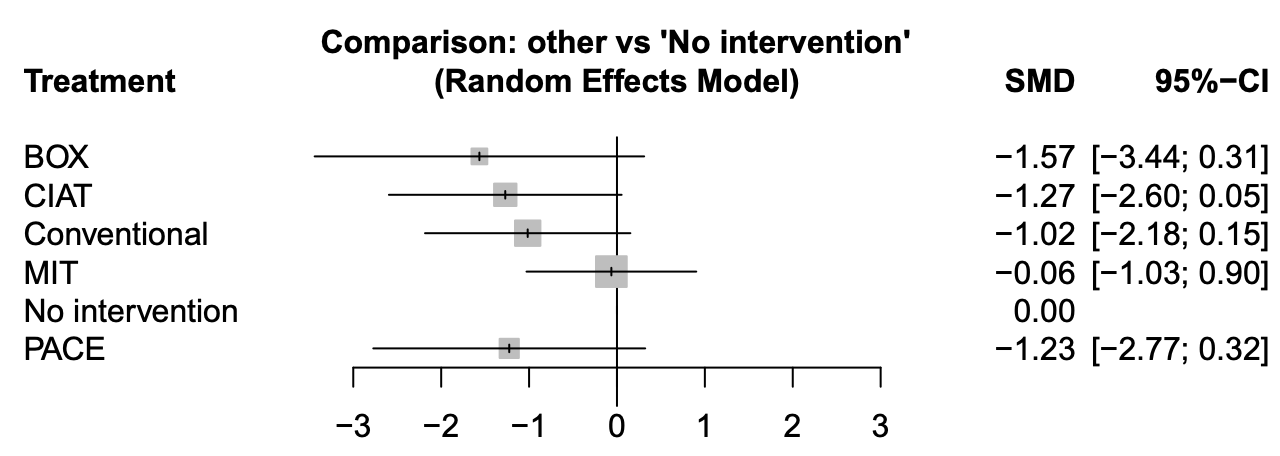


Supplementary Figure 25. Forest plots of all results showing the repeat performance of patients in the chronic phase. Abbreviations: 95% CI, 95% credible interval; CIAT, constraint-induced aphasia therapy; MIT, melodic intonation therapy; PACE, promoting aphasics’ communicative effectiveness; SMD, standard mean difference.


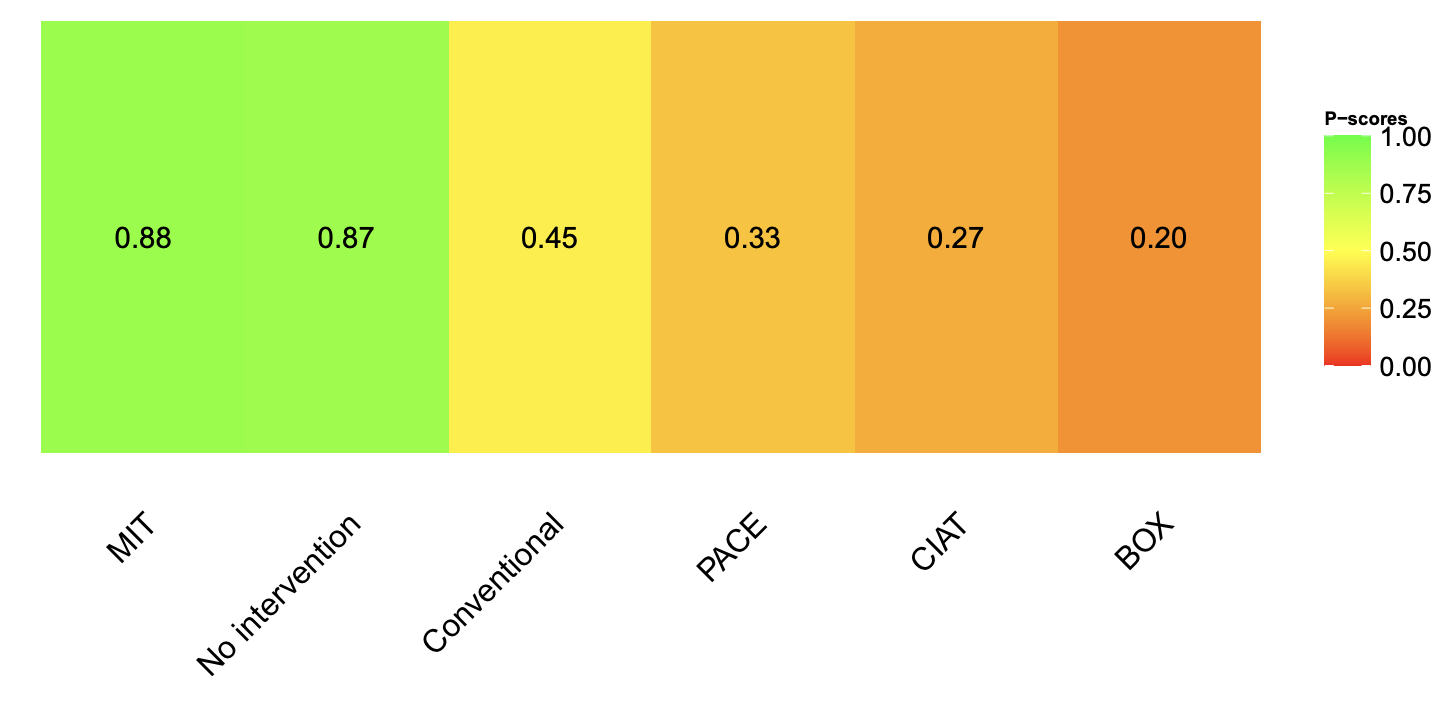


Supplementary Figure 26. Distribution of probabilities of effectiveness for each speech therapy showing the repeat performance of patients in the chronic phase. Abbreviations: CIAT, constraint-induced aphasia therapy; MIT, melodic intonation therapy; PACE, promoting aphasics’ communicative effectiveness.


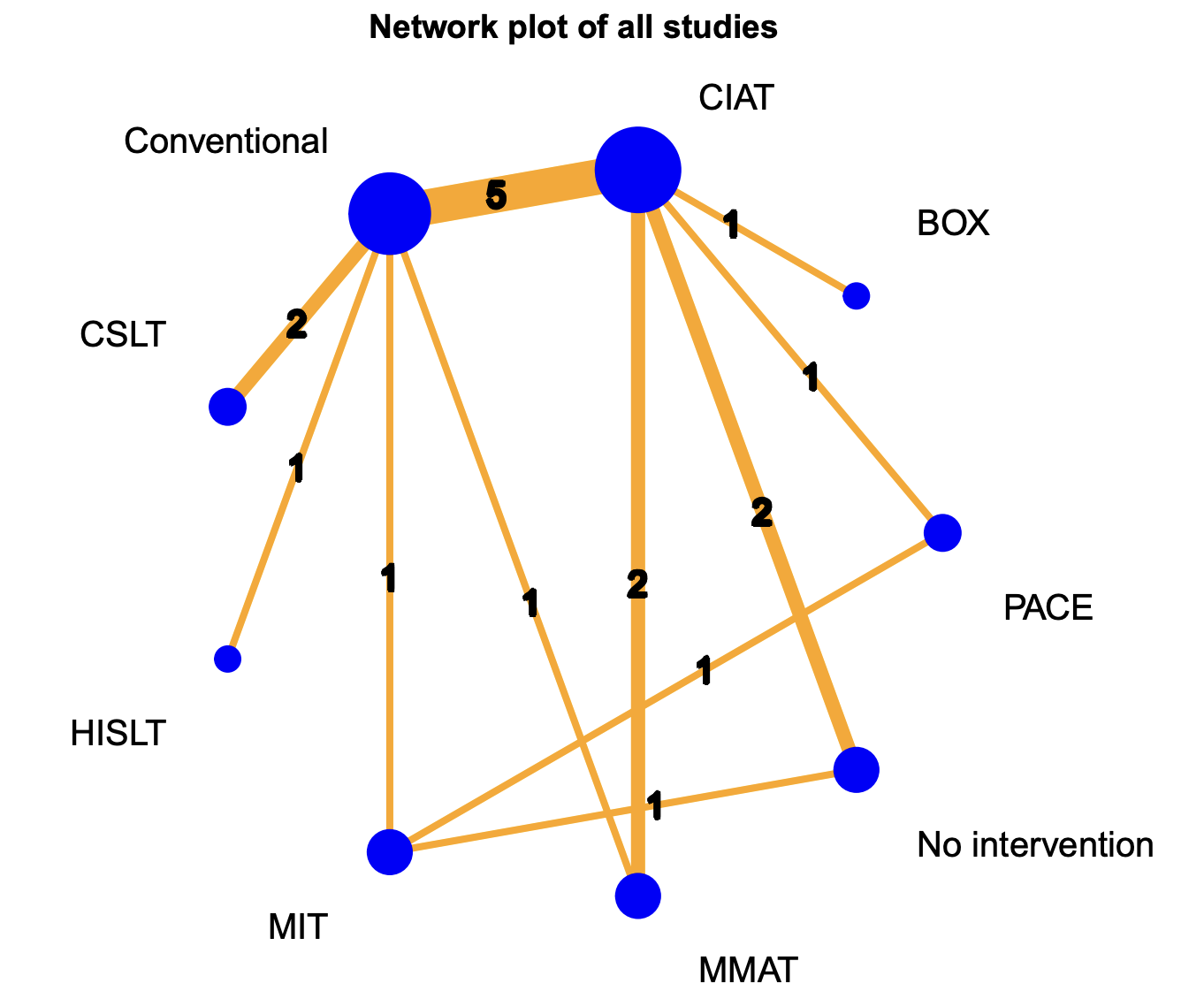


Supplementary Figure 27. Network plots of all studies showing the naming performance of all patients. Node size represents the total number of participants in each treatment arm, and edge width is proportional to the number of studies comparing the connected interventions. Abbreviations: CIAT, constraint-induced aphasia therapy; CSLT, computerized speech and language therapy; HISLT, high-intensity speech and language therapy; MIT, melodic intonation therapy; MMAT, multimodality aphasia therapy; PACE, promoting aphasics’ communicative effectiveness.


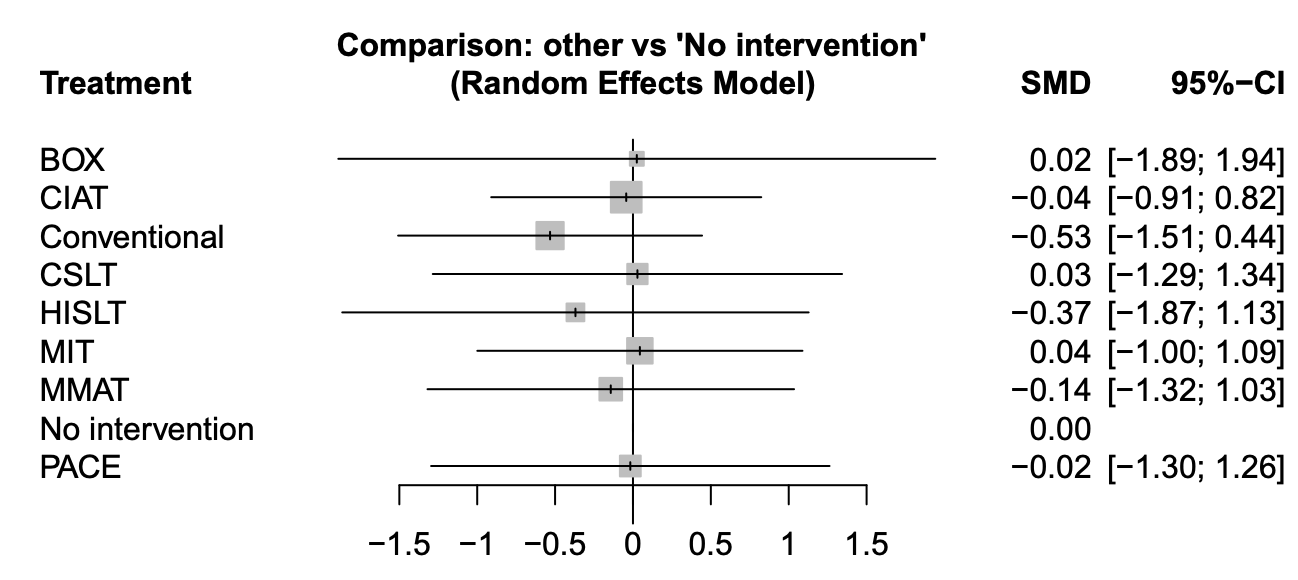


Supplementary Figure 28. Forest plots of all results showing the naming performance of all patients. Abbreviations: 95% CI, 95% credible interval; CIAT, constraint-induced aphasia therapy; CSLT, computerized speech and language therapy; HISLT, high-intensity speech and language therapy; MIT, melodic intonation therapy; MMAT, multimodality aphasia therapy; PACE, promoting aphasics’ communicative effectiveness; SMD, standard mean difference.


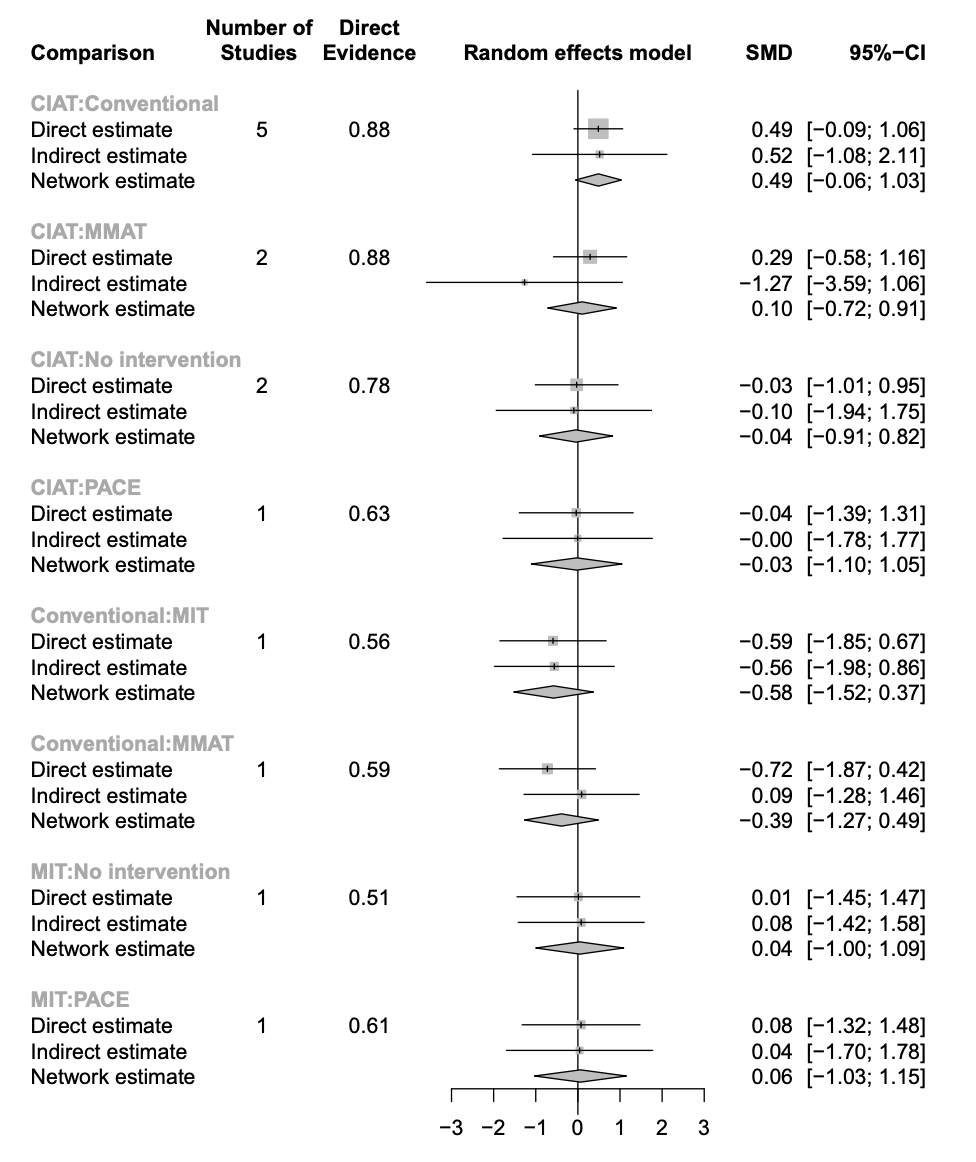


Supplementary Figure 29. Forest plots of pairwise and network meta-analyses showing the naming performance of all patients. Abbreviations: 95% CI, 95% credible interval; CIAT, constraint-induced aphasia therapy; MIT, melodic intonation therapy; MMAT, multimodality aphasia therapy; PACE, promoting aphasics’ communicative effectiveness; SMD, standard mean difference.


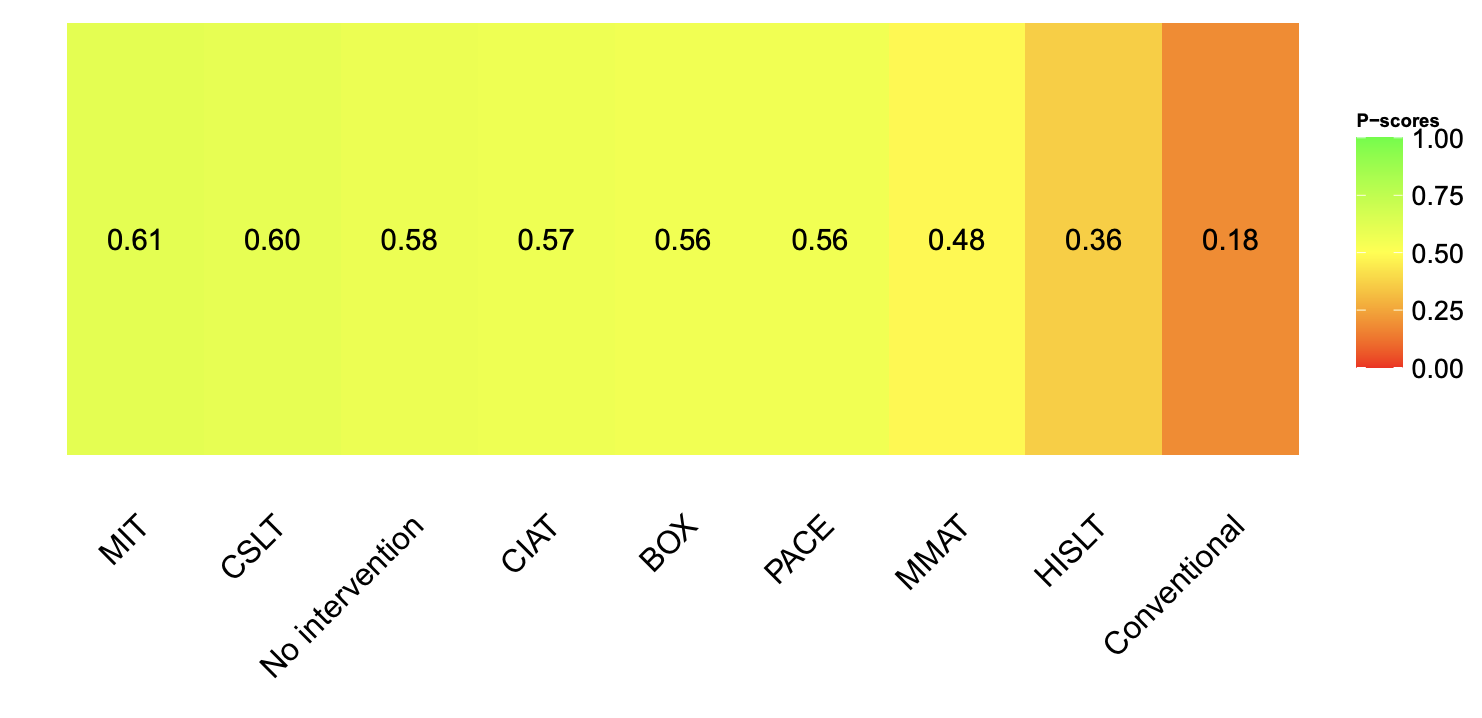


Supplementary Figure 30. Distribution of probabilities of effectiveness for each speech therapy showing the naming performance of all patients. Abbreviations: CIAT, constraint-induced aphasia therapy; CSLT, computerized speech and language therapy; HISLT, high-intensity speech and language therapy; MIT, melodic intonation therapy; MMAT, multimodality aphasia therapy; PACE, promoting aphasics’ communicative effectiveness.


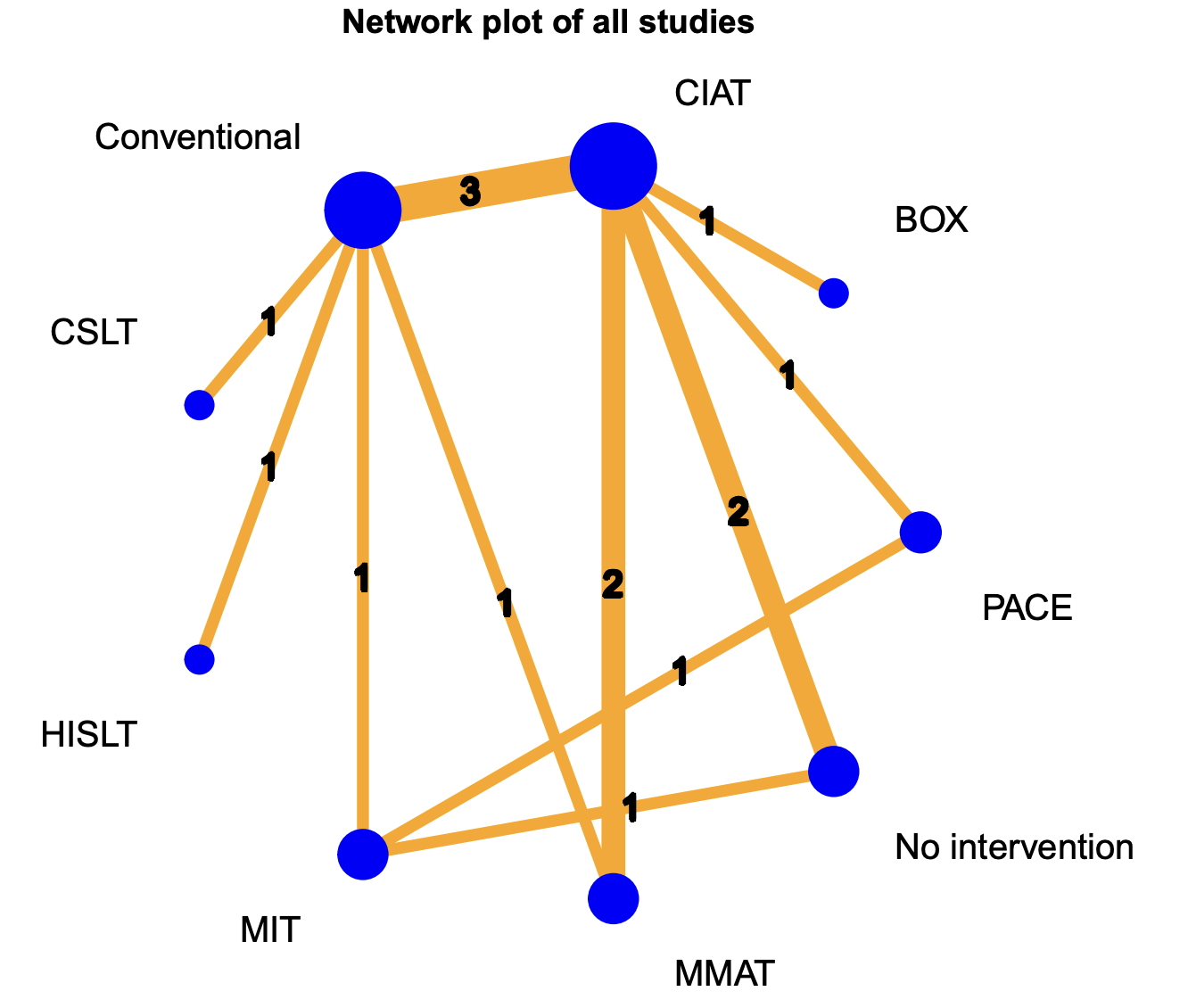


Supplementary Figure 31. Network plots of all studies showing the naming performance of patients in the chronic phase. Node size represents the total number of participants in each treatment arm, and edge width is proportional to the number of studies comparing the connected interventions. Abbreviations: CIAT, constraint-induced aphasia therapy; CSLT, computerized speech and language therapy; HISLT, high-intensity speech and language therapy; MIT, melodic intonation therapy; MMAT, multimodality aphasia therapy; PACE, promoting aphasics’ communicative effectiveness.


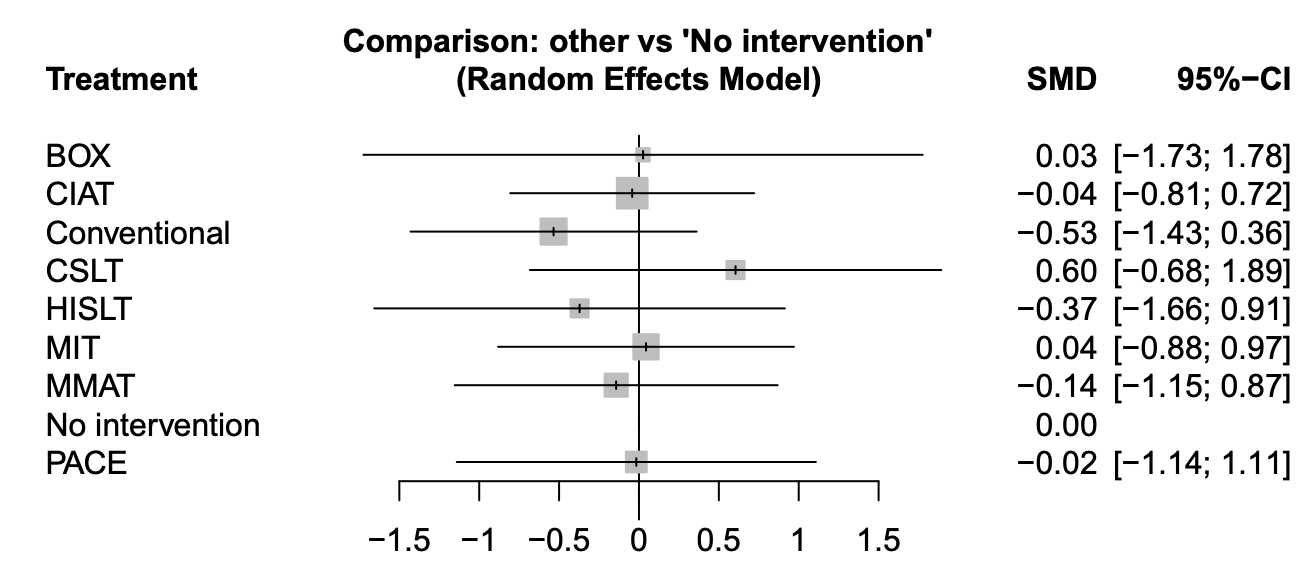


Supplementary Figure 32. Forest plots of all results showing the naming performance of patients in the chronic phase. Abbreviations: 95% CI, 95% credible interval; CIAT, constraint-induced aphasia therapy; CSLT, computerized speech and language therapy; HISLT, high-intensity speech and language therapy; MIT, melodic intonation therapy; MMAT, multimodality aphasia therapy; PACE, promoting aphasics’ communicative effectiveness; SMD, standard mean difference.


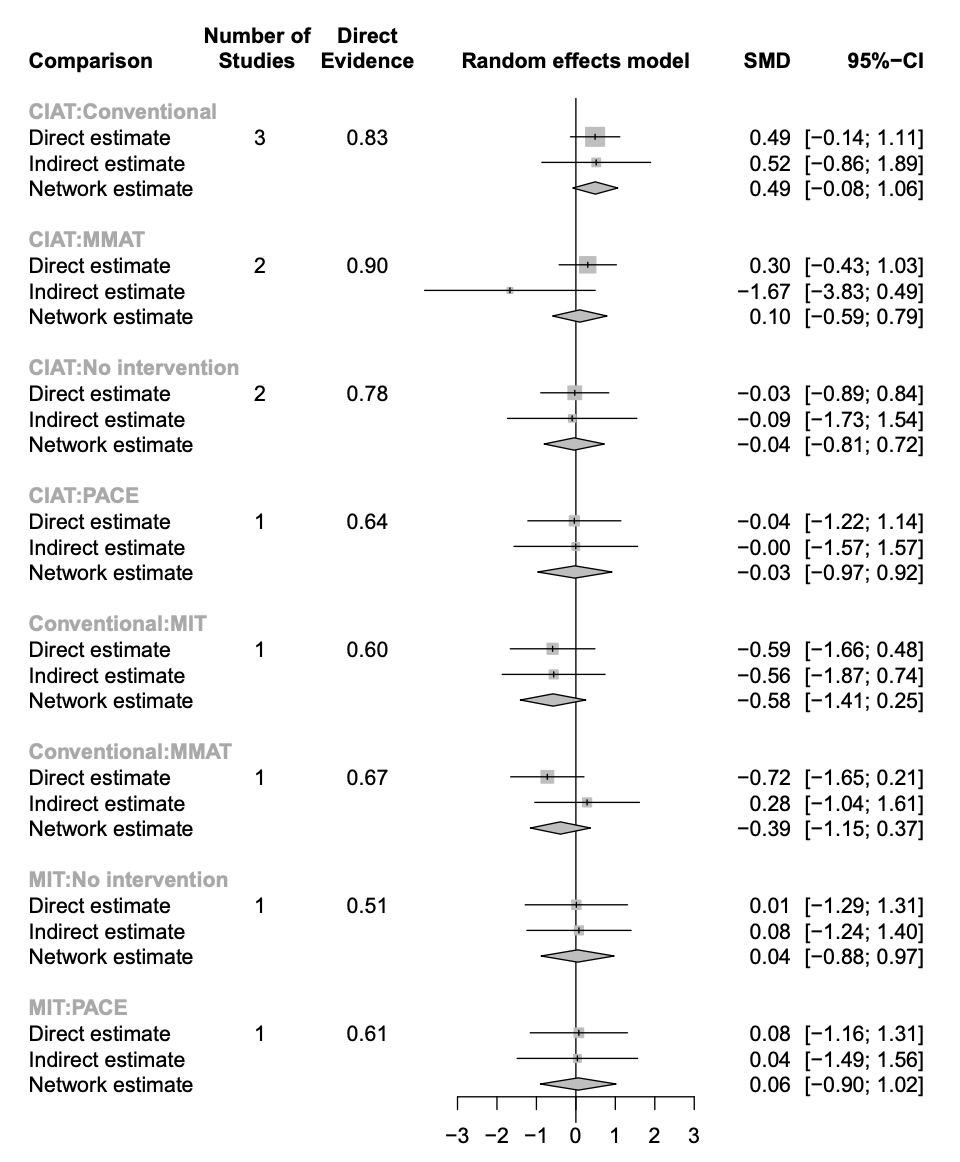


Supplementary Figure 33. Forest plots of pairwise and network meta-analyses showing the naming performance of patients in the chronic phase. Abbreviations: 95% CI, 95% credible interval; CIAT, constraint-induced aphasia therapy; MIT, melodic intonation therapy; MMAT, multimodality aphasia therapy; PACE, promoting aphasics’ communicative effectiveness; SMD, standard mean difference.


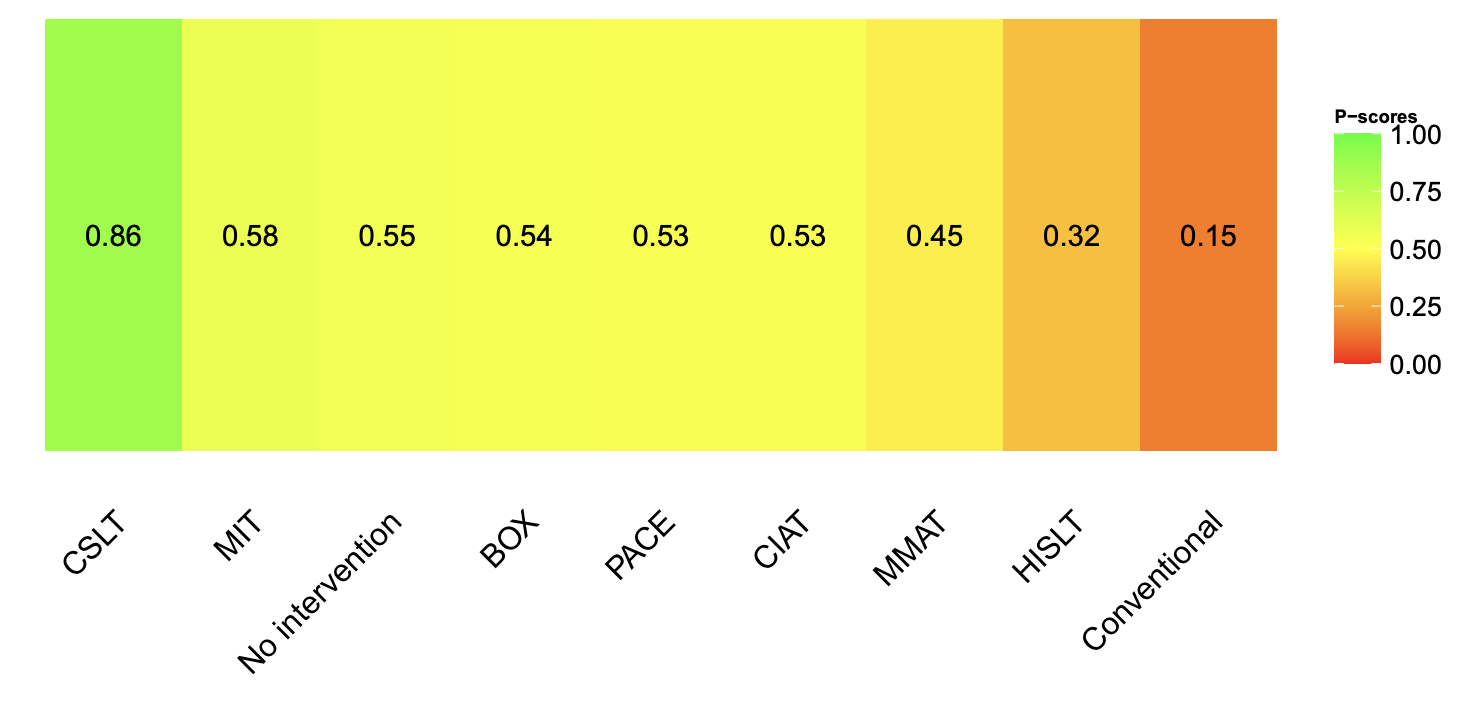


Supplementary Figure 34. Distribution of probabilities of effectiveness for each speech therapy showing the naming performance of patients in the chronic phase. Abbreviations: CIAT, constraint-induced aphasia therapy; CSLT, computerized speech and language therapy; HISLT, high-intensity speech and language therapy; MIT, melodic intonation therapy; MMAT, multimodality aphasia therapy; PACE, promoting aphasics’ communicative effectiveness.

**Supplementary Table Legends**

Supplementary Table 1. Characteristics of the Selected Randomized Controlled Trials.

Supplementary Table 2. Assessment of inconsistencies among studies regarding quality of life of all patients.

Supplementary Table 3. Assessment of quality of evidence using Confidence in Network meta-analysis (CINeMA) regarding quality of life of all patients.

Supplementary Table 4. Assessment of inconsistencies among studies regarding quality of life of patients in the chronic phase.

Supplementary Table 5. Assessment of quality of evidence using Confidence in Network meta-analysis (CINeMA) regarding quality of life of patients in the chronic phase.

Supplementary Table 6. Network meta-analysis results for fluency of all patients.

Supplementary Table 7. Assessment of inconsistencies among studies regarding fluency of all patients.

Supplementary Table 8. Assessment of quality of evidence using Confidence in Network meta-analysis (CINeMA) regarding fluency of all patients.

Supplementary Table 9. Network meta-analysis results for fluency of patients in the chronic phase.

Supplementary Table 10. Assessment of inconsistencies among studies regarding fluency of patients in the chronic phase.

Supplementary Table 11. Assessment of quality of evidence using Confidence in Network meta-analysis (CINeMA) regarding fluency of patients in the chronic phase.

Supplementary Table 12. Network meta-analysis results for comprehension of all patients.

Supplementary Table 13. Assessment of inconsistencies among studies regarding comprehension of all patients.

Supplementary Table 14. Assessment of quality of evidence using Confidence in Network meta-analysis (CINeMA) regarding comprehension of all patients.

Supplementary Table 15. Network meta-analysis results for comprehension of patients in the chronic phase.

Supplementary Table 16. Assessment of inconsistencies among studies regarding comprehension of patients in the chronic phase.

Supplementary Table 17. Assessment of quality of evidence using Confidence in Network meta-analysis (CINeMA) regarding comprehension of patients in the chronic phase.

Supplementary Table 18. Network meta-analysis results for repeat of all patients.

Supplementary Table 19. Assessment of quality of evidence using Confidence in Network meta-analysis (CINeMA) regarding repeat of all patients.

Supplementary Table 20. Network meta-analysis results for repeat of patients in the chronic phase.

Supplementary Table 21. Assessment of quality of evidence using Confidence in Network meta-analysis (CINeMA) regarding repeat of patients in the chronic phase.

Supplementary Table 22. Network meta-analysis results for naming of all patients.

Supplementary Table 23. Assessment of inconsistencies among studies regarding naming of all patients.

Supplementary Table 24. Assessment of quality of evidence using Confidence in Network meta-analysis (CINeMA) regarding naming of all patients.

Supplementary Table 25. Network meta-analysis results for naming of patients in the chronic phase.

Supplementary Table 26. Assessment of inconsistencies among studies regarding naming of patients in the chronic phase.

Supplementary Table 27. Assessment of quality of evidence using Confidence in Network meta-analysis (CINeMA) naming of patients in the chronic phase.

| Supplementary Table 1. Characteristics of the Selected Randomized Controlled Trials | | | | | | | | | |
| --- | --- | --- | --- | --- | --- | --- | --- | --- | --- |
| Author, year | Study design | Inclusion criteria | Intervention group | | | | | Longest follow-up | Outcome measurement |
|  |  |  | Group | Number of patients | Age (years), mean (SD) | Time since aphasia onset (months), mean (SD) | Intervention protocol |  |  |
| Breitenstein et al., 2017^42^ | Parallel | 1) Chronic aphasia lasting 6 months or more after an ischemic or hemorrhagic stroke; 2) age 18–70 years; 3) basic comprehension abilities and at least rudimentary attempts to verbally communicate; 4) the ability to follow simple instructions | Experimental group | 78 | 53·5 (9·0) | Mean (range) 43·0 (16·0–68·3) | HISLT: 10 hours per week of therapist-guided sessions plus 5 hours per week of self-managed training for three weeks | At the end of the interventions | SAPS - Language production, SAPS - Language comprehension, SAPS – Lexicon and SAQOL |
|  |  |  | Control group | 78 | 52·9 (10·2) | Mean (range) 27·0 (13·0–48·8) | No intervention |  |  |
| Ciccone et al., 2015^29^ | Parallel | 1) Acute stroke diagnosed within 48 hours of hospital admission; 2) Aphasia diagnosis (confirmed by score of <13/20 on the shortened Frenchay Aphasia Screening Test) and aphasia severity score of <93.7 on the AQ of WAB | Experimental group | 10 | 69.4 (15.0) | Days 5.6 (2.3) | CIAT: 45-60 min, 5 days a week for 20 sessions over 5 weeks (15-20 h total) | At the end of the interventions | SAQOL |
|  |  |  | Control group | 8 | 72.6 (14.1) | Days 4.8 (2.3) | Conventional therapy: 45-60 min, 5 days a week for 20 sessions over 5 weeks (15-20 h total) |  |  |
| Kurland et al., 2016^30^ | Parallel | 1) Diagnosed with poststroke aphasia; 2) Time since aphasia onset > 6 months | Experimental group | 12 | Mean (range) 68.6 (55-78) | 35.6 (6-142) | CIAT: 10 consecutive workdays × 3 h per day | At the end of the interventions | Cookie Theft Description, BDAE and BNT |
|  |  |  | Control group | 12 | 65 (46-81) | 17.8 (7-82) | PACE: 10 consecutive workdays × 3 h per day |  |  |
| Nenert et al., 2017^31^ | Parallel | Diagnosis of single ischemic stroke in the left middle cerebral artery | Experimental group | 11 | 58 (10.6) | Median (IQR) 60.2 (48.9) | CIAT: 10 daily sessions × 4 h | At the end and 3 months after the interventions | SFT, PPVT, BDAE and BNT |
|  |  |  | Control group | 8 | 50 (13.3) | Median (IQR) 41.9 (30) | No intervention |  |  |
| Pierce et al., 2023^33^ | Parallel | 1) Aged ≥17 years; 2) Chronic aphasia resulting from stroke (>6 months duration) confirmed by WAB-R-AQ < 93.8; 3); fluent in English prior to stroke | Experimental group | 10 | 60.5 (11.5) | Median (IQR) 46.5 (47.5) | CIAT: 2 h × 3 days × 5 weeks (30 h total) | At the end of the interventions | CIUs per minute, COMPARE naming battery and COAST |
|  |  |  | Control group | 16 | 66.1 (11.2) | Median (IQR) 30.5 (35.5) | MMAT: 2 h × 3 days × 5 weeks (30 h total) |  |  |
| Rose et al., 2022^26^ | Parallel | 1) Aged ≥18 years; 2) Chronic aphasia resulting from stroke of any type (>6 months duration) confirmed by an AQ of <93.8 on the WAB-R-AQ at the time of screening | Experimental group | 71 | Median (IQR) 63.93 (19.79) | Years, median (IQR) 2.41 (4.22) | CIAT: 3 h × 5 days per week × 2 weeks (30 h total) | At the end of the interventions | COMPARE naming battery and SAOQL |
|  |  |  | Experimental group | 75 | Median (IQR) 63.77 (21.02) | Years, median (IQR) 2.97 (3.81) | MMAT: 3 h × 5 days per week × 2 weeks (30 h total) |  |  |
|  |  |  | Control group | 70 | Median (IQR) 63.16 (14.10) | Years, median (IQR) 2.58 (2.87) | Conventional therapy |  |  |
| Sickert et al., 2013^35^ | Parallel | First-ever stroke with aphasia in the subacute stage (time since lesion onset 1-4 months poststroke) | Experimental group | 50 | 60.7 (41-81) | Days 36.7 (28-84) | CIAT: 2 h per day × 15 days | At the end of the interventions | AAT and CAL |
|  |  |  | Control group | 50 | 60.2 (34-84) | Days 32.9 (28-112) | Conventional therapy: 2 h per day × 15 days |  |  |
| Stahl et al., 2017^37^ | Crossover | Poststroke chronic nonfluent aphasia | Experimental group | 6 | 45.5 (10.6) | 45.7 (26.8) | CIAT treatment for 3.5 h per session for 6 consecutive working days, followed by 6-day washout period, then crossover to conventional therapy for 3.5 h per session for 6 consecutive working days | At the end of the interventions | ACT |
|  |  |  | Control group | 8 | 53.3 (11.9) | 109.4 (84.5) | Conventional therapy for 3.5 h per session for 6 consecutive working days, followed by 6-day washout period, then crossover to CIAT for 3.5 h per session for 6 consecutive working days |  |  |
| Szaflarski et al., 2015^27^ | Parallel | Chronic aphasia related to a single ischemic stroke in the left middle cerebral artery distribution | Experimental group | 14 | 57 (11) | Median (IQR) 38 (59) | CIAT: 4 h × 10 consecutive business days (40 h total) | At the end of the interventions | Peabody Picture Vocabulary Test III and BNT |
|  |  |  | Control group | 10 | 51 (13) | Median (IQR) 30 (58) | No intervention |  |  |
| Vuksanović et al., 2018^38^ | Crossover | Nonfluent poststroke aphasia | Experimental group | 8 | 61.4 (8.7) | Weeks 11.7 (13.7) | CIAT for 1 h × 5 times per week × 4 weeks, then crossover to conventional therapy 1 h × 5 times per week × 4 weeks | At the end of the interventions | BNT |
|  |  |  | Control group | 9 | 60.3 (10.5) | Weeks 15.8 (15.7) | Conventional therapy for 1 h × 5 times per week × 4 weeks, then crossover to CIAT for 1 h × 5 times per week × 4 weeks |  |  |
| Wilssens et al., 2015^39^ | Parallel | 1) Adult age; 2) Single and first-ever stroke in the left hemisphere confirmed by structural brain imaging; 3) Fluent aphasia with a combined semantic and phonological deficit | Experimental group | 5 | 63 (8) | 61 (48) | CIAT for 2-3-h sessions per day × 9 or 10 consecutive working days | At the end of the interventions | AAT and BNT |
|  |  |  | Control group | 4 | 71 (9) | 52 (25) | Semantic therapy (BOX therapy) for 2-3-h sessions per day × 9 or 10 consecutive working days |  |  |
| Woldag et al., 2016^40^ | Parallel | Acute aphasia after first-ever stroke | Experimental group | 20 | 71.3 (7.2) | Days 20.6 (10.9) | CIAT: 3 h × 10 workdays (30 h total) | At the end of the interventions | AAT and CAL |
|  |  |  | Control group | 20 | 70.3 (11.2) | Days 19.9 (10.0) | Conventional communication therapy: 3 h × 10 workdays (30 h total) |  |  |
| Palmer et al., 2019^32^ | Parallel | 1) Aged ≥18 years; 2) Aphasia diagnosis confirmed by a speech and language therapist after one or more strokes at least 4 months before randomization; 3) Difficulties finding words but able to perform a simple matching task on the StepByStep computer program | Experimental group | 83 | 64.9 (13.0) | Years 2.9 (2.9) | CSLT for 6 months | At the end of the interventions | picture naming test and COAST |
|  |  |  | Control group | 86 | 64.9 (13.0) | Years 2.8 (2.6) | Conventional communication for 6 months |  |  |
| Spaccavento et al., 2021^36^ | Parallel | 1) Aphasia resulting from unilateral left-hemisphere lesion; 2) Absence of premorbid cognitive impairment or mental health disorders | Experimental group | 13 | 57.38 (9.23) | Days 25.92 (25.99) | CSLT: 1 session per day of ±50 min × 5 days a week × 8 weeks | At the end of the interventions | AAT and QLQA |
|  |  |  | Control group | 9 | 64.11 (15.04) | Days 20 (10.66) | Conventional therapy: 1 session per day of ±50 min × 5 days a week × 8 weeks |  |  |
| Van Der Meulen et al., 2016^28^ | Parallel | 1) Aged 18-80 years; 2) >1 year poststroke | Experimental group | 10 | 58.1 (15.2) | 33.1 (19.4) | MIT: 5 h per week × 6 weeks | At the end of the interventions | Sabadel and AAT |
|  |  |  | Control group | 7 | 63.6 (12.7) | 42.6 (23.7) | No intervention |  |  |
| Raglio et al., 2016^34^ | Parallel | Patients with poststroke aphasia | Experimental group | 10 | 58.6 (12.4) | Years 3.4 (4.1) | MIT: individual 30-min sessions twice a week × 15 weeks (30 sessions total) | At the end of the interventions | AAT, Token test, BNT and SF-36 |
|  |  |  | Control group | 10 | 56.7 (10.5) | Years 3.8 (3.3) | PACE: individual 45-min sessions twice a week × 15 weeks (30 sessions total) |  |  |
| Zhang et al., 2021^41^ | Parallel | 1) Meeting the diagnostic criteria for nonfluent aphasia; 2) Time since aphasia onset ≥15, hospitalized patients; 3) Aged 18-70 years | Experimental group | 20 | 52.90 (9.08) | 2.57 (1.74) | MIT: 30 min × 5 times a week × 8 consecutive weeks | At the end of the interventions | BDAE |
|  |  |  | Control group | 20 | 54.05 (10.81) | 1.96 (1.38) | Conventional communication therapy: 30 min × 5 times a week × 8 consecutive weeks |  |  |
| Abbreviations:  AAT, Aachener Aphasia Test; ACT, Action Communication Test; BDAE, Boston Diagnostic Aphasia Examination; BNT, Boston Naming Test; CAL, Communicative Activity Log; CIAT, constraint-induced aphasia therapy; CIUs per minute, correct information units per minute; COAST, Communication Outcome after Stroke; COMPARE naming battery, Comprehensive Assessment for Person-Centered Rehabilitation naming battery; CSLT, computerized speech and language therapy; HISLT, high-intensity speech language therapy; IQR, interquartile range; MIT, melodic intonation therapy; MMAT, multimodality aphasia therapy; PACE, promoting aphasics’ communicative effectiveness; PPVT, Peabody Picture Vocabulary Test; QLQA, Quality of Life Questionnaire for Aphasics; SAQOL, Stroke and Aphasia Quality of Life Scale; SAPS, Scale for the Assessment of Phonological Skills; SD, standard deviation; SF-36, 36-Item Short Form Health Survey; SFT, Semantic Fluency Test. | | | | | | | | | |

| Supplementary Table 2. Assessment of inconsistencies among studies regarding quality of life of all patients. | | | | | | | | |
| --- | --- | --- | --- | --- | --- | --- | --- | --- |
| Comparison | Number of studies | Network meta-analysis | Direct comparison | Indirect comparison | Difference between direct and indirect comparisons | Lower limit of 95% CI | Upper limit of 95% CI | P value |
| CIAT: Conventional | 4 | 0.2865 | 0.2745 | 1.0155 | -0.7410 | -2.5373 | 1.0552 | 0.4188 |
| CIAT: MMAT | 2 | -0.4278 | -0.4061 | -0.6137 | 0.2076 | -0.7322 | 1.1475 | 0.6650 |
| MMAT: Conventional | 1 | 0.7143 | 0.7698 | 0.4950 | 0.2748 | -0.47678 | 1.0263 | 0.4736 |
| Abbreviations: 95% CI, 95% credible interval; CIAT, constraint-induced aphasia therapy; MMAT, multimodality aphasia therapy; SMD, standard mean difference. | | | | | | | | |

| Supplementary Table 3. Assessment of quality of evidence using Confidence in Network meta-analysis (CINeMA) regarding quality of life of all patients. | | | | | | | | | |
| --- | --- | --- | --- | --- | --- | --- | --- | --- | --- |
| Comparison | Number of studies | Within-study bias | Reporting bias | Indirectness | Imprecision | Heterogeneity | Incoherence | Confidence rating | Reason(s) for downgrading |
| CIAT: Conventional | 4 | No concerns | Low risk | No concerns | Some concerns | Major concerns | No concerns | Very low | [Imprecision and Heterogeneity] |
| CIAT: MMAT | 2 | No concerns | Low risk | No concerns | Some concerns | No concerns | No concerns | Moderate | [Imprecision] |
| Conventional: CSLT | 2 | No concerns | Low risk | No concerns | Major concerns | No concerns | No concerns | Low | [Imprecision] |
| Conventional: HISLT | 1 | No concerns | Low risk | No concerns | Major concerns | No concerns | No concerns | Low | [Imprecision] |
| Conventional: MMAT | 1 | No concerns | Low risk | No concerns | Some concerns | No concerns | No concerns | Moderate | [Imprecision] |
| CIAT: CSLT | 0 | No concerns | Low risk | No concerns | Major concerns | No concerns | No concerns | Low | [Imprecision] |
| CIAT: HISLT | 0 | No concerns | Low risk | No concerns | Major concerns | No concerns | No concerns | Low | [Imprecision] |
| CSLT: HISLT | 0 | No concerns | Low risk | No concerns | Major concerns | No concerns | No concerns | Low | [Imprecision] |
| CSLT: MMAT | 0 | No concerns | Low risk | No concerns | Some concerns | No concerns | No concerns | Moderate | [Imprecision] |
| HISLT: MMAT | 0 | No concerns | Low risk | No concerns | Some concerns | Major concerns | No concerns | Very low | [Imprecision and Heterogeneity] |
| Abbreviations: CIAT, constraint-induced aphasia therapy; CSLT, computerized speech and language therapy; HISLT, high-intensity speech and language therapy; MMAT, multimodality aphasia therapy. | | | | | | | | | |

| Supplementary Table 4. Assessment of inconsistencies among studies regarding quality of life of patients in the chronic phase. | | | | | | | | |
| --- | --- | --- | --- | --- | --- | --- | --- | --- |
| Comparison | Number of studies | Network meta-analysis | Direct comparison | Indirect comparison | Difference between direct and indirect comparisons | Lower limit of 95% CI | Upper limit of 95% CI | P value |
| CIAT: Conventional | 2 | 0.3949 | 0.3779 | 1.0154 | -0.6375 | -2.4433 | 1.1683 | 0.4889 |
| CIAT: MMAT | 2 | -0.3825 | -0.4061 | 0.1543 | -0.5605 | -2.0415 | 0.9205 | 0.4582 |
| MMAT: Conventional | 1 | 0.7774 | 0.7698 | 0.8439 | -0.0741 | -1.1232 | 0.9749 | 0.8897 |
| Abbreviations: 95% CI, 95% credible interval; CIAT, constraint-induced aphasia therapy; MMAT, multimodality aphasia therapy; SMD, standard mean difference. | | | | | | | | |

| Supplementary Table 5. Assessment of quality of evidence using Confidence in Network meta-analysis (CINeMA) regarding quality of life of patients in the chronic phase. | | | | | | | | | |
| --- | --- | --- | --- | --- | --- | --- | --- | --- | --- |
| Comparison | Number of studies | Within-study bias | Reporting bias | Indirectness | Imprecision | Heterogeneity | Incoherence | Confidence rating | Reason(s) for downgrading |
| CIAT: Conventional | 2 | No concerns | Low risk | No concerns | No concerns | Major concerns | No concerns | Low | [Heterogeneity] |
| CIAT: MMAT | 2 | No concerns | Low risk | No concerns | No concerns | Major concerns | No concerns | Low | [Heterogeneity] |
| Conventional: CSLT | 1 | No concerns | Low risk | No concerns | Major concerns | No concerns | No concerns | Low | [Imprecision] |
| Conventional: HISLT | 1 | No concerns | Low risk | No concerns | Major concerns | No concerns | No concerns | Low | [Imprecision] |
| Conventional: MMAT | 1 | No concerns | Low risk | No concerns | No concerns | Major concerns | No concerns | Low | [Heterogeneity] |
| CIAT: CSLT | 0 | No concerns | Low risk | No concerns | Major concerns | No concerns | No concerns | Low | [Imprecision] |
| CIAT: HISLT | 0 | No concerns | Low risk | No concerns | Major concerns | No concerns | No concerns | Low | [Imprecision] |
| CSLT: HISLT | 0 | No concerns | Low risk | No concerns | Major concerns | No concerns | No concerns | Low | [Imprecision] |
| CSLT: MMAT | 0 | No concerns | Low risk | No concerns | No concerns | Major concerns | No concerns | Low | [Heterogeneity] |
| HISLT: MMAT | 0 | No concerns | Low risk | No concerns | No concerns | Major concerns | No concerns | Low | [Heterogeneity] |
| Abbreviations: CIAT, constraint-induced aphasia therapy; CSLT, computerized speech and language therapy; HISLT, high-intensity speech and language therapy; MMAT, multimodality aphasia therapy. | | | | | | | | | |

| Supplementary Table 6. Network meta-analysis results for fluency of all patients. | | | | | | | |
| --- | --- | --- | --- | --- | --- | --- | --- |
| Pairwise meta-analysis | | | | | | | |
| **MIT** |  | -0.15 [-1.12; 0.82] |  | 0.34 [-0.29; 0.96] |  | 0.30 [-0.58; 1.19] |  |
| 0.04 [-0.56; 0.65] | **HISLT** |  |  | 0.17 [-0.14; 0.49] |  |  |  |
| 0.05 [-0.60; 0.70] | 0.01 [-0.68; 0.69] | **No intervention** | 0.06 [-0.55; 0.67] |  |  |  |  |
| 0.18 [-0.37; 0.73] | 0.14 [-0.34; 0.62] | 0.14 [-0.40; 0.67] | **CIAT** | -0.02 [-0.41; 0.38] | 0.17 [-0.62; 0.96] | 0.27 [-0.54; 1.07] |  |
| 0.21 [-0.30; 0.73] | 0.17 [-0.14; 0.49] | 0.17 [-0.45; 0.78] | 0.03 [-0.33; 0.40] | **Conventional** |  |  | 0.38 [-0.48; 1.24] |
| 0.35 [-0.61; 1.31] | 0.31 [-0.62; 1.24] | 0.30 [-0.65; 1.26] | 0.17 [-0.62; 0.96] | 0.14 [-0.74; 1.01] | **MMAT** |  |  |
| 0.38 [-0.28; 1.05] | 0.34 [-0.41; 1.10] | 0.34 [-0.46; 1.13] | 0.20 [-0.44; 0.85] | 0.17 [-0.52; 0.86] | 0.03 [-0.99; 1.05] | **PACE** |  |
| 0.59 [-0.41; 1.60] | 0.55 [-0.36; 1.47] | 0.55 [-0.51; 1.60] | 0.41 [-0.52; 1.34] | 0.38 [-0.48; 1.24] | 0.24 [-0.98; 1.47] | 0.21 [-0.89; 1.31] | **CSLT** |
| Network meta-analysis | | | | | | | |
| Data are expressed as SMD [95% CI]. Significant results are underscored. “-” means data not applicable.  Abbreviations: 95% CI, 95% credible interval; CIAT, constraint-induced aphasia therapy; CSLT, computerized speech and language therapy; HISLT, high-intensity speech and language therapy; MIT, melodic intonation therapy; MMAT, multimodality aphasia therapy; PACE, promoting aphasics’ communicative effectiveness; SMD, standard mean difference.  Note: The table presents two types of estimates. Results in the upper-right triangle (above the gray diagonal) represent the SMD from direct (pairwise) meta-analyses. Results in the lower-left triangle (below the gray diagonal) represent the SMD from the final network meta-analysis, which incorporates both direct and indirect evidence. | | | | | | | |

| Supplementary Table 7. Assessment of inconsistencies among studies regarding fluency of all patients. | | | | | | | | |
| --- | --- | --- | --- | --- | --- | --- | --- | --- |
| Comparison | Number of studies | Network meta-analysis | Direct comparison | Indirect comparison | Difference between direct and indirect comparisons | Lower limit of 95% CI | Upper limit of 95% CI | P value |
| CIAT: Conventional | 1 | 0.0319 | -0.0158 | 0.3653 | -0.3812 | -1.4883 | 0.7259 | 0.4997 |
| CIAT: No intervention | 2 | -0.1358 | -0.0582 | -0.4146 | 0.3564 | -0.9476 | 1.6604 | 0.5921 |
| CIAT: PACE | 1 | 0.2005 | 0.2661 | 0.0825 | 0.1836 | -1.1618 | 1.5291 | 0.7891 |
| Conventional: MIT | 1 | -0.2142 | -0.3355 | 0.0456 | -0.3812 | -1.4883 | 0.7259 | 0.4997 |
| MIT: No intervention | 1 | 0.0464 | -0.1498 | 0.2065 | -0.3564 | -1.6604 | 0.9476 | 0.5921 |
| MIT: PACE | 1 | 0.3828 | 0.3037 | 0.48739 | -0.1836 | -1.5291 | 1.1618 | 0.7891 |
| Abbreviations: 95% CI, 95% credible interval; CIAT, constraint-induced aphasia therapy; MIT, melodic intonation therapy; PACE, promoting aphasics’ communicative effectiveness. | | | | | | | | |

| Supplementary Table 8. Assessment of quality of evidence using Confidence in Network meta-analysis (CINeMA) regarding fluency of all patients. | | | | | | | | | |
| --- | --- | --- | --- | --- | --- | --- | --- | --- | --- |
| Comparison | Number of studies | Within-study bias | Reporting bias | Indirectness | Imprecision | Heterogeneity | Incoherence | Confidence rating | Reason(s) for downgrading |
| CIAT: Conventional | 1 | No concerns | Low risk | No concerns | Major concerns | No concerns | No concerns | Low | [Imprecision] |
| CIAT: MMAT | 1 | No concerns | Low risk | No concerns | Major concerns | No concerns | No concerns | Low | [Imprecision] |
| CIAT: No intervention | 2 | No concerns | Low risk | No concerns | Major concerns | No concerns | No concerns | Low | [Imprecision] |
| CIAT: PACE | 1 | No concerns | Low risk | No concerns | Major concerns | No concerns | No concerns | Low | [Imprecision] |
| Conventional: CSLT | 1 | No concerns | Low risk | No concerns | Major concerns | No concerns | No concerns | Low | [Imprecision] |
| Conventional: HISLT | 1 | No concerns | Low risk | No concerns | Major concerns | No concerns | No concerns | Low | [Imprecision] |
| Conventional: MIT | 1 | No concerns | Low risk | No concerns | Major concerns | No concerns | No concerns | Low | [Imprecision] |
| MIT: No intervention | 1 | No concerns | Low risk | No concerns | Major concerns | No concerns | No concerns | Low | [Imprecision] |
| MIT: PACE | 1 | No concerns | Low risk | No concerns | Major concerns | No concerns | No concerns | Low | [Imprecision] |
| CIAT: CSLT | 0 | No concerns | Low risk | No concerns | Major concerns | No concerns | No concerns | Low | [Imprecision] |
| CIAT: HISLT | 0 | No concerns | Low risk | No concerns | Major concerns | No concerns | No concerns | Low | [Imprecision] |
| CIAT: MIT | 0 | No concerns | Low risk | No concerns | Major concerns | No concerns | No concerns | Low | [Imprecision] |
| CSLT: HISLT | 0 | No concerns | Low risk | No concerns | Major concerns | No concerns | No concerns | Low | [Imprecision] |
| CSLT: MIT | 0 | No concerns | Low risk | No concerns | Major concerns | No concerns | No concerns | Low | [Imprecision] |
| CSLT: MMAT | 0 | No concerns | Low risk | No concerns | Major concerns | No concerns | No concerns | Low | [Imprecision] |
| CSLT: No intervention | 0 | No concerns | Low risk | No concerns | Major concerns | No concerns | No concerns | Low | [Imprecision] |
| CSLT: PACE | 0 | No concerns | Low risk | No concerns | Major concerns | No concerns | No concerns | Low | [Imprecision] |
| Conventional: MMAT | 0 | No concerns | Low risk | No concerns | Major concerns | No concerns | No concerns | Low | [Imprecision] |
| Conventional: No intervention | 0 | No concerns | Low risk | No concerns | Major concerns | No concerns | No concerns | Low | [Imprecision] |
| Conventional: PACE | 0 | No concerns | Low risk | No concerns | Major concerns | No concerns | No concerns | Low | [Imprecision] |
| HISLT: MIT | 0 | No concerns | Low risk | No concerns | Major concerns | No concerns | No concerns | Low | [Imprecision] |
| HISLT: MMAT | 0 | No concerns | Low risk | No concerns | Major concerns | No concerns | No concerns | Low | [Imprecision] |
| HISLT: No intervention | 0 | No concerns | Low risk | No concerns | Major concerns | No concerns | No concerns | Low | [Imprecision] |
| HISLT: PACE | 0 | No concerns | Low risk | No concerns | Major concerns | No concerns | No concerns | Low | [Imprecision] |
| MIT: MMAT | 0 | No concerns | Low risk | No concerns | Major concerns | No concerns | No concerns | Low | [Imprecision] |
| MMAT: No intervention | 0 | No concerns | Low risk | No concerns | Major concerns | No concerns | No concerns | Low | [Imprecision] |
| MMAT: PACE | 0 | No concerns | Low risk | No concerns | Major concerns | No concerns | No concerns | Low | [Imprecision] |
| No intervention: PACE | 0 | No concerns | Low risk | No concerns | Major concerns | No concerns | No concerns | Low | [Imprecision] |
| Abbreviations: 95% CI, 95% credible interval; CIAT, constraint-induced aphasia therapy; CSLT, computerized speech and language therapy; HISLT, high-intensity speech and language therapy; MIT, melodic intonation therapy; MMAT, multimodality aphasia therapy; PACE, promoting aphasics’ communicative effectiveness. | | | | | | | | | |

| Supplementary Table 9. Network meta-analysis results for fluency of patients in the chronic phase. | | | | | | |
| --- | --- | --- | --- | --- | --- | --- |
| Pairwise meta-analysis | | | | | | |
| **No intervention** | 0.06 [-0.55; 0.67] | 0.15 [-0.82; 1.12] |  |  |  |  |
| 0.08 [-0.49; 0.64] | **CIAT** |  |  | 0.17 [-0.62; 0.96] | 0.27 [-0.54; 1.07] |  |
| 0.11 [-0.68; 0.89] | 0.03 [-0.80; 0.86] | **MIT** |  |  | 0.30 [-0.58; 1.19] | 0.34 [-0.29; 0.96] |
| 0.27 [-0.78; 1.32] | 0.19 [-0.89; 1.27] | 0.16 [-0.54; 0.86] | **HISLT** |  |  | 0.17 [-0.14; 0.49] |
| 0.24 [-0.73; 1.22] | 0.17 [-0.62; 0.96] | 0.14 [-1.01; 1.28] | -0.02 [-1.37; 1.32] | **MMAT** |  |  |
| 0.37 [-0.43; 1.17] | 0.30 [-0.41; 1.00] | 0.27 [-0.48; 1.01] | 0.10 [-0.92; 1.13] | 0.13 [-0.93; 1.19] | **PACE** |  |
| 0.44 [-0.56; 1.44] | 0.37 [-0.67; 1.40] | 0.34 [-0.29; 0.96] | 0.17 [-0.14; 0.49] | 0.20 [-1.11; 1.50] | 0.07 [-0.90; 1.04] | **Conventional** |
| Network meta-analysis | | | | | | |
| Data are expressed as SMD [95% CI]. Significant results are underscored. “-” means data not applicable.  Abbreviations: 95% CI, 95% credible interval; CIAT, constraint-induced aphasia therapy; HISLT, high-intensity speech and language therapy; MIT, melodic intonation therapy; MMAT, multimodality aphasia therapy; PACE, promoting aphasics’ communicative effectiveness; SMD, standard mean difference.  Note: The table presents two types of estimates. Results in the upper-right triangle (above the gray diagonal) represent the SMD from direct (pairwise) meta-analyses. Results in the lower-left triangle (below the gray diagonal) represent the SMD from the final network meta-analysis, which incorporates both direct and indirect evidence. | | | | | | |

| Supplementary Table 10. Assessment of inconsistencies among studies regarding fluency of patients in the chronic phase. | | | | | | | | |
| --- | --- | --- | --- | --- | --- | --- | --- | --- |
| Comparison | Number of studies | Network meta-analysis | Direct comparison | Indirect comparison | Difference between direct and indirect comparisons | Lower limit of 95% CI | Upper limit of 95% CI | P value |
| CIAT: No intervention | 2 | -0.0757 | -0.0582 | -0.1874 | 0.12923 | -1.5239 | 1.7824 | 0.8782 |
| CIAT: PACE | 1 | 0.2967 | 0.2661 | 0.3953 | -0.1292 | -1.7824 | 1.5239 | 0.8782 |
| MIT: No intervention | 1 | -0.1055 | -0.1498 | -0.0205 | -0.1292 | -1.7824 | 1.5239 | 0.8782 |
| MIT: PACE | 1 | 0.2669 | 0.3037 | 0.1745 | 0.1292 | -1.5239 | 1.7824 | 0.8782 |
| Abbreviations: 95% CI, 95% credible interval; CIAT, constraint-induced aphasia therapy; MIT, melodic intonation therapy; PACE, promoting aphasics’ communicative effectiveness. | | | | | | | | |

| Supplementary Table 11. Assessment of quality of evidence using Confidence in Network meta-analysis (CINeMA) regarding fluency of patients in the chronic phase. | | | | | | | | | |
| --- | --- | --- | --- | --- | --- | --- | --- | --- | --- |
| Comparison | Number of studies | Within-study bias | Reporting bias | Indirectness | Imprecision | Heterogeneity | Incoherence | Confidence rating | Reason(s) for downgrading |
| CIAT: MMAT | 1 | No concerns | Low risk | No concerns | Major concerns | No concerns | No concerns | Low | [Imprecision] |
| CIAT: No intervention | 2 | No concerns | Low risk | No concerns | Major concerns | No concerns | No concerns | Low | [Imprecision] |
| CIAT: PACE | 1 | No concerns | Low risk | No concerns | Major concerns | No concerns | No concerns | Low | [Imprecision] |
| Conventional: HISLT | 1 | No concerns | Low risk | No concerns | Major concerns | No concerns | No concerns | Low | [Imprecision] |
| Conventional: MIT | 1 | No concerns | Low risk | No concerns | Major concerns | No concerns | No concerns | Low | [Imprecision] |
| MIT: PACE | 1 | No concerns | Low risk | No concerns | Major concerns | No concerns | No concerns | Low | [Imprecision] |
| CIAT: Conventional | 0 | No concerns | Low risk | No concerns | Major concerns | No concerns | No concerns | Low | [Imprecision] |
| CIAT: HISLT | 0 | No concerns | Low risk | No concerns | Major concerns | No concerns | No concerns | Low | [Imprecision] |
| CIAT: MIT | 0 | No concerns | Low risk | No concerns | Major concerns | No concerns | No concerns | Low | [Imprecision] |
| Conventional: MMAT | 0 | No concerns | Low risk | No concerns | Major concerns | No concerns | No concerns | Low | [Imprecision] |
| Conventional: No intervention | 0 | No concerns | Low risk | No concerns | Major concerns | No concerns | No concerns | Low | [Imprecision] |
| Conventional: PACE | 0 | No concerns | Low risk | No concerns | Major concerns | No concerns | No concerns | Low | [Imprecision] |
| HISLT: MIT | 0 | No concerns | Low risk | No concerns | Major concerns | No concerns | No concerns | Low | [Imprecision] |
| HISLT: MMAT | 0 | No concerns | Low risk | No concerns | Major concerns | No concerns | No concerns | Low | [Imprecision] |
| HISLT: No intervention | 0 | No concerns | Low risk | No concerns | Major concerns | No concerns | No concerns | Low | [Imprecision] |
| HISLT: PACE | 0 | No concerns | Low risk | No concerns | Major concerns | No concerns | No concerns | Low | [Imprecision] |
| MIT: MMAT | 0 | No concerns | Low risk | No concerns | Major concerns | No concerns | No concerns | Low | [Imprecision] |
| MIT: No intervention | 0 | No concerns | Low risk | No concerns | Major concerns | No concerns | No concerns | Low | [Imprecision] |
| MMAT: No intervention | 0 | No concerns | Low risk | No concerns | Major concerns | No concerns | No concerns | Low | [Imprecision] |
| MMAT: PACE | 0 | No concerns | Low risk | No concerns | Major concerns | No concerns | No concerns | Low | [Imprecision] |
| No intervention: PACE | 0 | No concerns | Low risk | No concerns | Major concerns | No concerns | No concerns | Low | [Imprecision] |
| Abbreviations: CIAT, constraint-induced aphasia therapy; HISLT, high-intensity speech and language therapy; MIT, melodic intonation therapy; MMAT, multimodality aphasia therapy; PACE, promoting aphasics’ communicative effectiveness. | | | | | | | | | |

| Supplementary Table 12. Network meta-analysis results for comprehension of all patients. | | | | | | | |
| --- | --- | --- | --- | --- | --- | --- | --- |
| Pairwise meta-analysis | | | | | | | |
| **MIT** |  |  | 0.00 [-0.95; 0.95] | -0.41 [-1.45; 0.64] |  |  | 0.82 [ 0.08; 1.57] |
| 0.01 [-0.76; 0.78] | **HISLT** |  |  |  |  |  | 0.39 [-0.10; 0.88] |
| -0.14 [-1.67; 1.38] | -0.15 [-1.69; 1.38] | **BOX** |  |  | 0.46 [-0.94; 1.85] |  |  |
| 0.17 [-0.56; 0.90] | 0.16 [-0.74; 1.06] | 0.31 [-1.25; 1.88] | **PACE** |  | -0.00 [-0.88; 0.88] |  |  |
| 0.23 [-0.49; 0.94] | 0.22 [-0.62; 1.05] | 0.37 [-1.14; 1.88] | 0.06 [-0.80; 0.92] | **No intervention** | -0.17 [-0.83; 0.49] |  |  |
| 0.31 [-0.31; 0.93] | 0.30 [-0.33; 0.94] | 0.46 [-0.94; 1.85] | 0.14 [-0.56; 0.85] | 0.09 [-0.50; 0.67] | **CIAT** |  | -0.05 [-0.48; 0.38] |
| 0.50 [-0.61; 1.60] | 0.49 [-0.56; 1.53] | 0.64 [-1.08; 2.36] | 0.33 [-0.87; 1.53] | 0.27 [-0.88; 1.42] | 0.18 [-0.83; 1.20] | **CSLT** | -0.09 [-1.02; 0.83] |
| 0.40 [-0.20; 1.00] | 0.39 [-0.10; 0.88] | 0.55 [-0.91; 2.00] | 0.23 [-0.53; 0.99] | 0.18 [-0.50; 0.85] | 0.09 [-0.32; 0.50] | -0.09 [-1.02; 0.83] | **Conventional** |
| Network meta-analysis | | | | | | | |
| Data are expressed as SMD [95% CI]. Significant results are underscored. “-” means data not applicable.  Abbreviations: 95% CI, 95% credible interval; CIAT, constraint-induced aphasia therapy; CSLT, computerized speech and language therapy; HISLT, high-intensity speech and language therapy; MIT, melodic intonation therapy; PACE, promoting aphasics’ communicative effectiveness; SMD, standard mean difference.  Note: The table presents two types of estimates. Results in the upper-right triangle (above the gray diagonal) represent the SMD from direct (pairwise) meta-analyses. Results in the lower-left triangle (below the gray diagonal) represent the SMD from the final network meta-analysis, which incorporates both direct and indirect evidence. | | | | | | | |

| Supplementary Table 13. Assessment of inconsistencies among studies regarding comprehension of all patients. | | | | | | | | |
| --- | --- | --- | --- | --- | --- | --- | --- | --- |
| Comparison | Number of studies | Network meta-analysis | Direct comparison | Indirect comparison | Difference between direct and indirect comparisons | Lower limit of 95% CI | Upper limit of 95% CI | P value |
| CIAT: Conventional | 2 | 0.0899 | -0.0522 | 1.1247 | -1.1770 | -2.4211 | 0.0670 | 0.0636 |
| CIAT: No intervention | 2 | -0.0851 | 0.1678 | -1.0132 | 1.1811 | -0.2508 | 2.6131 | 0.1059 |
| CIAT: PACE | 1 | -0.1435 | 0 | -0.4031 | 0.4031 | -1.0751 | 1.8814 | 0.5929 |
| Conventional: MIT | 1 | -0.4005 | -0.8249 | 0.3521 | -1.1770 | -2.4211 | 0.0670 | 0.0636 |
| MIT: No intervention | 1 | 0.2254 | -0.4051 | 0.7760 | -1.1811 | -2.6131 | 0.2508 | 0.1059 |
| MIT: PACE | 1 | 0.1671 | 0 | 0.4031 | -0.4031 | -1.8814 | 1.0751 | 0.5929 |
| Abbreviations: 95% CI, 95% credible interval; CIAT, constraint-induced aphasia therapy; MIT, melodic intonation therapy; PACE, promoting aphasics’ communicative effectiveness. | | | | | | | | |

| Supplementary Table 14. Assessment of quality of evidence using Confidence in Network meta-analysis (CINeMA) regarding comprehension of all patients. | | | | | | | | | |
| --- | --- | --- | --- | --- | --- | --- | --- | --- | --- |
| Comparison | Number of studies | Within-study bias | Reporting bias | Indirectness | Imprecision | Heterogeneity | Incoherence | Confidence rating | Reason(s) for downgrading |
| BOX: CIAT | 1 | No concerns | Low risk | No concerns | Major concerns | No concerns | Some concerns | Very low | [Imprecision and Incoherence] |
| CIAT: Conventional | 2 | No concerns | Low risk | No concerns | Major concerns | No concerns | No concerns | Low | [Imprecision] |
| CIAT: No intervention | 2 | No concerns | Low risk | No concerns | Major concerns | No concerns | No concerns | Low | [Imprecision] |
| CIAT: PACE | 1 | No concerns | Low risk | No concerns | Major concerns | No concerns | No concerns | Low | [Imprecision] |
| Conventional: CSLT | 1 | No concerns | Low risk | No concerns | Major concerns | No concerns | Some concerns | Very low | [Imprecision and Incoherence] |
| Conventional: HISLT | 1 | No concerns | Low risk | No concerns | Major concerns | No concerns | Some concerns | Very low | [Imprecision and Incoherence] |
| Conventional: MIT | 1 | No concerns | Low risk | No concerns | Major concerns | No concerns | Major concerns | Very low | [Imprecision and Incoherence] |
| MIT: No intervention | 1 | No concerns | Low risk | No concerns | Major concerns | No concerns | No concerns | Low | [Imprecision] |
| MIT: PACE | 1 | No concerns | Low risk | No concerns | Major concerns | No concerns | No concerns | Low | [Imprecision] |
| BOX: CSLT | 0 | No concerns | Low risk | No concerns | Major concerns | No concerns | Some concerns | Very low | [Imprecision and Incoherence] |
| BOX: Conventional | 0 | No concerns | Low risk | No concerns | Major concerns | No concerns | Some concerns | Very low | [Imprecision and Incoherence] |
| BOX: HISLT | 0 | No concerns | Low risk | No concerns | Major concerns | No concerns | Some concerns | Very low | [Imprecision and Incoherence] |
| BOX: MIT | 0 | No concerns | Low risk | No concerns | Major concerns | No concerns | Some concerns | Very low | [Imprecision and Incoherence] |
| BOX: No intervention | 0 | No concerns | Low risk | No concerns | Major concerns | No concerns | Some concerns | Very low | [Imprecision and Incoherence] |
| BOX: PACE | 0 | No concerns | Low risk | No concerns | Major concerns | No concerns | Some concerns | Very low | [Imprecision and Incoherence] |
| CIAT: CSLT | 0 | No concerns | Low risk | No concerns | Major concerns | No concerns | Some concerns | Very low | [Imprecision and Incoherence] |
| CIAT: HISLT | 0 | No concerns | Low risk | No concerns | Major concerns | No concerns | Some concerns | Very low | [Imprecision and Incoherence] |
| CIAT: MIT | 0 | No concerns | Low risk | No concerns | Major concerns | No concerns | Some concerns | Very low | [Imprecision and Incoherence] |
| CSLT: HISLT | 0 | No concerns | Low risk | No concerns | Major concerns | No concerns | Some concerns | Very low | [Imprecision and Incoherence] |
| CSLT: MIT | 0 | No concerns | Low risk | No concerns | Major concerns | No concerns | Some concerns | Very low | [Imprecision and Incoherence] |
| CSLT: No intervention | 0 | No concerns | Low risk | No concerns | Major concerns | No concerns | Some concerns | Very low | [Imprecision and Incoherence] |
| CSLT: PACE | 0 | No concerns | Low risk | No concerns | Major concerns | No concerns | Some concerns | Very low | [Imprecision and Incoherence] |
| Conventional: No intervention | 0 | No concerns | Low risk | No concerns | Major concerns | No concerns | Some concerns | Very low | [Imprecision and Incoherence] |
| Conventional: PACE | 0 | No concerns | Low risk | No concerns | Major concerns | No concerns | Some concerns | Very low | [Imprecision and Incoherence] |
| HISLT: MIT | 0 | No concerns | Low risk | No concerns | Major concerns | No concerns | Some concerns | Very low | [Imprecision and Incoherence] |
| HISLT: No intervention | 0 | No concerns | Low risk | No concerns | Major concerns | No concerns | Some concerns | Very low | [Imprecision and Incoherence] |
| HISLT: PACE | 0 | No concerns | Low risk | No concerns | Major concerns | No concerns | Some concerns | Very low | [Imprecision and Incoherence] |
| No intervention: PACE | 0 | No concerns | Low risk | No concerns | Major concerns | No concerns | Some concerns | Very low | [Imprecision and Incoherence] |
| Abbreviations: CIAT, constraint-induced aphasia therapy; CSLT, computerized speech and language therapy; HISLT, high-intensity speech and language therapy; MIT, melodic intonation therapy; PACE, promoting aphasics’ communicative effectiveness. | | | | | | | | | |

| Supplementary Table 15. Network meta-analysis results for comprehension of patients in the chronic phase. | | | | | | |
| --- | --- | --- | --- | --- | --- | --- |
| Pairwise meta-analysis | | | | | | |
| **BOX** | 0.46 [-0.89; 1.80] | . |  |  |  |  |
| 0.46 [-0.89; 1.80] | **CIAT** | 0.17 [-0.44; 0.78] | 0.00 [-0.80; 0.80] |  |  |  |
| 0.55 [-0.91; 2.01] | 0.09 [-0.47; 0.66] | **No intervention** |  | 0.41 [-0.57; 1.38] |  |  |
| 0.59 [-0.93; 2.11] | 0.13 [-0.57; 0.83] | 0.04 [-0.76; 0.84] | **PACE** | -0.00 [-0.88; 0.88] |  |  |
| 0.75 [-0.83; 2.33] | 0.30 [-0.53; 1.12] | 0.20 [-0.59; 0.99] | 0.16 [-0.58; 0.90] | **MIT** |  | **0.82 [ 0.18; 1.47]** |
| 1.19 [-0.55; 2.92] | 0.73 [-0.37; 1.83] | 0.64 [-0.43; 1.71] | 0.60 [-0.44; 1.63] | 0.43 [-0.29; 1.16] | **HISLT** | **0.39 [ 0.07; 0.71]** |
| 1.58 [-0.13; 3.28] | 1.12 [ 0.07; 2.17] | 1.03 [ 0.01; 2.05] | 0.99 [ 0.00; 1.97] | 0.82 [ 0.18; 1.47] | 0.39 [ 0.07; 0.71] | **Conventional** |
| Network meta-analysis | | | | | | |
| Data are expressed as SMD [95% CI]. Significant results are underscored. “-” means data not applicable.  Abbreviations: 95% CI, 95% credible interval; CIAT, constraint-induced aphasia therapy; CSLT, computerized speech and language therapy; HISLT, high-intensity speech and language therapy; MIT, melodic intonation therapy; PACE, promoting aphasics’ communicative effectiveness; SMD, standard mean difference.  Note: The table presents two types of estimates. Results in the upper-right triangle (above the gray diagonal) represent the SMD from direct (pairwise) meta-analyses. Results in the lower-left triangle (below the gray diagonal) represent the SMD from the final network meta-analysis, which incorporates both direct and indirect evidence. | | | | | | |

| Supplementary Table 16. Assessment of inconsistencies among studies regarding comprehension of patients in the chronic phase. | | | | | | | | |
| --- | --- | --- | --- | --- | --- | --- | --- | --- |
| Comparison | Number of studies | Network meta-analysis | Direct comparison | Indirect comparison | Difference between direct and indirect comparisons | Lower limit of 95% CI | Upper limit of 95% CI | P value |
| CIAT: No intervention | 2 | 0.0925 | 0.1704 | -0.4051 | 0.5755 | -1.0783 | 2.2294 | 0.4952 |
| CIAT: PACE | 1 | 0.1347 | 0 | 0.5755 | -0.5755 | -2.2294 | 1.0783 | 0.4952 |
| MIT: No intervention | 1 | -0.2037 | -0.4051 | 0.1704 | -0.5755 | -2.2294 | 1.0783 | 0.4952 |
| MIT: PACE | 1 | -0.1616 | 0 | -0.5755 | 0.5755 | -1.0783 | 2.2294 | 0.4952 |
| Abbreviations: 95% CI, 95% credible interval; CIAT, constraint-induced aphasia therapy; MIT, melodic intonation therapy; PACE, promoting aphasics’ communicative effectiveness. | | | | | | | | |

| Supplementary Table 17. Assessment of quality of evidence using Confidence in Network meta-analysis (CINeMA) regarding comprehension of patients in the chronic phase. | | | | | | | | | |
| --- | --- | --- | --- | --- | --- | --- | --- | --- | --- |
| Comparison | Number of studies | Within-study bias | Reporting bias | Indirectness | Imprecision | Heterogeneity | Incoherence | Confidence rating | Reason(s) for downgrading |
| BOX: CIAT | 1 | No concerns | Low risk | No concerns | Major concerns | No concerns | No concerns | Low | [Imprecision] |
| CIAT: No intervention | 2 | No concerns | Low risk | No concerns | Major concerns | No concerns | No concerns | Low | [Imprecision] |
| CIAT: PACE | 1 | No concerns | Low risk | No concerns | Major concerns | No concerns | No concerns | Low | [Imprecision] |
| Conventional: HISLT | 1 | No concerns | Low risk | No concerns | No concerns | Major concerns | No concerns | Low | [Heterogeneity] |
| Conventional: MIT | 1 | No concerns | Low risk | No concerns | No concerns | Major concerns | No concerns | Low | [Heterogeneity] |
| MIT: No intervention | 1 | No concerns | Low risk | No concerns | Major concerns | No concerns | No concerns | Low | [Imprecision] |
| MIT: PACE | 1 | No concerns | Low risk | No concerns | Major concerns | No concerns | No concerns | Low | [Imprecision] |
| BOX: Conventional | 0 | No concerns | Low risk | No concerns | Major concerns | No concerns | No concerns | Low | [Imprecision] |
| BOX: HISLT | 0 | No concerns | Low risk | No concerns | Major concerns | No concerns | No concerns | Low | [Imprecision] |
| BOX: MIT | 0 | No concerns | Low risk | No concerns | Major concerns | No concerns | No concerns | Low | [Imprecision] |
| BOX: No intervention | 0 | No concerns | Low risk | No concerns | Major concerns | No concerns | No concerns | Low | [Imprecision] |
| BOX: PACE | 0 | No concerns | Low risk | No concerns | Major concerns | No concerns | No concerns | Low | [Imprecision] |
| CIAT: Conventional | 0 | No concerns | Low risk | No concerns | No concerns | Major concerns | No concerns | Low | [Heterogeneity] |
| CIAT: HISLT | 0 | No concerns | Low risk | No concerns | Major concerns | No concerns | No concerns | Low | [Imprecision] |
| CIAT: MIT | 0 | No concerns | Low risk | No concerns | Major concerns | No concerns | No concerns | Low | [Imprecision] |
| Conventional: No intervention | 0 | No concerns | Low risk | No concerns | No concerns | Major concerns | No concerns | Low | [Heterogeneity] |
| Conventional: PACE | 0 | No concerns | Low risk | No concerns | Major concerns | No concerns | No concerns | Low | [Imprecision] |
| HISLT: MIT | 0 | No concerns | Low risk | No concerns | Major concerns | No concerns | No concerns | Low | [Imprecision] |
| HISLT: No intervention | 0 | No concerns | Low risk | No concerns | Major concerns | No concerns | No concerns | Low | [Imprecision] |
| HISLT: PACE | 0 | No concerns | Low risk | No concerns | Major concerns | No concerns | No concerns | Low | [Imprecision] |
| No intervention: PACE | 0 | No concerns | Low risk | No concerns | Major concerns | No concerns | No concerns | Low | [Imprecision] |
| Abbreviations: CIAT, constraint-induced aphasia therapy; CSLT, computerized speech and language therapy; HISLT, high-intensity speech and language therapy; MIT, melodic intonation therapy; PACE, promoting aphasics’ communicative effectiveness. | | | | | | | | | |

| Supplementary Table 18. Network meta-analysis results for repeat of all patients. | | | | | | |
| --- | --- | --- | --- | --- | --- | --- |
| Pairwise meta-analysis | | | | | | |
| **MIT** | -0.06 [-1.03; 0.90] | 0.95 [ 0.29; 1.61] |  |  |  |  |
| -0.06 [-1.03; 0.90] | **No intervention** |  |  |  |  |  |
| 0.95 [ 0.29; 1.61] | 1.02 [-0.15; 2.18] | **Conventional** |  | 0.08 [-0.25; 0.42] |  | 0.58 [-0.29; 1.45] |
| 0.99 [-0.10; 2.08] | 1.05 [-0.40; 2.51] | 0.04 [-0.83; 0.90] | **PACE** | 0.05 [-0.76; 0.85] |  |  |
| 1.04 [ 0.30; 1.77] | 1.10 [-0.12; 2.31] | 0.08 [-0.25; 0.42] | 0.05 [-0.76; 0.85] | **CIAT** | 0.29 [-1.03; 1.62] |  |
| 1.33 [-0.19; 2.85] | 1.39 [-0.41; 3.19] | 0.38 [-0.99; 1.75] | 0.34 [-1.21; 1.89] | 0.29 [-1.03; 1.62] | **BOX** |  |
| 1.53 [ 0.44; 2.62] | 1.59 [ 0.14; 3.05] | 0.58 [-0.29; 1.45] | 0.54 [-0.69; 1.77] | 0.49 [-0.44; 1.42] | 0.20 [-1.42; 1.82] | **CSLT** |
| Network meta-analysis | | | | | | |
| Data are expressed as SMD [95% CI]. Significant results are underscored. “-” means data not applicable.  Abbreviations: 95% CI, 95% credible interval; CIAT, constraint-induced aphasia therapy; CSLT, computerized speech and language therapy; MIT, melodic intonation therapy; PACE, promoting aphasics’ communicative effectiveness; SMD, standard mean difference.  Note: The table presents two types of estimates. Results in the upper-right triangle (above the gray diagonal) represent the SMD from direct (pairwise) meta-analyses. Results in the lower-left triangle (below the gray diagonal) represent the SMD from the final network meta-analysis, which incorporates both direct and indirect evidence. | | | | | | |

| Supplementary Table 19. Assessment of quality of evidence using Confidence in Network meta-analysis (CINeMA) regarding repeat of all patients. | | | | | | | | | |
| --- | --- | --- | --- | --- | --- | --- | --- | --- | --- |
| Comparison | Number of studies | Within-study bias | Reporting bias | Indirectness | Imprecision | Heterogeneity | Incoherence | Confidence rating | Reason(s) for downgrading |
| BOX: CIAT | 1 | No concerns | Low risk | No concerns | Major concerns | No concerns | No concerns | Low | [Imprecision] |
| CIAT: Conventional | 2 | No concerns | Low risk | No concerns | Major concerns | No concerns | No concerns | Low | [Imprecision] |
| CIAT: PACE | 1 | No concerns | Low risk | No concerns | Major concerns | No concerns | No concerns | Low | [Imprecision] |
| Conventional: CSLT | 1 | No concerns | Low risk | No concerns | Major concerns | No concerns | No concerns | Low | [Imprecision] |
| Conventional: MIT | 1 | No concerns | Low risk | No concerns | Major concerns | No concerns | No concerns | Low | [Imprecision] |
| MIT: No intervention | 1 | No concerns | Low risk | No concerns | Major concerns | No concerns | No concerns | Low | [Imprecision] |
| BOX: CSLT | 0 | No concerns | Low risk | No concerns | Major concerns | No concerns | No concerns | Low | [Imprecision] |
| BOX: Conventional | 0 | No concerns | Low risk | No concerns | Major concerns | No concerns | No concerns | Low | [Imprecision] |
| BOX: MIT | 0 | No concerns | Low risk | No concerns | Major concerns | No concerns | No concerns | Low | [Imprecision] |
| BOX: No intervention | 0 | No concerns | Low risk | No concerns | Major concerns | No concerns | No concerns | Low | [Imprecision] |
| BOX: PACE | 0 | No concerns | Low risk | No concerns | Major concerns | No concerns | No concerns | Low | [Imprecision] |
| CIAT: CSLT | 0 | No concerns | Low risk | No concerns | Major concerns | No concerns | No concerns | Low | [Imprecision] |
| CIAT: MIT | 0 | No concerns | Low risk | No concerns | Major concerns | No concerns | No concerns | Low | [Imprecision] |
| CIAT: No intervention | 0 | No concerns | Low risk | No concerns | Major concerns | No concerns | No concerns | Low | [Imprecision] |
| CSLT: MIT | 0 | No concerns | Low risk | No concerns | Major concerns | No concerns | No concerns | Low | [Imprecision] |
| CSLT: No intervention | 0 | No concerns | Low risk | No concerns | Major concerns | No concerns | No concerns | Low | [Imprecision] |
| CSLT: PACE | 0 | No concerns | Low risk | No concerns | Major concerns | No concerns | No concerns | Low | [Imprecision] |
| Conventional: No intervention | 0 | No concerns | Low risk | No concerns | Major concerns | No concerns | No concerns | Low | [Imprecision] |
| Conventional: PACE | 0 | No concerns | Low risk | No concerns | Major concerns | No concerns | No concerns | Low | [Imprecision] |
| MIT: PACE | 0 | No concerns | Low risk | No concerns | Major concerns | No concerns | No concerns | Low | [Imprecision] |
| No intervention: PACE | 0 | No concerns | Low risk | No concerns | Major concerns | No concerns | No concerns | Low | [Imprecision] |
| Abbreviations: CIAT, constraint-induced aphasia therapy; CSLT, computerized speech and language therapy; MIT, melodic intonation therapy; PACE, promoting aphasics’ communicative effectiveness. | | | | | | | | | |

| Supplementary Table 20. Network meta-analysis results for repeat of patients in the chronic phase. | | | | | |
| --- | --- | --- | --- | --- | --- |
| Pairwise meta-analysis | | | | | |
| **MIT** | -0.06 [-1.03; 0.90] | 0.95 [ 0.29; 1.61] |  |  |  |
| -0.06 [-1.03; 0.90] | **No intervention** |  |  |  |  |
| 0.95 [ 0.29; 1.61] | 1.02 [-0.15; 2.18] | **Conventional** |  | 0.26 [-0.37; 0.88] |  |
| 1.16 [-0.05; 2.37] | 1.23 [-0.32; 2.77] | 0.21 [-0.80; 1.22] | **PACE** | 0.05 [-0.76; 0.85] |  |
| 1.21 [ 0.30; 2.11] | 1.27 [-0.05; 2.60] | 0.26 [-0.37; 0.88] | 0.05 [-0.76; 0.85] | **CIAT** | 0.29 [-1.03; 1.62] |
| 1.50 [-0.11; 3.11] | 1.57 [-0.31; 3.44] | 0.55 [-0.92; 2.02] | 0.34 [-1.21; 1.89] | 0.29 [-1.03; 1.62] | **BOX** |
| Network meta-analysis | | | | | |
| Data are expressed as SMD [95% CI]. Significant results are underscored. “-” means data not applicable.  Abbreviations: 95% CI, 95% credible interval; CIAT, constraint-induced aphasia therapy; MIT, melodic intonation therapy; PACE, promoting aphasics’ communicative effectiveness; SMD, standard mean difference.  Note: The table presents two types of estimates. Results in the upper-right triangle (above the gray diagonal) represent the SMD from direct (pairwise) meta-analyses. Results in the lower-left triangle (below the gray diagonal) represent the SMD from the final network meta-analysis, which incorporates both direct and indirect evidence. | | | | | |

| Supplementary Table 21. Assessment of quality of evidence using Confidence in Network meta-analysis (CINeMA) regarding repeat of patients in the chronic phase. | | | | | | | | | |
| --- | --- | --- | --- | --- | --- | --- | --- | --- | --- |
| Comparison | Number of studies | Within-study bias | Reporting bias | Indirectness | Imprecision | Heterogeneity | Incoherence | Confidence rating | Reason(s) for downgrading |
| BOX: CIAT | 1 | No concerns | Low risk | No concerns | Major concerns | No concerns | No concerns | Low | [Imprecision] |
| CIAT: Conventional | 1 | No concerns | Low risk | No concerns | Major concerns | No concerns | No concerns | Low | [Imprecision] |
| CIAT: PACE | 1 | No concerns | Low risk | No concerns | Major concerns | No concerns | No concerns | Low | [Imprecision] |
| Conventional: MIT | 1 | No concerns | Low risk | No concerns | Major concerns | No concerns | No concerns | Low | [Imprecision] |
| MIT: No intervention | 1 | No concerns | Low risk | No concerns | Major concerns | No concerns | No concerns | Low | [Imprecision] |
| BOX: Conventional | 0 | No concerns | Low risk | No concerns | Major concerns | No concerns | No concerns | Low | [Imprecision] |
| BOX: MIT | 0 | No concerns | Low risk | No concerns | Major concerns | No concerns | No concerns | Low | [Imprecision] |
| BOX: No intervention | 0 | No concerns | Low risk | No concerns | Major concerns | No concerns | No concerns | Low | [Imprecision] |
| BOX: PACE | 0 | No concerns | Low risk | No concerns | Major concerns | No concerns | No concerns | Low | [Imprecision] |
| CIAT: MIT | 0 | No concerns | Low risk | No concerns | Major concerns | No concerns | No concerns | Low | [Imprecision] |
| CIAT: No intervention | 0 | No concerns | Low risk | No concerns | Major concerns | No concerns | No concerns | Low | [Imprecision] |
| Conventional: No intervention | 0 | No concerns | Low risk | No concerns | Major concerns | No concerns | No concerns | Low | [Imprecision] |
| Conventional: PACE | 0 | No concerns | Low risk | No concerns | Major concerns | No concerns | No concerns | Low | [Imprecision] |
| MIT: PACE | 0 | No concerns | Low risk | No concerns | Major concerns | No concerns | No concerns | Low | [Imprecision] |
| No intervention: PACE | 0 | No concerns | Low risk | No concerns | Major concerns | No concerns | No concerns | Low | [Imprecision] |
| Abbreviations: CIAT, constraint-induced aphasia therapy; MIT, melodic intonation therapy; PACE, promoting aphasics’ communicative effectiveness. | | | | | | | | | |

| Supplementary Table 22. Network meta-analysis results for naming of all patients. | | | | | | | | |
| --- | --- | --- | --- | --- | --- | --- | --- | --- |
| Pairwise meta-analysis | | | | | | | | |
| **MIT** |  | 0.01 [-1.45; 1.47] |  |  | 0.08 [-1.32; 1.48] |  |  | 0.59 [-0.67; 1.85] |
| 0.02 [-1.28; 1.31] | **CSLT** |  |  |  |  |  |  | 0.56 [-0.32; 1.44] |
| 0.04 [-1.00; 1.09] | 0.03 [-1.29; 1.34] | **No intervention** | 0.03 [-0.95; 1.01] |  |  |  |  |  |
| 0.09 [-0.86; 1.04] | 0.07 [-0.96; 1.11] | 0.04 [-0.82; 0.91] | **CIAT** | -0.07 [-1.78; 1.64] | -0.04 [-1.39; 1.31] | 0.29 [-0.58; 1.16] |  | 0.49 [-0.09; 1.06] |
| 0.02 [-1.94; 1.98] | 0.00 [-1.99; 2.00] | -0.02 [-1.94; 1.89] | -0.07 [-1.78; 1.64] | **BOX** |  |  |  |  |
| 0.06 [-1.03; 1.15] | 0.05 [-1.40; 1.49] | 0.02 [-1.26; 1.30] | -0.03 [-1.10; 1.05] | 0.04 [-1.98; 2.06] | **PACE** |  |  |  |
| 0.19 [-1.02; 1.40] | 0.17 [-1.07; 1.41] | 0.14 [-1.03; 1.32] | 0.10 [-0.72; 0.91] | 0.17 [-1.73; 2.06] | 0.13 [-1.21; 1.46] | **MMAT** |  | 0.72 [-0.42; 1.87] |
| 0.41 [-1.06; 1.89] | 0.40 [-1.04; 1.83] | 0.37 [-1.13; 1.87] | 0.33 [-0.93; 1.59] | 0.39 [-1.73; 2.52] | 0.35 [-1.26; 1.96] | 0.23 [-1.21; 1.66] | **HISLT** | 0.16 [-0.97; 1.30] |
| 0.58 [-0.37; 1.52] | 0.56 [-0.32; 1.44] | 0.53 [-0.44; 1.51] | 0.49 [-0.06; 1.03] | 0.56 [-1.24; 2.35] | 0.52 [-0.63; 1.66] | 0.39 [-0.49; 1.27] | 0.16 [-0.97; 1.30] | **Conventional** |
| Network meta-analysis | | | | | | | | |
| Data are expressed as SMD [95% CI]. Significant results are underscored. “-” means data not applicable.  Abbreviations: 95% CI, 95% credible interval; CIAT, constraint-induced aphasia therapy; CSLT, computerized speech and language therapy; HISLT, high-intensity speech and language therapy; MIT, melodic intonation therapy; MMAT, multimodality aphasia therapy; PACE, promoting aphasics’ communicative effectiveness; SMD, standard mean difference.  Note: The table presents two types of estimates. Results in the upper-right triangle (above the gray diagonal) represent the SMD from direct (pairwise) meta-analyses. Results in the lower-left triangle (below the gray diagonal) represent the SMD from the final network meta-analysis, which incorporates both direct and indirect evidence. | | | | | | | | |

| Supplementary Table 23. Assessment of inconsistencies among studies regarding naming of all patients. | | | | | | | | |
| --- | --- | --- | --- | --- | --- | --- | --- | --- |
| Comparison | Number of studies | Network meta-analysis | Direct comparison | Indirect comparison | Difference between direct and indirect comparisons | Lower limit of 95% CI | Upper limit of 95% CI | P value |
| CIAT: Conventional | 5 | 0.4889 | 0.4853 | 0.5161 | -0.0307 | -1.7290 | 1.6674 | 0.9716 |
| CIAT: MMAT | 2 | 0.0992 | 0.2904 | -1.2665 | 1.5570 | -0.9261 | 4.0402 | 0.2190 |
| CIAT: No intervention | 2 | -0.0432 | -0.0284 | -0.0955 | 0.0671 | -2.0218 | 2.1561 | 0.9497 |
| CIAT: PACE | 1 | -0.0262 | -0.0390 | -0.0041 | -0.0349 | -2.2654 | 2.1955 | 0.9755 |
| Conventional: MIT | 1 | -0.5766 | -0.5899 | -0.5596 | -0.0303 | -1.9332 | 1.8726 | 0.9751 |
| Conventional: MMAT | 1 | -0.3896 | -0.7233 | 0.0885 | -0.8118 | -2.5933 | 0.9697 | 0.3717 |
| MIT: No intervention | 1 | 0.0444 | 0.0117 | 0.0788 | -0.0671 | -2.1561 | 2.0218 | 0.9497 |
| MIT: PACE | 1 | 0.0614 | 0.0752 | 0.04029 | 0.0349 | -2.1955 | 2.2654 | 0.9755 |
| Abbreviations: 95% CI, 95% credible interval; CIAT, constraint-induced aphasia therapy; MIT, melodic intonation therapy; MMAT, multimodality aphasia therapy; PACE, promoting aphasics’ communicative effectiveness. | | | | | | | | |

| Supplementary Table 24. Assessment of quality of evidence using Confidence in Network meta-analysis (CINeMA) regarding naming of all patients. | | | | | | | | | |
| --- | --- | --- | --- | --- | --- | --- | --- | --- | --- |
| Comparison | Number of studies | Within-study bias | Reporting bias | Indirectness | Imprecision | Heterogeneity | Incoherence | Confidence rating | Reason(s) for downgrading |
| BOX: CIAT | 1 | No concerns | Low risk | No concerns | Major concerns | No concerns | No concerns | Low | [Imprecision] |
| CIAT: Conventional | 5 | No concerns | Low risk | No concerns | Major concerns | No concerns | No concerns | Low | [Imprecision] |
| CIAT: MMAT | 2 | No concerns | Low risk | No concerns | Major concerns | No concerns | No concerns | Low | [Imprecision] |
| CIAT: No intervention | 2 | No concerns | Low risk | No concerns | Major concerns | No concerns | No concerns | Low | [Imprecision] |
| CIAT: PACE | 1 | No concerns | Low risk | No concerns | Major concerns | No concerns | No concerns | Low | [Imprecision] |
| Conventional: CSLT | 2 | No concerns | Low risk | No concerns | Major concerns | No concerns | No concerns | Low | [Imprecision] |
| Conventional: HISLT | 1 | No concerns | Low risk | No concerns | Major concerns | No concerns | No concerns | Low | [Imprecision] |
| Conventional: MIT | 1 | No concerns | Low risk | No concerns | Major concerns | No concerns | No concerns | Low | [Imprecision] |
| Conventional: MMAT | 1 | No concerns | Low risk | No concerns | Major concerns | No concerns | No concerns | Low | [Imprecision] |
| MIT: No intervention | 1 | No concerns | Low risk | No concerns | Major concerns | No concerns | No concerns | Low | [Imprecision] |
| MIT: PACE | 1 | No concerns | Low risk | No concerns | Major concerns | No concerns | No concerns | Low | [Imprecision] |
| BOX: CSLT | 0 | No concerns | Low risk | No concerns | Major concerns | No concerns | No concerns | Low | [Imprecision] |
| BOX: Conventional | 0 | No concerns | Low risk | No concerns | Major concerns | No concerns | No concerns | Low | [Imprecision] |
| BOX: HISLT | 0 | No concerns | Low risk | No concerns | Major concerns | No concerns | No concerns | Low | [Imprecision] |
| BOX: MIT | 0 | No concerns | Low risk | No concerns | Major concerns | No concerns | No concerns | Low | [Imprecision] |
| BOX: MMAT | 0 | No concerns | Low risk | No concerns | Major concerns | No concerns | No concerns | Low | [Imprecision] |
| BOX: No intervention | 0 | No concerns | Low risk | No concerns | Major concerns | No concerns | No concerns | Low | [Imprecision] |
| BOX: PACE | 0 | No concerns | Low risk | No concerns | Major concerns | No concerns | No concerns | Low | [Imprecision] |
| CIAT: CSLT | 0 | No concerns | Low risk | No concerns | Major concerns | No concerns | No concerns | Low | [Imprecision] |
| CIAT: HISLT | 0 | No concerns | Low risk | No concerns | Major concerns | No concerns | No concerns | Low | [Imprecision] |
| CIAT: MIT | 0 | No concerns | Low risk | No concerns | Major concerns | No concerns | No concerns | Low | [Imprecision] |
| CSLT: HISLT | 0 | No concerns | Low risk | No concerns | Major concerns | No concerns | No concerns | Low | [Imprecision] |
| CSLT: MIT | 0 | No concerns | Low risk | No concerns | Major concerns | No concerns | No concerns | Low | [Imprecision] |
| CSLT: MMAT | 0 | No concerns | Low risk | No concerns | Major concerns | No concerns | No concerns | Low | [Imprecision] |
| CSLT: No intervention | 0 | No concerns | Low risk | No concerns | Major concerns | No concerns | No concerns | Low | [Imprecision] |
| CSLT: PACE | 0 | No concerns | Low risk | No concerns | Major concerns | No concerns | No concerns | Low | [Imprecision] |
| Conventional: No intervention | 0 | No concerns | Low risk | No concerns | Major concerns | No concerns | No concerns | Low | [Imprecision] |
| Conventional: PACE | 0 | No concerns | Low risk | No concerns | Major concerns | No concerns | No concerns | Low | [Imprecision] |
| HISLT: MIT | 0 | No concerns | Low risk | No concerns | Major concerns | No concerns | No concerns | Low | [Imprecision] |
| HISLT: MMAT | 0 | No concerns | Low risk | No concerns | Major concerns | No concerns | No concerns | Low | [Imprecision] |
| HISLT: No intervention | 0 | No concerns | Low risk | No concerns | Major concerns | No concerns | No concerns | Low | [Imprecision] |
| HISLT: PACE | 0 | No concerns | Low risk | No concerns | Major concerns | No concerns | No concerns | Low | [Imprecision] |
| MIT: MMAT | 0 | No concerns | Low risk | No concerns | Major concerns | No concerns | No concerns | Low | [Imprecision] |
| MMAT: No intervention | 0 | No concerns | Low risk | No concerns | Major concerns | No concerns | No concerns | Low | [Imprecision] |
| MMAT: PACE | 0 | No concerns | Low risk | No concerns | Major concerns | No concerns | No concerns | Low | [Imprecision] |
| No intervention: PACE | 0 | No concerns | Low risk | No concerns | Major concerns | No concerns | No concerns | Low | [Imprecision] |
| Abbreviations: CIAT, constraint-induced aphasia therapy; CSLT, computerized speech and language therapy; HISLT, high-intensity speech and language therapy; MIT, melodic intonation therapy; MMAT, multimodality aphasia therapy; PACE, promoting aphasics’ communicative effectiveness. | | | | | | | | | |

| Supplementary Table 25. Network meta-analysis results for naming of patients in the chronic phase. | | | | | | | | |
| --- | --- | --- | --- | --- | --- | --- | --- | --- |
| Pairwise meta-analysis | | | | | | | | |
| **CSLT** |  |  |  |  |  |  |  | 1.14 [ 0.21; 2.06] |
| 0.56 [-0.68; 1.80] | **MIT** | 0.01 [-1.29; 1.31] |  | 0.08 [-1.16; 1.31] |  |  |  | 0.59 [-0.48; 1.66] |
| 0.60 [-0.68; 1.89] | 0.04 [-0.88; 0.97] | **No intervention** |  |  | 0.03 [-0.84; 0.89] |  |  |  |
| 0.58 [-1.34; 2.49] | 0.02 [-1.77; 1.80] | -0.03 [-1.78; 1.73] | **BOX** |  | 0.07 [-1.51; 1.64] |  |  |  |
| 0.62 [-0.76; 2.00] | 0.06 [-0.90; 1.02] | 0.02 [-1.11; 1.14] | 0.04 [-1.79; 1.88] | **PACE** | 0.04 [-1.14; 1.22] |  |  |  |
| 0.65 [-0.44; 1.73] | 0.09 [-0.76; 0.93] | 0.04 [-0.72; 0.81] | 0.07 [-1.51; 1.64] | 0.03 [-0.92; 0.97] | **CIAT** | 0.30 [-0.43; 1.03] |  | 0.49 [-0.14; 1.11] |
| 0.75 [-0.45; 1.95] | 0.19 [-0.85; 1.22] | 0.14 [-0.87; 1.15] | 0.17 [-1.55; 1.89] | 0.13 [-1.02; 1.27] | 0.10 [-0.59; 0.79] | **MMAT** |  | 0.72 [-0.21; 1.65] |
| 0.98 [-0.33; 2.28] | 0.42 [-0.82; 1.66] | 0.37 [-0.91; 1.66] | 0.40 [-1.52; 2.31] | 0.36 [-1.02; 1.73] | 0.33 [-0.75; 1.41] | 0.23 [-0.97; 1.42] | **HISLT** | 0.16 [-0.76; 1.08] |
| 1.14 [ 0.21; 2.06] | 0.58 [-0.25; 1.41] | 0.53 [-0.36; 1.43] | 0.56 [-1.12; 2.24] | 0.52 [-0.51; 1.54] | 0.49 [-0.08; 1.06] | 0.39 [-0.37; 1.15] | 0.16 [-0.76; 1.08] | **Conventional** |
| Network meta-analysis | | | | | | | | |
| Data are expressed as SMD [95% CI]. Significant results are underscored. “-” means data not applicable.  Abbreviations: 95% CI, 95% credible interval; CIAT, constraint-induced aphasia therapy; CSLT, computerized speech and language therapy; HISLT, high-intensity speech and language therapy; MIT, melodic intonation therapy; MMAT, multimodality aphasia therapy; PACE, promoting aphasics’ communicative effectiveness; SMD, standard mean difference.  Note: The table presents two types of estimates. Results in the upper-right triangle (above the gray diagonal) represent the SMD from direct (pairwise) meta-analyses. Results in the lower-left triangle (below the gray diagonal) represent the SMD from the final network meta-analysis, which incorporates both direct and indirect evidence. | | | | | | | | |

| Supplementary Table 26. Assessment of inconsistencies among studies regarding naming of patients in the chronic phase. | | | | | | | | |
| --- | --- | --- | --- | --- | --- | --- | --- | --- |
| Comparison | Number of studies | Network meta-analysis | Direct comparison | Indirect comparison | Difference between direct and indirect comparisons | Lower limit of 95% CI | Upper limit of 95% CI | P value |
| CIAT: Conventional | 3 | 0.4921 | 0.4873 | 0.5156 | -0.0283 | -1.5430 | 1.4863 | 0.9707 |
| CIAT: MMAT | 2 | 0.1004 | 0.3007 | -1.6715 | 1.9722 | -0.3096 | 4.2542 | 0.0902 |
| CIAT: No intervention | 2 | -0.0423 | -0.0280 | -0.0941 | 0.0661 | -1.7863 | 1.9185 | 0.9442 |
| CIAT: PACE | 1 | -0.0258 | -0.0390 | -0.0023 | -0.0366 | -1.9987 | 1.9253 | 0.9707 |
| Conventional: MIT | 1 | -0.5787 | -0.5899 | -0.5622 | -0.0277 | -1.7167 | 1.6612 | 0.9742 |
| Conventional: MMAT | 1 | -0.3917 | -0.7233 | 0.2846 | -1.0079 | -2.6289 | 0.6130 | 0.2229 |
| MIT: No intervention | 1 | 0.0441 | 0.0117 | 0.0778 | -0.0661 | -1.9185 | 1.7863 | 0.9442 |
| MIT: PACE | 1 | 0.0607 | 0.0752 | 0.0385 | 0.0366 | -1.9253 | 1.9987 | 0.9707 |
| Abbreviations: 95% CI, 95% credible interval; CIAT, constraint-induced aphasia therapy; MIT, melodic intonation therapy; MMAT, multimodality aphasia therapy; PACE, promoting aphasics’ communicative effectiveness. | | | | | | | | |

| Supplementary Table 27. Assessment of quality of evidence using Confidence in Network meta-analysis (CINeMA) naming of patients in the chronic phase. | | | | | | | | | |
| --- | --- | --- | --- | --- | --- | --- | --- | --- | --- |
| Comparison | Number of studies | Within-study bias | Reporting bias | Indirectness | Imprecision | Heterogeneity | Incoherence | Confidence rating | Reason(s) for downgrading |
| BOX: CIAT | 1 | No concerns | Low risk | No concerns | Major concerns | No concerns | Major concerns | Very low | [Imprecision and Incoherence] |
| CIAT: Conventional | 3 | No concerns | Low risk | No concerns | Major concerns | No concerns | No concerns | Low | [Imprecision] |
| CIAT: MMAT | 2 | No concerns | Low risk | No concerns | Major concerns | No concerns | No concerns | Low | [Imprecision] |
| CIAT: No intervention | 2 | No concerns | Low risk | No concerns | Major concerns | No concerns | No concerns | Low | [Imprecision] |
| CIAT: PACE | 1 | No concerns | Low risk | No concerns | Major concerns | No concerns | No concerns | Low | [Imprecision] |
| Conventional: CSLT | 1 | No concerns | Low risk | No concerns | No concerns | Major concerns | Major concerns | Very low | [Heterogeneity and Incoherence] |
| Conventional: HISLT | 1 | No concerns | Low risk | No concerns | Major concerns | No concerns | Major concerns | Very low | [Imprecision and Incoherence] |
| Conventional: MIT | 1 | No concerns | Low risk | No concerns | Major concerns | No concerns | No concerns | Low | [Imprecision] |
| Conventional: MMAT | 1 | No concerns | Low risk | No concerns | Major concerns | No concerns | No concerns | Low | [Imprecision] |
| MIT: No intervention | 1 | No concerns | Low risk | No concerns | Major concerns | No concerns | No concerns | Low | [Imprecision] |
| MIT: PACE | 1 | No concerns | Low risk | No concerns | Major concerns | No concerns | No concerns | Low | [Imprecision] |
| BOX: CSLT | 0 | No concerns | Low risk | No concerns | Major concerns | No concerns | Major concerns | Very low | [Imprecision and Incoherence] |
| BOX: Conventional | 0 | No concerns | Low risk | No concerns | Major concerns | No concerns | Major concerns | Very low | [Imprecision and Incoherence] |
| BOX: HISLT | 0 | No concerns | Low risk | No concerns | Major concerns | No concerns | Major concerns | Very low | [Imprecision and Incoherence] |
| BOX: MIT | 0 | No concerns | Low risk | No concerns | Major concerns | No concerns | Major concerns | Very low | [Imprecision and Incoherence] |
| BOX: MMAT | 0 | No concerns | Low risk | No concerns | Major concerns | No concerns | Major concerns | Very low | [Imprecision and Incoherence] |
| BOX: No intervention | 0 | No concerns | Low risk | No concerns | Major concerns | No concerns | Major concerns | Very low | [Imprecision and Incoherence] |
| BOX: PACE | 0 | No concerns | Low risk | No concerns | Major concerns | No concerns | Major concerns | Very low | [Imprecision and Incoherence] |
| CIAT: CSLT | 0 | No concerns | Low risk | No concerns | Major concerns | No concerns | Major concerns | Very low | [Imprecision and Incoherence] |
| CIAT: HISLT | 0 | No concerns | Low risk | No concerns | Major concerns | No concerns | Major concerns | Very low | [Imprecision and Incoherence] |
| CIAT: MIT | 0 | No concerns | Low risk | No concerns | Major concerns | No concerns | Major concerns | Very low | [Imprecision and Incoherence] |
| CSLT: HISLT | 0 | No concerns | Low risk | No concerns | Major concerns | No concerns | Major concerns | Very low | [Imprecision and Incoherence] |
| CSLT: MIT | 0 | No concerns | Low risk | No concerns | Major concerns | No concerns | Major concerns | Very low | [Imprecision and Incoherence] |
| CSLT: MMAT | 0 | No concerns | Low risk | No concerns | Major concerns | No concerns | Major concerns | Very low | [Imprecision and Incoherence] |
| CSLT: No intervention | 0 | No concerns | Low risk | No concerns | Major concerns | No concerns | Major concerns | Very low | [Imprecision and Incoherence] |
| CSLT: PACE | 0 | No concerns | Low risk | No concerns | Major concerns | No concerns | Major concerns | Very low | [Imprecision and Incoherence] |
| Conventional: No intervention | 0 | No concerns | Low risk | No concerns | Major concerns | No concerns | Major concerns | Very low | [Imprecision and Incoherence] |
| Conventional: PACE | 0 | No concerns | Low risk | No concerns | Major concerns | No concerns | Major concerns | Very low | [Imprecision and Incoherence] |
| HISLT: MIT | 0 | No concerns | Low risk | No concerns | Major concerns | No concerns | Major concerns | Very low | [Imprecision and Incoherence] |
| HISLT: MMAT | 0 | No concerns | Low risk | No concerns | Major concerns | No concerns | Major concerns | Very low | [Imprecision and Incoherence] |
| HISLT: No intervention | 0 | No concerns | Low risk | No concerns | Major concerns | No concerns | Major concerns | Very low | [Imprecision and Incoherence] |
| HISLT: PACE | 0 | No concerns | Low risk | No concerns | Major concerns | No concerns | Major concerns | Very low | [Imprecision and Incoherence] |
| MIT: MMAT | 0 | No concerns | Low risk | No concerns | Major concerns | No concerns | Major concerns | Very low | [Imprecision and Incoherence] |
| MMAT: No intervention | 0 | No concerns | Low risk | No concerns | Major concerns | No concerns | Major concerns | Very low | [Imprecision and Incoherence] |
| MMAT: PACE | 0 | No concerns | Low risk | No concerns | Major concerns | No concerns | Major concerns | Very low | [Imprecision and Incoherence] |
| No intervention: PACE | 0 | No concerns | Low risk | No concerns | Major concerns | No concerns | Major concerns | Very low | [Imprecision and Incoherence] |
| Abbreviations: CIAT, constraint-induced aphasia therapy; CSLT, computerized speech and language therapy; HISLT, high-intensity speech and language therapy; MIT, melodic intonation therapy; MMAT, multimodality aphasia therapy; PACE, promoting aphasics’ communicative effectiveness. | | | | | | | | | |
