# Supplementary material for: Speech therapy for poststroke aphasia: a network meta-analysis of randomized controlled trials
Source: PeerJ. 2026 Apr 15;14:e21118. doi: 10.7717/peerj.21118 (PMC13091577; doi:10.7717/peerj.21118)
Supplement: Supplemental Information 4 [file peerj-14-21118-s004.docx]

**Appendix**

Keywords used for identifying relevant articles in various electronic databases

| Database | Search terms |
| --- | --- |
| **PubMed (inception: 1782)**  **EMBASE (inception: 1947)**  **Cochrane Library (inception: 1971)** | Search for articles published from database inception to September 20, 2023. |
| #1 | Constraint |
| #2 | Aphasia |
| #3 | Language |
| #4 | #2 OR #3 |
| #5 | #1 AND #4 |
| #6 | CIAT |
| #7 | CILT |
| #8 | #5 OR #6 OR #7 |
| #9 | Centeredness |
| #10 | CTI |
| #11 | #9 OR #10 |
| #12 | Copy |
| #13 | Recall |
| #14 | CART |
| #15 | #12 AND #13 |
| #16 | #14 OR #15 |
| #17 | Visual |
| #18 | Communication |
| #19 | VIC |
| #20 | #17 AND #18 |
| #21 | #19 OR #20 |
| #22 | Visual |
| #23 | Action |
| #24 | VAT |
| #25 | #22 AND #23 |
| #26 | #24 OR #25 |
| #27 | Functional |
| #28 | Communication |
| #29 | FCT |
| #30 | #27 AND #28 |
| #31 | #29 OR #30 |
| #32 | Promoting |
| #33 | Communicative |
| #34 | PACE |
| #35 | #32 AND #33 |
| #36 | #34 OR #35 |
| #37 | Melod* |
| #38 | Intonation |
| #39 | MIT |
| #40 | #37 OR #38 OR #39 |
| #41 | Intensive |
| #42 | Language |
| #43 | ILAT |
| #44 | #41 AND #42 |
| #45 | #43 OR #44 |
| #46 | Multi-modality |
| #47 | M-MAT |
| #48 | #46 OR #47 |
| #49 | Phonological |
| #50 | PCA |
| #51 | #49 OR #50 |
| #52 | Elaboration |
| #53 | RET |
| #54 | #52 OR #53 |
| #55 | Semantic |
| #56 | SFA |
| #57 | #55 OR #56 |
| #58 | Script |
| #59 | Sentence |
| #60 | SPPA |
| #61 | #59 OR #60 |
| #62 | Verb |
| #63 | VNeST |
| #64 | #62 OR #63 |
| #65 | Retrieval |
| #66 | Oral |
| #67 | Read* |
| #68 | MOR |
| #69 | #66 OR #67 OR #68 |
| #70 | Conversation* |
| #71 | SPPARC |
| #72 | #70 OR #71 |
| #73 | Augment* |
| #74 | AAC |
| #75 | #73 OR #74 |
| #76 | Gestur* |
| #77 | Reciprocal |
| #78 | Scaffolding |
| #79 | #77 AND #78 |
| #80 | #8 OR #11 OR #16 OR #21 OR #26 OR #31 OR #36 OR #40 OR #45 OR #48 OR #51 OR #54 OR #57 OR #58 OR #61 OR #64 OR #65 OR #69 OR #72 OR #75 OR #76 OR #79 |
| #81 | Aphasia* |
| #82 | Aphasia [MeSH] (Pubmed)  Aphasia/exp (EMBASE)  [Aphasia] explode all trees (Cochrane Library) |
| #83 | Anomi* |
| #84 | Linguistic* |
| #85 | Language* |
| #86 | Speech |
| #87 | #81 OR #82 OR #83 OR #84 OR #85 OR #86 |
| #88 | Ischem* |
| #89 | Hemorrhage* |
| #90 | Infarct* |
| #91 | #88 OR #89 OR #90 |
| #92 | Cerebral |
| #93 | Brain |
| #94 | #92 OR #93 |
| #95 | #91 AND #94 |
| #96 | Stroke |
| #97 | Cerebrovascular |
| #98 | CVA |
| #99 | Stroke [MeSH] (Pubmed)  Stroke/exp (EMBASE)  [Stroke] explode all trees (Cochrane Library) |
| #100 | #95 OR #96 OR #97 OR #98 OR #99 |
| #101 | #80 AND #87 AND #100 |
| Filter | Randomized controlled trial (Pubmed and EMBASE)  Random* (Additional search term for Cochrane Library) |
