# Supplementary material for: Speech therapy for poststroke aphasia: a network meta-analysis of randomized controlled trials
Source: PeerJ. 2026 Apr 15;14:e21118. doi: 10.7717/peerj.21118 (PMC13091577; doi:10.7717/peerj.21118)
Supplement: Supplemental Information 5 [file peerj-14-21118-s005.docx]

The audience it is intended for:

This systematic review and network meta-analysis was conducted to address a critical knowledge gap in clinical neurorehabilitation. While numerous speech therapies exist for post-stroke aphasia, there is a lack of comprehensive, direct comparisons to guide evidence-based practice. This study synthesizes the existing randomized controlled trial literature to compare the relative effectiveness of different named therapeutic protocols, aiming to identify which interventions are most effective for improving patients' overall communicative ability. The findings are intended for a broad, interdisciplinary audience, including clinical practitioners such as speech-language pathologists, neurologists, and physiatrists, as well as academic researchers in the fields of rehabilitation science, evidence-based medicine, and the neurosciences.
